# Both indirect maternal and direct fetal genetic effects reflect the observational relationship between higher birth weight and lower adult bone mass

**DOI:** 10.1186/s12916-022-02531-w

**Published:** 2022-10-04

**Authors:** Jiang-Wei Xia, Lin Zhang, Jin Li, Cheng-Da Yuan, Xiao-Wei Zhu, Yu Qian, Saber Khederzadeh, Jia-Xuan Gu, Lin Xu, Jian-Hua Gao, Ke-Qi Liu, David Karasik, Shu-Yang Xie, Guo-Bo Chen, Hou-Feng Zheng

**Affiliations:** 1 College of Life Sciences, Zhejiang University, Hangzhou, 310058 China; 2 Diseases & Population (DaP) Geninfo Lab, School of Life Sciences, Westlake University, 18 Shilongshan Road, Xihu District, Zhejiang, 310024 Hangzhou China; 3 Westlake Laboratory of Life Sciences and Biomedicine, 18 Shilongshan Road, Cloud Town, Xihu District, Zhejiang, 310024 Hangzhou China; 4 Hangzhou Women’s Hospital (Hangzhou Maternity and Child Health Care Hospital), Hangzhou, China; 5 Department of Dermatology, Hangzhou Hospital of Traditional Chinese Medicine, Zhejiang, 310007 Hangzhou China; 6 WBBC Shandong Center, Binzhou Medical University, Yantai, Shandong China; 7 WBBC Jiangxi Center, Jiangxi Medical College, Shangrao, Jiangxi China; 8 Azrieli Faculty of Medicine, Bar-Ilan University, Safed, Israel; 9 Clinical Research Institute, Zhejiang Provincial People’s Hospital, Hangzhou Medical College, Hangzhou, China

**Keywords:** Birth weight, Bone mineral density, Fetal genetic effects, Maternal genetic effects, Observational analysis, Mendelian randomization

## Abstract

**Background:**

Birth weight is considered not only to undermine future growth, but also to induce lifelong diseases; the aim of this study is to explore the relationship between birth weight and adult bone mass.

**Methods:**

We performed multivariable regression analyses to assess the association of birth weight with bone parameters measured by dual-energy X-ray absorptiometry (DXA) and by quantitative ultrasound (QUS), independently. We also implemented a systemic Mendelian randomization (MR) analysis to explore the causal association between them with both fetal-specific and maternal-specific instrumental variables.

**Results:**

In the observational analyses, we found that higher birth weight could increase the adult bone area (lumbar spine, *β*-coefficient= 0.17, *P* < 2.00 × 10^−16^; lateral spine, *β*-coefficient = 0.02, *P* = 0.04), decrease bone mineral content-adjusted bone area (BMCadjArea) (lumbar spine, *β*-coefficient= − 0.01, *P* = 2.27 × 10^−14^; lateral spine, *β*-coefficient = − 0.05, *P* = 0.001), and decrease adult bone mineral density (BMD) (lumbar spine, *β*-coefficient = − 0.04, *P* = 0.007; lateral spine; *β*-coefficient = − 0.03, *P* = 0.02; heel, *β*-coefficient = − 0.06, *P* < 2.00 × 10^−16^), and we observed that the effect of birth weight on bone size was larger than that on BMC. In MR analyses, the higher fetal-specific genetically determined birth weight was identified to be associated with higher bone area (lumbar spine; *β*-coefficient = 0.15, *P* = 1.26 × 10^−6^, total hip, *β*-coefficient = 0.15, *P* = 0.005; intertrochanteric area, *β*-coefficient = 0.13, *P* = 0.0009; trochanter area, *β*-coefficient = 0.11, *P* = 0.03) but lower BMD (lumbar spine, *β*-coefficient = − 0.10, *P* = 0.01; lateral spine, *β*-coefficient = − 0.12, *P* = 0.0003, and heel *β*-coefficient = − 0.11, *P =* 3.33 × 10^−13^). In addition, we found that the higher maternal-specific genetically determined offspring birth weight was associated with lower offspring adult heel BMD (*β*-coefficient = − 0.001, *P* = 0.04).

**Conclusions:**

The observational analyses suggested that higher birth weight was associated with the increased adult bone area but decreased BMD. By leveraging the genetic instrumental variables with maternal- and fetal-specific effects on birth weight, the observed relationship could be reflected by both the direct fetal and indirect maternal genetic effects.

**Supplementary Information:**

The online version contains supplementary material available at 10.1186/s12916-022-02531-w.

## Background

Osteoporosis is a common skeletal disease characterized by the loss of bone mass and the risk of brittle fracture. It has become a major global public health problem, affecting about 200 million people around the world [1]. Osteoporosis could be clinically diagnosed by measuring the bone mineral density (BMD), which is a highly heritable trait [2]. Some modifiable environmental factors such as body weight [3], physical activity [4], sleep behavior [5], and inflammatory disease [6] could have an impact on bone mass gain and the development of osteoporosis. Other non-modifiable factors included sex, age, and genetic factors. The genome-wide association studies (GWASs) have discovered hundreds of genetic loci that are associated with BMD, osteoporosis, and fracture in the past decade [7]. However, there is yet a considerable proportion of the variance in bone mass that cannot be explained by known genetic and environmental determinants, e.g., the role of the growth during the prenatal period on adult osteoporosis was often underestimated.

The Developmental Origins of Health and Disease (DOHaD) hypothesis was initially proposed in the 1990s [8], which proposed that the growth during the prenatal period might play a critical role in an individual’s short- and long-term health [9]. Although the relationship between birth weight and risk of osteoporosis in later life has been investigated in observational studies, the results were controversial. Some studies detected a positive association between birth weight and bone mass [10–13], but some other studies found an inverse association [14–16]; these conflicting results might be in part due to the limited sample size and other unmeasured confounding factors. With the development of genomic medicine, the Mendelian randomization (MR) approach somehow provides a possibility to reduce the confounding bias or reverse causation by leveraging genetic variants to instrument a potential exposure and therefore leads to more robust results than the conventional observational approach [17]. Recently, by conducting genome-wide association analyses of own birth weight versus own genetic factors and offspring birth weight versus mother genetic factors, Warrington  et al. have successfully partitioned the genetic effects on birth weight into direct fetal genetic component and indirect maternal genetic component [18]. Consequently, by using the maternal and fetal genetic effects separately, Moen et al. suggested that the maternal intrauterine environment, as proxied by maternal genetic variants that influence offspring birthweight, was unlikely to be a major determinant of adverse cardiometabolic outcomes, but offspring birth weight determined by own genetic factors was found to be associated with offspring cardiometabolic risk factors [19].

Therefore, the aim of this study is to investigate the effect of birth weight on adult BMD by integrating the observational data and both fetal and maternal genetic evidence. We first performed multivariable regression analyses to assess the association of birth weight with BMD-related parameters measured by dual-energy X-ray absorptiometry (DXA) and by quantitative ultrasound (QUS) in the UK Biobank. We also applied the MR approach to test whether the observational relationship between birth weight and adult BMD could be reflected by either indirect maternal or direct fetal genetic effects or both.

## Methods

### Data sources and phenotypes

**Fig. 1:**
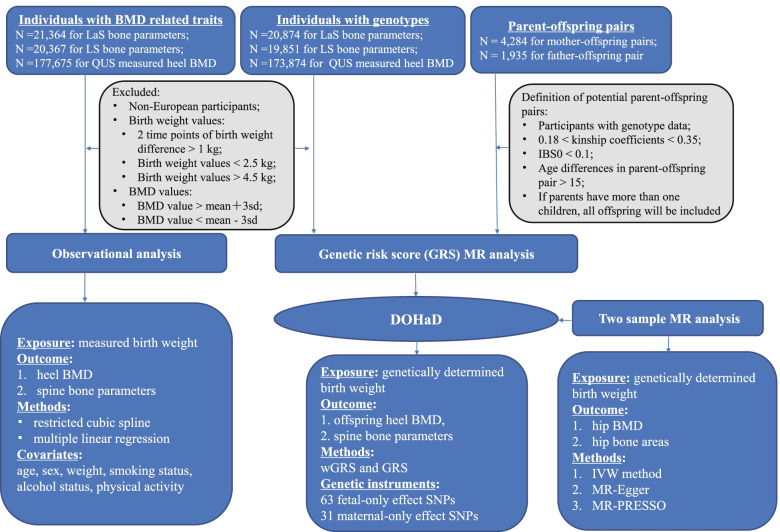
The study design overview. BMD, bone mineral density; IVW, inverse variance-weighted method; MR, Mendelian randomization; MR-PRESSO, MR pleiotropy residual sum and outlier test; QUS, quantitative ultrasound; DXA, dual-energy X-ray absorptiometry

An overview of the study design is illustrated in Fig. 1. In the observational study and weighted genetic risk score analysis, we used individual-level data from the UK Biobank (Application 41376) as we used before [6, 20]. In the present study, the exposure feature of interest was birth weight (Field ID 20022), and we obtained 280,255 participants with birth weight measurements in the UK Biobank dataset. Next, we applied the exclusion criterion step by step: (1) 2 time points of birth weight difference > 1 kg (*N* = 84), (2) birth weight < 2.5 kg, (3) birth weight > 4.5 kg (*N* = 42,613), and (4) non-Europeans (*N* = 29,767). We calculated the mean value of the birth weight if there were more than one visit. The outcomes were the spine DXA BMD and ultrasound-derived heel BMD. The spine DXA data in the UK Biobank were provided as lumbar spine (LS) and lateral spine (LaS), the scanning of the lumbar spine (from L1 to L4) requested the lower legs of the participant be placed on a polystyrene block, bringing the hips and knees to 80° flexion. The scanning of the lateral spine (from L4 to T4) requested the participant to lie on their side and acquire the spine in the lateral plane. Here, we extracted the corresponding bone parameters including the LS BMD (Field ID 23204), LS bone mineral content (BMC) (Field ID 23203), LS bone area (BA) (Field ID 23200), LaS BMD (Field ID 23234), LaS BMC (Field ID 23312), and LaS BA (Field ID 23311). Finally, we got 20,367 participants with both LS data and birth weight measurements, and 21,364 participants with both LaS data and birth weight measurements. Furthermore, we calculated the area-adjusted bone mineral content (BMCadjArea) as an additional outcome; to generate the measurement of BMCadjArea, we first performed a linear regression for BMC with the independent variable bone area, and the residual of BMC for each individual was predicted, then, the BMCadjArea of each individual was calculated as the sum of the mean of BMC and the residual. The heel BMD (Field ID 3084, 3148, and 4105) was estimated from the quantitative ultrasound (QUS) measurement with the following formula: heel estimated BMD = 0.0025926 × (bone ultrasound attenuation + speed of sound) − 3.687. We excluded the unusually large or small heel BMD value (the heel BMD value > mean + 3SD or mean − 3SD), and we also excluded the individuals which were measured by DXA scan, leaving 177,675 individuals with both QUS heel BMD and birth weight measurements.

We extracted the potential confounding factors from the questionnaires such as sex (Field ID 31), age (Field ID 21003), weight (Field ID 21002), smoking status (Field ID 20116), alcohol intake frequency (Field ID 1558), physical activity (Field ID 884, 894, 904, 914), and assessment center (Field ID 54). The physical activity was assessed whether the individual accorded with at least one of the 4 criteria: (1) at least moderate physical activity 5 days a week, (2) vigorous activity once a week, (3) more than 150 min of moderate activity per week, and (4) more than 75 min of vigorous activity per week.

### The selection of instrumental variables

The fetal single nucleotide polymorphisms (SNPs) associated with own birth weight (sample size: 321,223) and the maternal SNPs associated with offspring birth weight (sample size: 230,069 mothers) were extracted from the large-scale GWAS meta-analyses from the Early Growth Genetics (EGG) Consortium and the UK Biobank dataset [18]. Specifically, 205 autosomal SNPs were independently (*r*^2^ < 0.1) associated with birth weight at genome-wide significance *P* < 6.6 × 10^−9^. Of which, 63 autosomal SNPs that have a fetal-only effect on own birth weight represent the effect of own genotype on own birth weight after adjusting the maternal effect on birth weight using the weighted linear regression (WLM method; an approximation of the structural equation model), and 31 autosomal SNPs that had a maternal-only effect on offspring birth weight represent the effect of maternal genotype on offspring birth weight after adjusting the fetal effect on birth weight; 41 autosomal SNPs have both fetal and maternal effects (Additional file 1: Table S1). Here, we selected 63 autosomal SNPs as the fetal instrument variables to investigate the causal association between the genetically determined birth weight and BMD. As a complement, MR analyses with 104 (= 63 + 41) and 205 SNPs were also performed. Similarly, we selected 31 maternal-only effect SNPs as the maternal instrumental variables to represent the intrauterine environment in the parent-offspring pair MR analysis. We also performed MR analyses with 72 (= 31 + 41) and 205 SNPs as additional evidence. Furthermore, we evaluated the strength of the abovementioned instrument variables using two parameters: the proportion of variance explained (*R*^2^) [21], which was calculated from the formula ∑*β*^2^ × 2 × MAF × (1 − MAF), where the *β* was the estimated effect, MAF was the minor allele frequency, and the *F* statistic, which was calculated from the formula *F* = [(*N* – *K* − 1)/*K*] × *R*^2^/(1 − *R*^2^), where the *N* was the sample size, *K* was the number of included SNPs, and the *R*^2^ was the proportion of variance explained by the genetic variants [21]. Typically, the SNPs with an *F*-statistic > 10 were considered as the strong and validated instrumental variables [22]. Additionally, the GWAS summary-level data for femoral neck BMD (FN BMD, *n* = 49,988) [23] was extracted from the GEnetic Factors for Osteoporosis (GEFOS) Consortium (http://www.gefos.org/). We also obtained the GWAS summary-level data for bone areas including the femoral neck area, intertrochanteric area, trochanter area, and total hip area [24] from the deCODE genetics (https://www.decode.com/summarydata). The abovementioned datasets were shown in Additional file 1: Table S2.

### Observational analyses

Firstly, we attempted to test the potential non-linear relationship between the birth weight and each of the bone parameters (BMC, bone area, BMCadjArea, and BMD) using the multivariable restricted cubic spline (RCS) model with four knots (using the “ols” function from the “rms” library in R) adjusting for age, gender, weight, alcohol status, smoking status, and physical status. Then, we performed a multivariable linear regression to explore the association of birth weight with the spine DXA scan results including the lumbar spine and the lateral spine sites in 20,367 and 21,364 participants, respectively; here, the outcomes were LS/LaS BMC, LS/LaS BA, LS/LaS BMCadjArea, and LS/LaS BMD. The confounding factors were sex, age, weight, smoking, alcohol, and physical activity. The baseline characteristics of the DXA measured datasets were shown in Additional file 1: Table S3. Besides, we also implemented the multivariable linear regressions with the same confounders to explore the relationship between birth weight and QUS measured heel BMD in 177,675 individuals. Of note, these individuals did not overlap with the DXA-measured participants, which could be served as an independent replication. The baseline characteristics of the QUS-measured heel BMD dataset were shown in Additional file 1: Table S4. To be noted, we applied a standardized approach (*z*-scores) for the outcomes to give a mean = 0 and standard deviation = 1 and then conducted the above analyses. All observational analyses were performed using the R software (https://www.r-project.org/), and *P* < 0.05 was set as the significant threshold.

### Mendelian randomization

#### Fetal SNPs and own birth weight

We performed a weighted genetic risk score (wGRS) analysis in the UK Biobank using the individual-level data to test the relationship between genetically determined birth weight and BMC, bone area, BMCadjArea, and BMD. Here, we constructed the wGRS with the selected instrumental variables (63, 104, 205 SNPs described in “The selection of instrumental variables” section) [18] using the PLINK software (http://zzz.bwh.harvard.edu/plink/) in the UK Biobank individuals; briefly, the wGRS was calculated by the summation of the individual’s effect alleles, weighted by corresponding effect sizes [25], and we used the option *--score sum* to obtain the sum of valid per-allele scores [6]. Then, we applied the multivariable linear regression adjusting for sex, age, weight, smoking, alcohol, and physical activity to assess the genetically determined birth weight on BMD-related parameters (LS/LaS BMC, LS/LaS BA, LS/LaS BMCadjArea, and LS/LaS BMD) in 19,851 participants with LS BMD measurement, 20,874 participants with LaS BMD measurement, and 173,874 participants with heel BMD measurement. In addition, we implemented a two-sample MR analysis to detect the association of genetically determined birth weight with hip bone areas and femoral neck BMD. The information on the instrumental variables was shown in Additional file 1: Table S5-S7. The inverse variance weighted (IVW), MR-Egger, MR-Egger intercept, and MR-PRESSO methods were used for the two-sample MR analyses [26–28]. All MR analyses were conducted using the “TwoSampleMR” and “MR-PRESSO” packages in R [27, 29]. In this analysis, *P* < 0.05 was considered as the significant level. Additionally, we also calculated the statistical power for MR analyses using the “online sample size and power calculator for Mendelian randomization” (https://sb452.shinyapps.io/power/), with the parameters of the sample size, the proportion of variance explained (*R*^2^), the causal effect, and the significance level (0.05).

#### Genetic pleiotropy assessment

We conducted a further sensitivity analysis to address the horizontal pleiotropy assumption of the MR analysis. According to the existing data in the UK Biobank, we selected the bone metabolic markers including alkaline phosphatase (Field ID 30610), vitamin D (Field ID 30890), oestradiol (Field ID 30800), calcium (Field ID 30680), and inflammatory markers including C-reactive protein (Field ID 30710), rheumatoid factor (Field ID 30820), and urea (Field ID 30670) as the confounding risk factors. We performed a linear regression to test the associations between the genetically determined birth weight (wGRS) and the potential risk factors of bone parameters in a one-sample MR analysis. Additionally, we also evaluated the pleiotropic association of instrumental variables (SNPs associated with birth weight) with potential confounders (*P* < 5 × 10^−8^). Previous studies have suggested some risk factors for bone mineral density, including anorexia nervosa [30], alcohol [31, 32], body mass index (BMI) [33], Crohn’s disease [34], education years [35], inflammatory bowel disease [36, 37], rheumatoid arthritis [38], smoking [39], ulcerative colitis [36, 37], and 25-hydroxyvitamin D [40]. We checked the association of the SNPs in the GWAS catalog (https://www.ebi.ac.uk/gwas/) to exclude any of the pleiotropic instrumental variables (Additional file 1: Table S8) [41–49].

#### Maternal SNPs and offspring birth weight

With the UK Biobank individual-level dataset, we also examined whether the intrauterine environment (proxied by the maternal SNPs that influence offspring birth weight) was associated with the offspring adult BMD. Firstly, we confirmed the parent-offspring pairs in the UK Biobank with the released kinship information file “ukbA_rel_sP.txt” (https://biobank.ndph.ox.ac.uk/ukb/ukb/docs/ukbgeneinstruct.html). The kinship information file contained kinship coefficients and estimates of the proportion of SNPs with zero identical-by-state (IBS0). The parent-offspring pairs were defined using the formula $\frac{1}{2^{5/2}}<\mathrm{kinship}\ \mathrm{coefficient}<\frac{1}{2^{3/2}}$ and IBS0 < 0.1 as recommended by Manichaikul et al. [50]. We then excluded the parent-offspring pairs who had less than 15 years of age difference between the mother’s/father’s age and the son’s/daughter’s age. We kept all the offsprings when one mother/father had one or more children. Finally, we obtained 6219 parent-offspring pairs of European ancestry, that is, 4284 mother-offspring pairs and 1935 father-offspring pairs. The detailed process of quality control was provided in Fig. 1. Eventually, we calculated the unweighted genetic risk scores (GRS) with 31 maternal-only effect SNPs [18] for the mothers/fathers in the parent-offspring pairs. Then, we examined the association of the maternal SNP-determined birth weight (exposure) and offspring heel BMD (outcome), either controlling for the offspring GRS or not, in the linear mixed-effects model (with a random effects term to account for within sibling variance) adjusting for the offspring age, sex, weight, and assessment centers in the analysis. Of note, some parent-offspring pairs might miss the covariates data. We also performed MR analyses with 72 and 205 SNPs (see the “The selection of instrumental variables” section) as additional evidence. All statistical analyses were performed with the “lme4” package in R.

## Results

### The association of birth weight with BMC, bone area, BMCadjArea, and BMD

The results from the restricted cubic spline analysis demonstrated that there was no evidence of a non-linear relationship between birth weight and BMD-related parameters (BMC, bone area, BMCadjArea, and BMD) (*P* > 0.05, Additional file 1: Fig. S1-S3). In the multivariable linear regression analyses at the site of the lumbar spine (LS), we found that higher birth weight was associated with higher adult LS BMC (*β*-coefficient = 0.06, *P* = 2.87 × 10^−7^) and higher LS area (*β*-coefficient = 0.17, *P* < 2.00 × 10^−16^) (Table 1). However, when BMC was adjusted for the bone area (BMCadjArea), the direction of association between birth weight and BMCadjArea turned negative with significance (*β*-coefficient = − 0.01, *P* = 2.27 × 10^−14^) (Table 1). Similarly, if we looked at the BMD directly, we found a higher birth weight was associated with lower adult LS BMD (*β*-coefficient = − 0.04, *P* = 0.007) (Table 1). At the site of the lateral spine (LaS), the association between birth weight and LaS BMC was not observed (*P* = 0.83); however, when the area was adjusted, we found a strong negative association between birth weight and LaS BMCadjArea (*β*-coefficient = − 0.05, *P* = 0.001) (Table 1). In addition, we found that higher birth weight was associated with lower adult LaS BMD (*β*-coefficient = − 0.03, *P* = 0.02) (Table 1). At the site of the heel, the higher birth weight was associated with QUS-estimated BMD (*β*-coefficient = − 0.06, *P* < 2.00 × 10^−16^), and the direction of the effect was consistent with the findings from DXA-measured spine BMD (Table 1).

**Table 1:** The analyses between birth weight and BMC, bone area, BMCadjArea, and BMD in UK Biobank dataset

Measurement	Site	Outcome	Effect	Se	*P*
DXA	Lumbar spine (LS, *N* = 20,367)	BMC	0.06	0.01	2.87 × 10^−7^
		Bone area	0.17	0.01	< 2.00 × 10^−16^
		BMD	− 0.04	0.01	0.007
		BMCadjArea	− 0.01	0.01	2.27 × 10^−14^
	Lateral spine (LaS, *N* = 21,364)	BMC	− 0.002	0.01	0.83
		Bone area	0.02	0.01	0.04
		BMD	− 0.03	0.01	0.02
		BMCadjArea	− 0.05	0.01	0.001
QUS	Heel (*N*=177,675)	BMD	− 0.06	0.01	< 2.00 × 10^−16^

BMCadjArea: the linear regression for BMC with independent variable bone area, and the residual of BMC for each individual was predicted, then, the BMCadjArea of each individual was calculated as the sum of the mean of BMC and the residual

Exposure: measured birth weight

Outcome: BMC, bone area, BMCadjArea, and BMD. The measurements of the outcomes were standardized (*z*-scores) to give a mean = 0 and standard deviation = 1 to ease comparison

All analyses were adjusted for sex, age, weight, smoking, alcohol, and physical activity

### Fetal SNP-determined birth weight, BMC, bone area, BMCadjArea, and BMD

A total of 63 fetal-only effect SNPs explained 1.7% of the variance (*R*^2^) of the own birth weight, and the corresponding *F*-statistic was 88. We calculated the weighted genetic risk score (wGRS) for each individual with these 63 fetal-only effect SNPs within the UK Biobank dataset, and the weighted effect size (beta value) of each SNP on birth weight was extracted from Warrington et al. [18]. We conducted a multivariable linear regression analysis to test the relationship between fetal-only effect SNP-determined birth weight and spine DXA scan parameters and QUS-estimated heel BMD. We observed that higher genetically determined birth weight was significantly associated with higher LS area (*β*-coefficient = 0.15, *P* = 1.26 × 10^−6^) but associated with lower LS BMCadjArea (*β*-coefficient = − 0.19, *P* = 6.03 × 10^−5^) and lower LS BMD (*β*-coefficient = − 0.10, *P* = 0.01) (Table 2). At the site of the lateral spine (LaS), we also found that higher fetal SNP-determined birth weight was associated with lower LaS BMCadjArea (*β*-coefficient = − 0.17, *P* = 0.0001) and lower LaS BMD (*β*-coefficient = − 0.12, *P* = 0.0003) (Table 2). Similarly, the higher fetal-only effect SNP-determined birth weight was found to be associated with lower QUS-estimated BMD at the site of the heel (*β*-coefficient = − 0.11, *P* = 3.33 × 10^−13^) (Table 2). Additionally, we also performed the multivariable linear regression analysis of the wGRS calculated with 104 and 205 SNPs (see the “Methods” section) and obtained similar trends of associations (Table 2). The 104 and 205 SNPs explained 2.4% and 3.6% of the variance (*R*^2^) of the own birth weight, respectively, and the corresponding *F*-statistics were 58 and 75. Furthermore, we conducted a sensitivity analysis to address the horizontal pleiotropy assumption of the MR analysis. Within the selected confounding risk factors such as the bone metabolic markers (alkaline phosphatase, vitamin D, oestradiol, calcium) and inflammatory markers (C-reactive protein, rheumatoid factor, urea), we only found a significant relationship between the fetal-only effect SNP-determined birth weight (wGRS) and serum calcium (Additional file 1: Table S9). Then, we included serum calcium in the one-sample MR model (adjusting the covariates sex, age, weight, smoking, alcohol, physical activity, and serum calcium), and we obtained similar results as before (Additional file 1: Table S10). Therefore, by leveraging the genetic instrumental variables with fetal-SNPs, the wGRS analysis provided additional evidence to support the observational findings, suggesting that the observational findings that higher birth weight was associated with lower BMD might not be biased by potential confounding factors or reverse causality.

**Table 2:** The association between fetal SNP-determined birth weight and adult BMC, bone area, BMCadjArea, and BMD in UK Biobank dataset

Measurement	Sites	Outcome	63 autosomal SNPs only with fetal effect			104 autosomal SNPs			205 autosomal SNPs		
			Effect	SE	*P*	Effect	SE	*P*	Effect	SE	*P*
DXA	Lumbar spine (LS, *N* = 19,851)	BMC	0.01	0.03	0.72	0.01	0.02	0.86	0.04	0.02	0.05
		Bone area	0.15	0.03	1.26 × 10^−6^	0.14	0.02	1.20 × 10^−8^	0.17	0.02	< 2.00 × 10^−16^
		BMD	− 0.10	0.04	0.01	− 0.11	0.03	0.002	− 0.07	0.03	0.01
		BMCadjArea	− 0.19	0.05	6.03 × 10^−5^	− 0.20	0.04	4.80 × 10^−7^	− 0.16	0.03	1.04 × 10^−7^
	Lateral spine (LaS, *N* = 20,874)	BMC	− 0.07	0.03	0.03	− 0.05	0.02	0.04	− 0.02	0.02	0.19
		Bone area	0.01	0.03	0.64	0.04	0.03	0.18	0.05	0.02	0.05
		BMD	− 0.12	0.03	0.0003	− 0.11	0.03	7.54 × 10^−5^	− 0.07	0.02	9.00 × 10^−4^
		BMCadjArea	− 0.17	0.04	0.0001	− 0.18	0.03	5.81 × 10^−7^	− 0.14	0.02	1.86 × 10^−6^
QUS	Heel (*N* = 173,874)	Heel BMD	− 0.11	0.02	3.33 × 10^−13^	− 0.11	0.01	< 2.00 × 10^−16^	− 0.10	0.01	< 2.00 × 10^−16^

Exposure: weighted genetic risk scores, calculated from the 63 fetal-only effect SNPs, 104 autosomal SNPs (63 SNPs + 41 SNPs with both fetal and maternal effect), and 205 autosomal SNPs (total significant SNPs)

Outcome: BMC, bone area, BMCadjArea, and BMD. The measurements of the outcomes were standardized (*z*-scores) to give a mean = 0 and standard deviation = 1 to ease comparison

All analyses were adjusted for sex, age, weight, smoking, alcohol, and physical activity

**Fig. 2:**
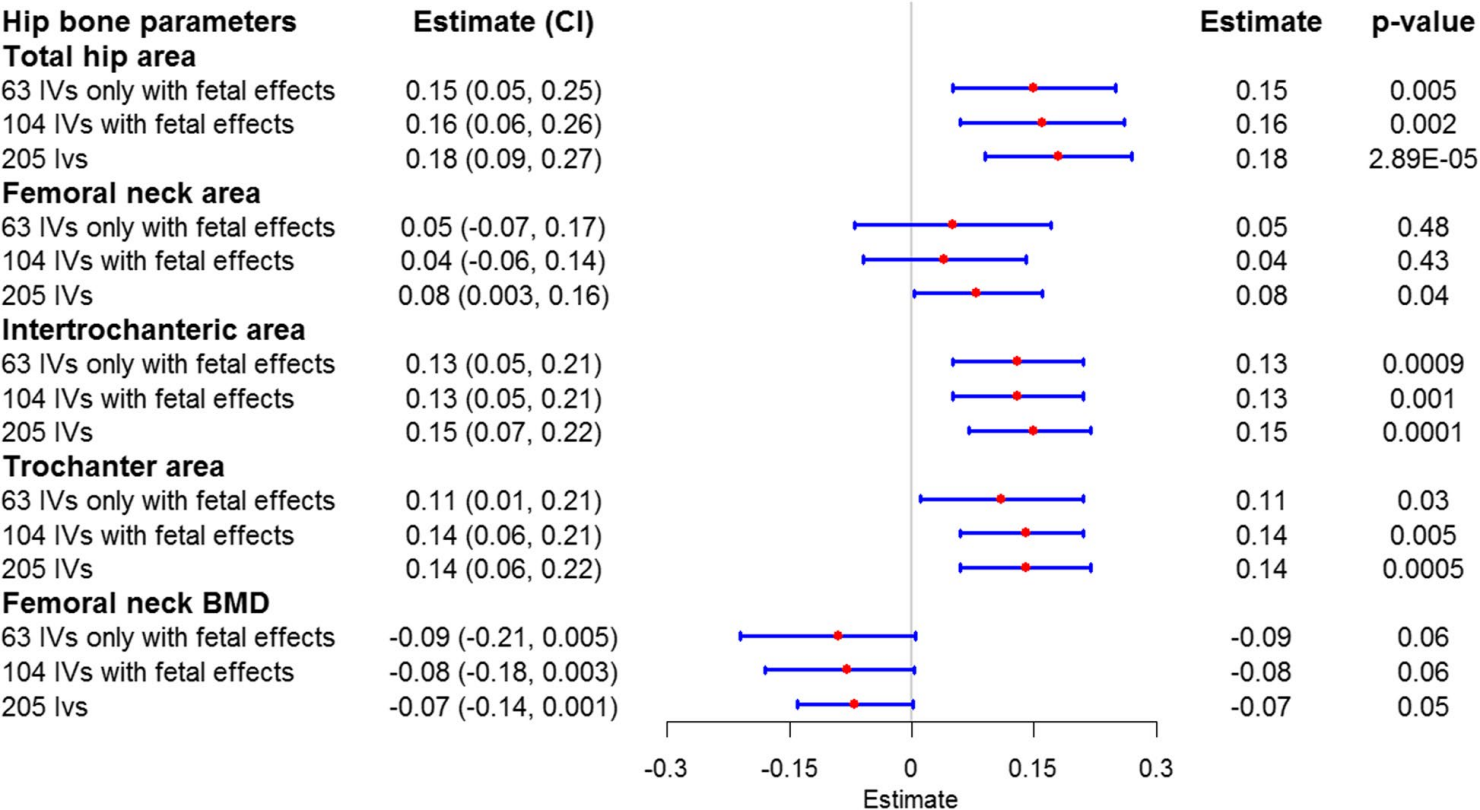
The causal relationship between the birth weight determined by fetal genetic effect and the hip bone parameters in MR-PRESSO analysis

Because bone area data at the hip cannot be downloaded from the UK Biobank, we tested the association of 63 fetal-only effect SNP-determined birth weight with bone area and BMD at the hip using a two-sample MR analysis. We found that the 63 fetal-only effect SNP-determined higher birth weight was associated with the higher total hip area (*β*-coefficient = 0.15, *P* = 0.005), the intertrochanteric area (*β*-coefficient = 0.13, *P* = 0.0009), and the trochanter area (*β*-coefficient = 0.11, *P* = 0.03) in two-sample MR-PRESSO analysis (Fig. 2 and Additional file 1: Table S11). Additionally, a higher 63 fetal-only effect SNP-determined birth weight was associated with lower femoral neck BMD at a nominal significance (*β*-coefficient = − 0.09, *P* = 0.06). Furthermore, we also performed a two-sample MR analysis with 104 and 205 SNPs (see the “Methods” section) as the instrumental variables and obtained similar trends of association (Fig. 2). The MR-Egger intercept also showed no evidence of directional horizontal pleiotropy for the above three types of fetal instruments (MR-Egger intercept; *P* > 0.05), and the statistical power was 94–100% as evaluated by the online tool (Additional file 1: Table S11). Additionally, we evaluated the pleiotropic association of instrumental variables (SNPs associated with birth weight) with potential confounders (such as anorexia nervosa, alcohol, BMI, Crohn’s disease, education year, inflammatory bowel disease, rheumatoid arthritis, smoking, ulcerative colitis, and 25-hydroxyvitamin D). We found that rs1547669, rs6911024, and rs9267812 were associated with rheumatoid arthritis, rs516246 was associated with alcohol and Crohn’s disease, and rs7903146 was associated with BMI. Likewise, excluding these SNPs did not significantly change the results of the MR analysis (Additional file 1: Table S12).

### Maternal SNP-determined birth weight and BMD

In order to test whether maternal-only effect SNP-determined offspring birth weight was associated with the offspring BMD, we calculated the unweighted genetic risk scores (GRS) with 31 maternal-only effect SNPs in 4284 mother-offspring pairs and 1935 father-offspring pairs. We first checked whether the maternal GRS was associated with offspring birth weight and found that the maternal GRS was indeed strongly associated with increased offspring birth weight before (*β*-coefficient = 0.01, *P* = 5.07 × 10^−7^) and after conditioning on offspring GRS (*β*-coefficient = 0.01, *P* = 0.0003) in 4284 mother-offspring pairs (Table 3). In the linear mixed model (exposure: maternal GRS, outcome: offspring heel BMD) adjusting for age, sex, weight, and assessment centers, we found that higher maternal-only effect SNP-determined offspring birth weight was associated with lower adult BMD in both before (*β*-coefficient = − 0.001, *P* = 0.03) and after (*β*-coefficient = − 0.001, *P* = 0.04) adjusting for offspring GRS in 4284 mother-offspring pairs (Table 3). Additionally, we also performed a linear mixed model analysis of the GRS calculated with 72 and 205 SNPs (see the “Methods” section) and obtained similar trends of association (Table 3). Here, 31, 72, and 205 SNPs explained 1.0%, 1.8%, and 3.0% of the variance (*R*^2^) of the offspring birth weight, respectively, and the corresponding *F*-statistics were 74, 58, and 34. We did not detect any significant relationship in 1935 father-offspring pairs with these types of maternal SNPs (all *P* > 0.05) (Table 3).

**Table 3:** The association between maternal SNP-determined birth weight and offspring BMD in the UK Biobank dataset

Analysis sample	Outcome	Exposure	31 autosomal SNPs only with maternal effect			72 autosomal SNPs			205 autosomal SNPs		
			Effect	SE	*P*	Effect	SE	*P*	Effect	SE	*P*
Mother-offspring pairs (*N* = 4,284)	Offspring heel BMD	Maternal GRS	− 0.001	0.0006	0.03	− 0.001	0.0003	0.01	− 0.001	0.0002	0.002
		Maternal GRS—adjusted for offspring GRS	− 0.001	0.0007	0.04	− 0.001	0.0004	0.01	− 0.001	0.0002	0.009
	Offspring birth weight	Maternal GRS	0.01	0.003	5.07×10^−7^	0.01	0.001	1.40×10^−9^	0.007	0.001	8.76×10^−12^
		Maternal GRS—adjusted for offspring GRS	0.01	0.003	0.0003	0.007	0.002	0.0002	0.003	0.001	0.008
Father-offspring pairs (*N* = 1,935)	Offspring heel BMD	Paternal GRS	0.0003	0.001	0.76	0.0003	0.0006	0.63	− 0.0003	0.0003	0.41
		Paternal GRS—adjusted for offspring GRS	− 0.000002	0.001	0.98	0.00001	0.0007	0.99	− 0.0003	0.0004	0.36
	Offspring birth weight	Paternal GRS	− 0.001	0.004	0.76	0.001	0.002	0.64	0.002	0.001	0.19
		Paternal GRS—adjusted for offspring GRS	− 0.008	0.005	0.09	− 0.005	0.003	0.08	− 0.002	0.002	0.20

The regression coefficients give the estimated expected change in offspring eBMD (g/cm^2^), per one unit (i.e., allele) increase in maternal/paternal genetic risk score (calculated from the 31 maternal-only effect SNPs, 72 autosomal SNPs with both fetal and maternal effect and 205 total significant autosomal SNPs), with or without conditioning on offspring genetic risk score

The linear mixed-effects model was applied. All analyses were adjusted for offspring age, sex, weight, and assessment center

## Discussion

In this study, the observational analyses suggested that higher birth weight was associated with increased adult spine bone area but decreased spine BMCadjArea, spine BMD, and heel BMD. By leveraging fetal-only effect SNPs as the genetic instrumental variables, the birth weight determined by own genetic factors was found to be positively associated with offspring bone area but negatively associated with BMD. Furthermore, by using the parent-offspring pairs, our results suggested that the maternal intrauterine environment, as proxied by maternal genetic variants that influence offspring birthweight, was also a determinant of adult BMD. In other words, the observational relationship between birth weight and BMD could be reflected by both the direct fetal and indirect maternal genetic effects.

Lower birth weight often occurs along with inferior intrauterine environments, for example, malnutrition or stress during pregnancy. The Developmental Origin of Health and Disease (DOHaD) hypothesis proposes that exposure to a hostile uterine environment would change the fetus’ structure, function, and metabolism in ways that lead to significantly adverse consequences on an individual’s short- and long-term health, followed by the reduced fetal growth rate, low body weight at birth, and the high risk of future related diseases [51, 52]. The hypothesis was applied well in the studies of cardiovascular diseases [9, 53]. Other epidemiologic observations also showed that lower birth weight was associated with an increased risk of stroke, type 2 diabetes mellitus (T2DM), obesity, and hypertension [54–57].

A previous study has tried to investigate the relationship between birth weight and osteoporosis risk, but as they listed as a limitation, they failed to analyze the DXA data, which is the gold standard for the diagnosis of osteoporosis [58]. In our study design, the observation that higher birth weight was associated with lower adult BMD was consistently shown in two independent study samples: the DXA scan data (20,367 and 21,364 participants) and QUS measurement data (177,675 samples). With DXA scan data, we could also look at the association between birth weight and BMC/bone area. It is better to pay close attention to BMC/bone area because Dennison et al. demonstrated that birth weight had greater contributions to bone size and mineral content than to bone mineral density [10], and birth weight was previously detected to be associated with the forearm and tibial bone size in the old population aged 65–73 years [59]. In our study, we found that higher birth weight was associated with higher adult LS BMC and higher LS area. Interestingly, the effect of birth weight on bone size was much larger than the effect on BMC after standardization. At the site of the lateral spine (LaS), the association between birth weight and LaS BMC was not observed, but birth weight was positively associated with the LaS area. These results suggested that higher birth weight would lead to lower adult BMD; this might, to some extent, be because of the larger effect of birth weight on bone size than on BMC. Coincidentally, Steer et al. reported similar results in teenagers that birth weight was positively related to bone size but inversely related to cortical BMD, but they suggested that the relationship was mediated by the effects on late onset of puberty and bone resorption [14]. In addition, we included an alternative parameter BMCadjArea (BMC adjusted for bone area) in our analysis, and as expected, the results for BMCadjArea were consistent with the result for BMD, because BMCadjArea is conceptually similar to BMD.

Mendelian randomization (MR) is an approach to test the causality of an observed relationship between an exposure and an outcome [17]. Recently, some studies have tried to leverage the MR method to detect the relationship between birth weight and non-communicable diseases (i.e., atrial fibrillation, type 2 diabetes, and cardiovascular diseases) to address the DOHaD hypothesis [60–62]. However, these MR analyses only took the offspring genetic variants to instrument the birth weight. An optimal way is to also take the maternal genotypes that effect the offspring’s birth weight to proxy the intrauterine environment to test aspects of DOHaD [63]. Benefiting from the study by Warrington  et al., the genetic effects on birth weight were partitioned into direct fetal genetic component and indirect maternal genetic component [18]. They identified a total of 205 autosomal SNPs associated with birth weight. Of which, 63 autosomal SNPs that have a fetal-only effect on own birth weight represent the effect of own genotype on own birth weight after adjusting the maternal effect on birth weight, 31 autosomal SNPs that had a maternal-only effect on offspring birth weight represent the intrauterine environment, and 41 autosomal SNPs have both fetal and maternal effects [18]. By leveraging these SNPs with different effects, we found that higher fetal-only effect SNP-determined birth weight was associated with lower LS/FN BMD and heel BMD. We also found that higher maternal-only effect SNP-determined offspring birth weight was associated with lower adult BMD after adjusting for offspring genetic effects but did not find any association evidence in father-offspring pairs, which is suggestive of DOHaD mechanisms (related to lower birthweight) through the effect on bone size. Here, we calculated the unweighted GRS in mothers because we were ignorant of the effect size of each allele to avoid the inflation in type 1 error [19]. Previously, Moen et al. suggested that the maternal intrauterine environment, as proxied by maternal genetic variants that influence offspring birth weight, was unlikely to be a major determinant of adverse cardiometabolic outcomes, but offspring birth weight determined by own genetic factors was found to be associated with offspring cardiometabolic risk factors [19]. However, in our study, we suggested that both indirect maternal and direct fetal genetic effects reflected the observational relationship between higher birth weight and lower adult bone mass.

Acknowledged as a limitation of this study, we excluded the individuals with extreme birth weight (very low birth weight < 1.5 kg, low birth weight > 1.5 kg and < 2.5 kg, and birth weight > 4.5 kg) in our study to exclude likely pre-term births and fetal macrosomia. Hovi et al. found that adults born with very low birth weight (< 1.5 kg) had lower LS BMD compared with the participants born at normal birth weight at ages 18.5 to 27.1 [64]. When we checked back in the UK Biobank dataset, we found that the participants born with very low birth weight had lower lumbar spine BMD than the participants born with normal birth weight (1.166 vs 1.179 g/cm^2^). Therefore, the results should be interpreted with caution when facing the extreme value of birth weight.

## Conclusions

In summary, we observed that higher birth weight would lead to lower bone mass in later adulthood; this might be because of the larger effect of birth weight on bone size than on BMC. By leveraging the genetic instrumental variables with maternal- and fetal-specific SNP information, we detected the fetal genetic contributions to birth weight had a casually positive effect on the bone area but a negative effect on BMD. Furthermore, the maternal intrauterine environment, as proxied by maternal genetic variants that influence offspring birth weight, was also a determinant of adult BMD.

## Supplementary Information

**Additional file 1: Fig. S1.** Restricted cubic spline analysis to show the relationship between birth weight and Lumbar spine bone parameters. **Fig. S2.** Restricted cubic spline analysis to show the relationship between birth weight and Lateral spine bone parameters. **Fig. S3.** Restricted cubic spline analysis to show the relationship between birth weight and heel BMD. **Table S1.** Characteristics of the single nucleotide polymorphisms associated with the birth weight. **Table S2.** Detailed information on the GWAS summary-statistic data for birth weight and bone parameters. **Table S3.** Characteristics of participants from the UK Biobank dataset in the observational analysis for the Spine DXA data. **Table S4.** Characteristics of participants from the UK Biobank dataset in the model 3 observational analysis for heel BMD measured by QUS. **Table S5.** Summary statistics for the 63 SNPs used in instrumental variable analysis to assess the effect of fetal birth weight on the hip bone parameters. **Table S6.** Summary statistics for the 104 SNPs used in instrumental variable analysis to assess the effect of fetal birth weight on the hip bone parameters. **Table S7.** Summary statistics for the 205 SNPs used in instrumental variable analysis to assess the effect of fetal birth weight on the hip bone parameters. **Table S8.** P values for the associations of 205 birth weight variants with potential risk factors. **Table S9.** The association between fetal SNPs determined birth weight and potential risk factors in UK biobank dataset. **Table S10.** The association between fetal SNPs determined birth weight and adult BMC, Bone area, BMCadjArea and BMD in UK biobank dataset (confounders including calcium level). **Table S11.** Causal associations of the fetal genotype effects on birth weight (63, 104, 205 instrument variables) with hip bone parameters in two-sample MR analyses. **Table S12.** Causal associations of the fetal genotype effects on birth weight (62, 102, 200 instrumental variables which excluded the potential pleiotropic variants) with hip bone parameters in two-sample MR analyses.

## Data Availability

The genetic summary statistics for BMD and bone size were downloaded from the GEnetic Factors for OSteoporosis (GEFOS) consortium (http://www.gefos.org/) and deCODE Genetics (https://www.decode.com/summarydata/). The individual-level genetic and phenotype data require permission from the UK Biobank (Application 41376) (https://www.ukbiobank.ac.uk/).

## Abbreviations

BMC: Bone mineral contents
BMCadjArea: BMC-adjusted bone area
BMD: Bone mineral density
DXA: Dual-energy X-ray absorptiometry
GRS: Genetic risk score
LaS: Lateral spine
LS: Lumbar spine
MR: Mendelian randomization
QUS: Quantitative ultrasound
