# Supplementary material for: Both indirect maternal and direct fetal genetic effects reflect the observational relationship between higher birth weight and lower adult bone mass
Source: BMC Med. 2022 Oct 4;20:361. doi: 10.1186/s12916-022-02531-w (PMC9531399; doi:10.1186/s12916-022-02531-w)
Supplement: Supplementary file 1 — Additional file 1: Fig. S1. Restricted cubic spline analysis to show the relationship between birth weight and Lumbar spine bone parameters. Fig. S2. Restricted cubic spline analysis to show the relationship between birth weight and Lateral spine bone parameters. Fig. S3. Restricted cubic spline analysis to show the relationship between birth weight and heel BMD. Table S1. Characteristics of the single nucleotide polymorphisms associated with the birth weight. Table S2. Detailed information on the GWAS summary-statistic data for birth weight and bone parameters. Table S3. Characteristics of participants from the UK Biobank dataset in the observational analysis for the Spine DXA data. Table S4. Characteristics of participants from the UK Biobank dataset in the model 3 observational analysis for heel BMD measured by QUS. Table S5. Summary statistics for the 63 SNPs used in instrumental variable analysis to assess the effect of fetal birth weight on the hip bone parameters. Table S6. Summary statistics for the 104 SNPs used in instrumental variable analysis to assess the effect of fetal birth weight on the hip bone parameters. Table S7. Summary statistics for the 205 SNPs used in instrumental variable analysis to assess the effect of fetal birth weight on the hip bone parameters. Table S8. P values for the associations of 205 birth weight variants with potential risk factors. Table S9. The association between fetal SNPs determined birth weight and potential risk factors in UK biobank dataset. Table S10. The association between fetal SNPs determined birth weight and adult BMC, Bone area, BMCadjArea and BMD in UK biobank dataset (confounders including calcium level). Table S11. Causal associations of the fetal genotype effects on birth weight (63, 104, 205 instrument variables) with hip bone parameters in two-sample MR analyses. Table S12. Causal associations of the fetal genotype effects on birth weight (62, 102, 200 instrumental variables which excluded the potential p [file 12916_2022_2531_MOESM1_ESM.docx]

Additional file 1

**Fig. S1.** Restricted cubic spline analysis to show the relationship between birth weight and Lumbar spine bone parameters.

**Fig. S2.** Restricted cubic spline analysis to show the relationship between birth weight and Lateral spine bone parameters.

**Fig. S3.** Restricted cubic spline analysis to show the relationship between birth weight and heel BMD.

**Table S1.** Characteristics of the single nucleotide polymorphisms associated with the birth weight.

**Table S2.** Detailed information on the GWAS summary-statistic data for birth weight and bone parameters.

**Table S3.** Characteristics of participants from the UK Biobank dataset in the observational analysis for the Spine DXA data.

**Table S4.** Characteristics of participants from the UK Biobank dataset in the model 3 observational analysis for heel BMD measured by QUS.

**Table S5.** Summary statistics for the 63 SNPs used in instrumental variable analysis to assess the effect of fetal birth weight on the hip bone parameters.

**Table S6.** Summary statistics for the 104 SNPs used in instrumental variable analysis to assess the effect of fetal birth weight on the hip bone parameters.

**Table S7.** Summary statistics for the 205 SNPs used in instrumental variable analysis to assess the effect of fetal birth weight on the hip bone parameters.

**Table S8.** P Values for Associations of 205 birth weight variants with potential risk factors

**Table S9.** The association between fetal SNPs determined birth weight and potential risk factors in UK biobank dataset.

**Table S10.** The association between fetal SNPs determined birth weight and adult BMC, Bone area, BMCadjArea and BMD in UK biobank dataset (confounders including calcium level).

**Table S11.** Causal associations of the fetal genotype effects on birth weight (63, 104, 205 instrument variables) with hip bone parameters in two-sample MR analyses.

**Table S12.** Causal associations of the fetal genotype effects on birth weight (62, 102, 200 instrumental variables which excluded the potential pleiotropic variants) with hip bone parameters in two-sample MR analyses.


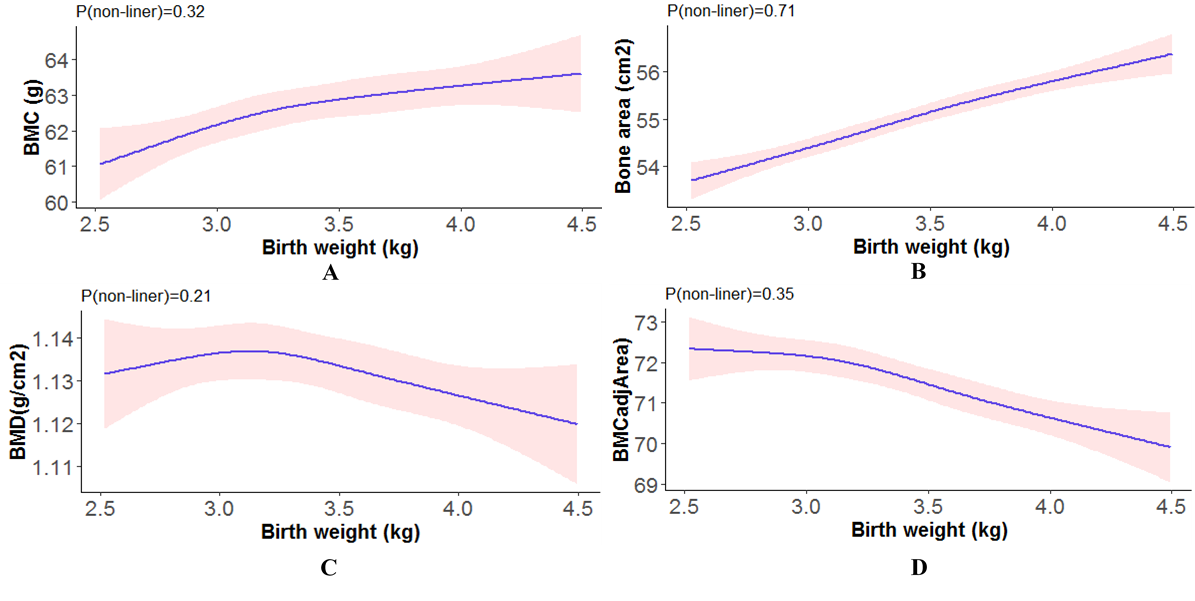


**Fig. S1.** Restricted cubic spline analysis to show the relationship between birth weight and Lumbar spine bone parameters. (A) Relationship between birth weight and bone mineral content (BMC). (B) Relationship between birth weight and bone area. (C) Relationship between birth weight and bone mineral density (BMD). (D) Relationship between birth weight and BMCadjArea.


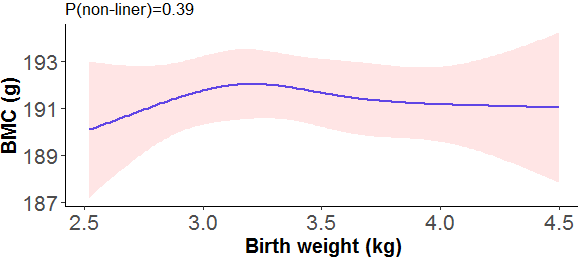

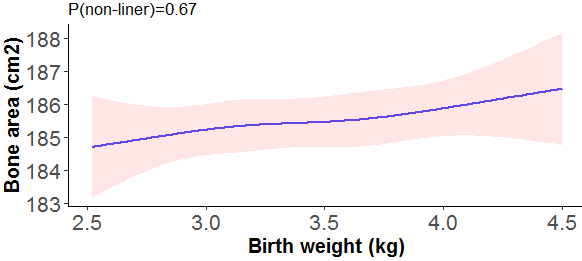

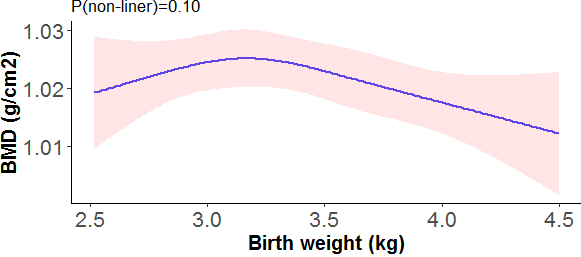

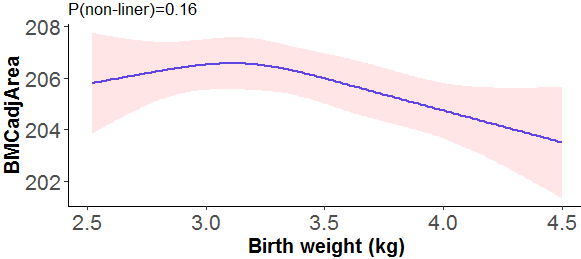


**E**

**F**

**G**

**H**

**Fig. S2.** Restricted cubic spline analysis to show the relationship between birth weight and Lateral spine bone parameters. (E) Relationship between birth weight and bone mineral content (BMC). (F) Relationship between birth weight and bone area. (G) Relationship between birth weight and bone mineral density (BMD). (H) Relationship between birth weight and BMCadjArea.


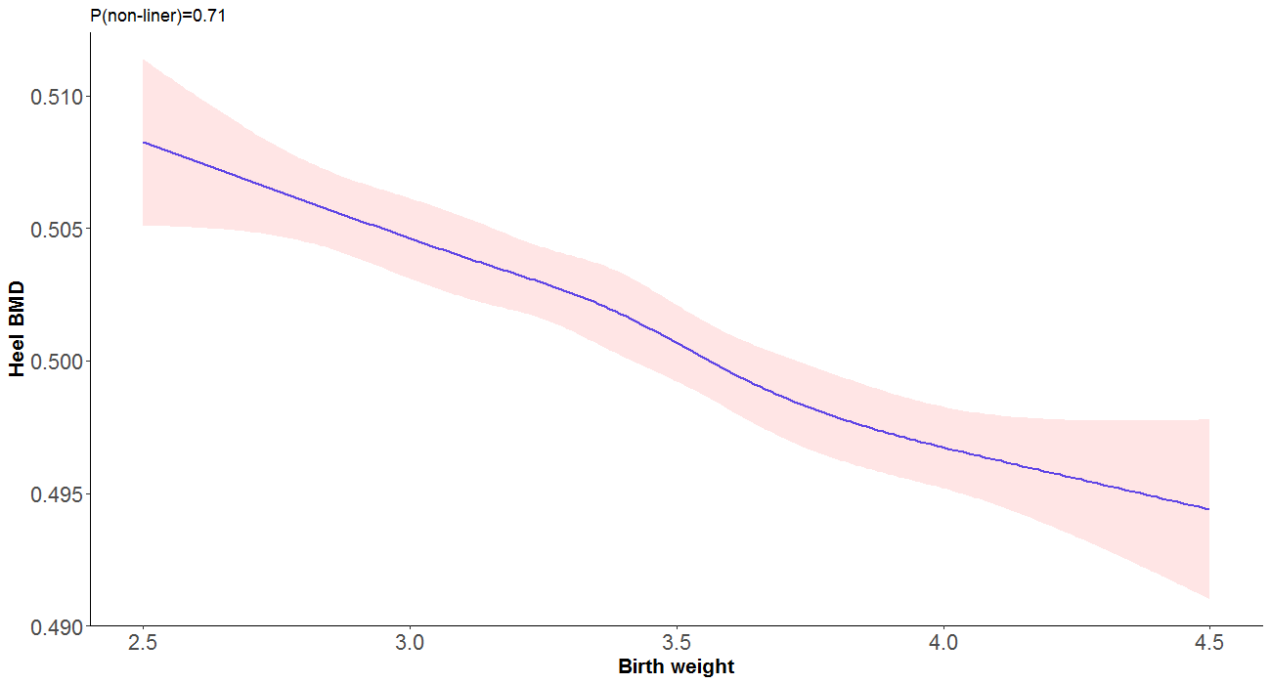


**Fig. S3.** Restricted cubic spline analysis to show the relationship between birth weight and heel BMD.

**Table S1.** Characteristics of the single nucleotide polymorphisms associated with the birth weight.

| **SNP** | **Chromosome** | **Position (hg19)** | **Maternal effect** | **Maternal effect only** | **Fetal effect** | **Fetal effect only** | **Effect Allele^a^** | **Other Allele** |
| --- | --- | --- | --- | --- | --- | --- | --- | --- |
| rs17367504 | 1 | 11862778 | √ | √ |  |  | G | A |
| rs12401656 | 1 | 43456767 |  |  | √ | √ | A | G |
| rs80278614 | 1 | 119412317 |  |  | √ | √ | G | A |
| rs905938 | 1 | 154991389 |  |  |  |  | C | T |
| rs670523 | 1 | 155878732 |  |  |  |  | G | A |
| rs72480273 | 1 | 161644871 |  |  |  |  | C | A |
| rs10913200 | 1 | 176521655 | √ |  | √ |  | G | A |
| rs61830764 | 1 | 212289976 |  |  | √ | √ | G | A |
| rs3806315 | 1 | 214724668 |  |  |  |  | A | G |
| rs708122 | 1 | 228216997 |  |  |  |  | C | A |
| rs10495563 | 2 | 9662210 | √ |  | √ |  | A | G |
| rs11893688 | 2 | 9695282 | √ |  | √ |  | T | C |
| rs2551347 | 2 | 23912401 |  |  | √ | √ | C | T |
| rs1179494 | 2 | 36809496 |  |  |  |  | G | C |
| rs754868 | 2 | 43185532 |  |  | √ | √ | A | G |
| rs4952673 | 2 | 43423870 | √ | √ |  |  | A | G |
| rs17034876 | 2 | 46484310 | √ |  | √ |  | T | C |
| rs4953353 | 2 | 46567276 |  |  | √ | √ | T | G |
| rs186606513 | 2 | 97482001 |  |  |  |  | G | A |
| rs56188432 | 2 | 158406865 |  |  | √ | √ | G | A |
| rs560887 | 2 | 169763148 | √ |  | √ |  | C | T |
| rs2280235 | 2 | 191843830 |  |  |  |  | G | A |
| rs10181515 | 2 | 227019461 |  |  | √ | √ | C | T |
| rs9855896 | 3 | 14287150 | √ |  | √ |  | G | A |
| rs2168443 | 3 | 46947087 | √ |  | √ |  | T | A |
| rs11708067 | 3 | 123065778 | √ |  | √ |  | A | G |
| rs9851257 | 3 | 123125711 | √ | √ |  |  | T | A |
| rs6440006 | 3 | 141142691 | √ | √ |  |  | A | G |
| rs2306700 | 3 | 142123841 |  |  |  |  | T | C |
| rs10935733 | 3 | 148622968 |  |  | √ | √ | C | T |
| rs4679760 | 3 | 155855418 | √ |  | √ |  | G | C |
| rs1482852 | 3 | 156798294 |  |  | √ | √ | G | A |
| rs11711420 | 3 | 183349010 |  |  | √ | √ | G | T |
| rs4144829 | 4 | 17903654 |  |  | √ | √ | C | T |
| rs2174633 | 4 | 17917781 | √ |  | √ |  | A | C |
| rs2189234 | 4 | 106075498 | √ | √ |  |  | G | T |
| rs6533183 | 4 | 106133184 |  |  |  |  | C | T |
| rs116807401 | 4 | 135121721 |  |  | √ | √ | T | C |
| rs6845999 | 4 | 145565826 | √ |  | √ |  | T | C |
| rs2131354 | 4 | 145599908 | √ |  | √ |  | A | G |
| rs4579095 | 4 | 174726635 | √ | √ |  |  | G | A |
| rs1818782 | 5 | 39424628 |  |  |  |  | A | C |
| rs351930 | 5 | 52003397 |  |  | √ | √ | A | T |
| rs854037 | 5 | 57091783 |  |  |  |  | A | G |
| rs28365970 | 5 | 67585723 |  |  |  |  | C | A |
| rs6871635 | 5 | 133830395 |  |  |  |  | G | A |
| rs1981627 | 5 | 133838180 |  |  |  |  | G | A |
| rs2946179 | 5 | 157886627 | √ | √ |  |  | C | T |
| rs34471628 | 5 | 172196752 | √ | √ |  |  | A | G |
| rs9379084 | 6 | 7231843 | √ | √ |  |  | G | A |
| rs35261542 | 6 | 20675792 | √ |  | √ |  | A | C |
| rs9379832 | 6 | 26186200 |  |  |  |  | A | G |
| rs9366778 | 6 | 31269173 |  |  |  |  | G | A |
| rs6911024 | 6 | 31368451 | √ | √ |  |  | T | C |
| rs9267812 | 6 | 32128394 | √ |  | √ |  |  |  |
| rs1547669 | 6 | 33775641 |  |  | √ | √ | A | G |
| rs75104038 | 6 | 34190104 | √ |  | √ |  | A | G |
| rs75034466 | 6 | 34199815 | √ |  | √ |  | T | C |
| rs6911621 | 6 | 35529025 |  |  |  |  | T | C |
| rs9348981 | 6 | 35687249 |  |  |  |  | T | G |
| rs7744700 | 6 | 53349401 |  |  |  |  | T | A |
| rs76094073 | 6 | 109288036 |  |  |  |  | G | C |
| rs6568554 | 6 | 109290319 |  |  |  |  | A | C |
| rs6925689 | 6 | 126865884 |  |  | √ | √ | C | T |
| rs6569647 | 6 | 130337266 |  |  |  |  | T | C |
| rs1415701 | 6 | 130345835 |  |  |  |  | G | A |
| rs6930558 | 6 | 141878920 |  |  | √ | √ | T | G |
| rs962554 | 6 | 142734204 |  |  |  |  | T | C |
| rs10872678 | 6 | 152039964 |  |  | √ | √ | T | C |
| rs7772579 | 6 | 152042502 |  |  | √ | √ | A | C |
| rs2934844 | 6 | 166142456 |  |  |  |  | T | A |
| rs1724889 | 7 | 2741021 |  |  |  |  | G | A |
| rs4719648 | 7 | 2756832 |  |  |  |  | C | T |
| rs59084784 | 7 | 22739562 |  |  |  |  | A | C |
| rs7808457 | 7 | 22798265 |  |  |  |  | A | T |
| rs34776209 | 7 | 23513093 | √ |  | √ |  | C | T |
| rs2908279 | 7 | 44174857 |  |  |  |  | T | G |
| rs2971669 | 7 | 44231778 | √ | √ |  |  | T | C |
| rs138715366 | 7 | 44246271 |  |  | √ | √ | C | T |
| rs10265133 | 7 | 45895604 | √ |  | √ |  | G | T |
| rs11983722 | 7 | 46298647 |  |  |  |  | A | T |
| rs10265057 | 7 | 47275737 |  |  | √ | √ | A | G |
| rs2237467 | 7 | 50733316 | √ |  | √ |  | A | G |
| rs112139215 | 7 | 73034559 |  |  | √ | √ | C | A |
| rs2282978 | 7 | 92264410 |  |  | √ | √ | T | C |
| rs45446698 | 7 | 99332948 | √ | √ |  |  | G | T |
| rs13231367 | 7 | 127509070 | √ |  | √ |  | G | A |
| rs6467157 | 7 | 127660763 | √ |  | √ |  | T | C |
| rs3918226 | 7 | 150690176 | √ | √ |  |  | C | T |
| rs62496903 | 8 | 6446938 |  |  |  |  | T | C |
| rs732563 | 8 | 23345526 |  |  | √ | √ | T | C |
| rs11778247 | 8 | 23403378 | √ | √ |  |  | G | A |
| rs34036147 | 8 | 38366249 |  |  | √ | √ | C | T |
| rs13266210 | 8 | 41533514 |  |  | √ | √ | G | A |
| rs72656010 | 8 | 57122215 |  |  | √ | √ | C | T |
| rs6995390 | 8 | 77611012 | √ |  | √ |  | T | A |
| rs7819593 | 8 | 106115172 |  |  | √ | √ | T | C |
| rs10283100 | 8 | 120596023 |  |  |  |  | G | A |
| rs13271368 | 8 | 126506140 |  |  |  |  | C | T |
| rs13257363 | 8 | 142252580 |  |  |  |  | G | A |
| rs9657468 | 8 | 142362391 |  |  | √ | √ | T | G |
| rs7854962 | 9 | 96900505 |  |  |  |  | C | G |
| rs28457693 | 9 | 98217348 |  |  | √ | √ | G | A |
| rs1411424 | 9 | 113892963 | √ |  | √ |  | A | G |
| rs2418135 | 9 | 113901309 | √ |  | √ |  | A | G |
| rs72760655 | 9 | 116916214 | √ |  | √ |  | C | A |
| rs1323438 | 9 | 119115531 |  |  | √ | √ | T | C |
| rs3933326 | 9 | 123633948 |  |  | √ | √ | A | G |
| rs10985827 | 9 | 125701608 |  |  |  |  | G | T |
| rs28505901 | 9 | 139241030 |  |  | √ | √ | A | G |
| rs4350272 | 10 | 25056118 |  |  |  |  | A | G |
| rs5030938 | 10 | 70975916 | √ |  | √ |  | T | C |
| rs9645500 | 10 | 70986723 | √ |  | √ |  | G | T |
| rs1112718 | 10 | 94479107 | √ |  | √ |  | A | G |
| rs10509669 | 10 | 95969913 | √ |  | √ |  | A | T |
| rs3740360 | 10 | 96025491 | √ | √ |  |  | C | A |
| rs2274224 | 10 | 96039597 |  |  |  |  | C | G |
| rs562974282 | 10 | 104201070 | √ |  | √ |  | G | T |
| rs10883846 | 10 | 104958244 |  |  |  |  | C | T |
| rs7903146 | 10 | 114758349 |  |  |  |  | T | C |
| rs7076938 | 10 | 115789375 |  |  | √ | √ | T | C |
| rs1801253 | 10 | 115805056 |  |  |  |  | C | G |
| rs71486610 | 10 | 124134803 | √ |  | √ |  | C | G |
| rs11042596 | 11 | 2118860 |  |  | √ | √ | G | T |
| rs234864 | 11 | 2857297 |  |  | √ | √ | G | A |
| rs2168101 | 11 | 8255408 | √ |  | √ |  | C | A |
| rs4444073 | 11 | 10331664 |  |  | √ | √ | C | A |
| rs12574749 | 11 | 32405355 |  |  |  |  | C | A |
| rs5030317 | 11 | 32410337 |  |  |  |  | C | G |
| rs10437653 | 11 | 46297631 |  |  |  |  | A | C |
| rs10734564 | 11 | 48160429 | √ | √ |  |  | A | G |
| rs667515 | 11 | 69449076 | √ |  | √ |  | G | C |
| rs61885091 | 11 | 69791952 |  |  |  |  | A | G |
| rs10830963 | 11 | 92708710 | √ | √ |  |  | G | C |
| rs10895278 | 11 | 102095335 | √ | √ |  |  | C | T |
| rs76895963 | 12 | 4384844 | √ |  | √ |  | G | T |
| rs11055030 | 12 | 12878349 |  |  | √ | √ | C | G |
| rs2306547 | 12 | 26877885 |  |  |  |  | C | T |
| rs11051061 | 12 | 30914668 | √ | √ |  |  | A | G |
| rs6582623 | 12 | 46613394 |  |  |  |  | C | T |
| rs180438 | 12 | 47187260 | √ | √ |  |  | G | A |
| rs8756 | 12 | 66359752 |  |  | √ | √ | C | A |
| rs7968682 | 12 | 66371880 |  |  | √ | √ | G | T |
| rs1480470 | 12 | 66412130 |  |  | √ | √ | A | G |
| rs1533688 | 12 | 102772745 | √ | √ |  |  | C | T |
| rs2647873 | 12 | 103081192 | √ |  | √ |  | A | G |
| rs17033114 | 12 | 103123339 | √ | √ |  |  | T | C |
| rs3184504 | 12 | 111884608 | √ | √ |  |  | C | T |
| rs9549046 | 13 | 40647206 |  |  |  |  | A | G |
| rs34217484 | 13 | 48854550 | √ |  | √ |  | A | T |
| rs9318511 | 13 | 78601413 |  |  |  |  | C | A |
| rs72681869 | 14 | 50655357 |  |  | √ | √ | G | C |
| rs6575803 | 14 | 101257755 |  |  | √ | √ | T | C |
| rs75844534 | 15 | 38667117 | √ |  | √ |  | C | A |
| rs2928148 | 15 | 41401550 | √ | √ |  |  | A | G |
| rs339969 | 15 | 60883281 | √ |  | √ |  | A | C |
| rs3784789 | 15 | 75082552 | √ |  | √ |  | G | C |
| rs12909648 | 15 | 86224570 | √ | √ |  |  | G | A |
| rs12443252 | 15 | 91064690 | √ | √ |  |  | T | C |
| rs7183988 | 15 | 91428589 |  |  |  |  | G | T |
| rs4932373 | 15 | 91429287 | √ |  | √ |  | A | C |
| rs55958435 | 15 | 96852638 |  |  |  |  | A | G |
| rs7402983 | 15 | 99193276 |  |  | √ | √ | A | C |
| rs11630479 | 15 | 99240481 |  |  |  |  | G | A |
| rs2045457 | 16 | 20046115 |  |  |  |  | G | A |
| rs40434 | 16 | 55699525 |  |  | √ | √ | A | G |
| rs28544888 | 16 | 55741204 |  |  |  |  | C | T |
| rs11641308 | 16 | 75312023 | √ | √ |  |  | T | C |
| rs222857 | 17 | 7164563 |  |  | √ | √ | T | C |
| rs2428362 | 17 | 7180274 |  |  |  |  | T | C |
| rs4511593 | 17 | 7455536 |  |  | √ | √ | C | T |
| rs78378222 | 17 | 7571752 |  |  |  |  | G | T |
| rs9909342 | 17 | 25652275 |  |  | √ | √ | A | G |
| rs7223535 | 17 | 29211667 |  |  |  |  | G | A |
| rs11867479 | 17 | 68090207 |  |  | √ | √ | C | T |
| rs10221267 | 17 | 68464662 |  |  | √ | √ | C | T |
| rs73354194 | 17 | 79905947 |  |  | √ | √ | T | C |
| rs9912553 | 17 | 79959703 |  |  |  |  | G | C |
| rs11082304 | 18 | 20720973 |  |  |  |  | T | G |
| rs2779165 | 19 | 4915447 |  |  |  |  | G | C |
| rs8106042 | 19 | 7161849 |  |  | √ | √ | C | G |
| rs2967676 | 19 | 8789666 | √ | √ |  |  | A | C |
| rs41355649 | 19 | 33790556 |  |  | √ | √ | A | G |
| rs1129156 | 19 | 40719076 |  |  | √ | √ | C | T |
| rs147957154 | 19 | 43431040 |  |  | √ | √ | C | T |
| rs516246 | 19 | 49206172 |  |  |  |  | C | T |
| rs255773 | 19 | 54723546 |  |  |  |  | C | T |
| rs147110934 | 19 | 55993436 |  |  |  |  | G | T |
| rs12461110 | 19 | 56320663 | √ | √ |  |  | A | G |
| rs304001 | 19 | 56423668 | √ | √ |  |  | G | A |
| rs6040076 | 20 | 10658882 |  |  |  |  | C | G |
| rs6033062 | 20 | 11207419 |  |  |  |  | A | T |
| rs1203876 | 20 | 22540915 | √ |  | √ |  | A | C |
| rs11698914 | 20 | 31327144 |  |  | √ | √ | C | G |
| rs181451002 | 20 | 32466219 | √ | √ |  |  | G | A |
| rs2889874 | 20 | 33715777 |  |  |  |  | G | T |
| rs1012167 | 20 | 39159119 |  |  | √ | √ | T | C |
| rs753381 | 20 | 39797465 |  |  | √ | √ | C | T |
| rs6026449 | 20 | 57272617 |  |  | √ | √ | T | C |
| rs73143584 | 20 | 62445702 |  |  | √ | √ | G | A |
| rs2229742 | 21 | 16339172 |  |  |  |  | G | C |
| rs220193 | 21 | 43581308 |  |  |  |  | G | A |
| rs134594 | 22 | 29468456 |  |  | √ | √ | T | C |
| rs41311445 | 22 | 42070374 |  |  | √ | √ | C | A |
| rs7285579 | 22 | 46441980 |  |  | √ | √ | T | C |

SNP: Single Nucleotide Polymorphism

^a :^ Effect allele: Birth weight increasing allele from the conditional analyses of maternal genotype on offspring birth weight by Warrington et al. (2019).

**Table S2.** Detailed information on the GWAS summary-statistic data for birth weight and bone parameters.

| **Phenotype** | **Sample size** | **Mean (Standard deviation)**  **Age (years)** | **Explained Variations** |
| --- | --- | --- | --- |
| Fetal birth weigh | 321,223 | - | 6% |
| Maternal birth weight | 230,069 | - | 2% |
| Femoral neck BMD | 49,988 | 67.97 (4.82) | - |
| Total hip area | 28,900 | - | - |
| Femoral neck area | 28,954 | - | - |
| Intertrochanteric area | 28,936 | - | - |
| Trochanter area | 28,944 | - | - |

**Table S3.** Characteristics of participants from the UK Biobank dataset in the observational analysis for the Spine DXA data.

| **Variable** | **Field ID** | **Participants No. (%) (n=20,367^a^)** | **Coding** |
| --- | --- | --- | --- |
| **Sex No. (%)** | 31 |  |  |
| Female |  | 11,793 (57.90) | 0 |
| Male |  | 8,574 (42.10) | 1 |
| **Age**, mean (SD)/years | 21003 | 53.78 (7.40) | - |
| **Weight**, mean (SD)/kg | 21001 | 76.25 (15.04) | - |
| **Smoking status, No. (%)** | 20116 |  |  |
| Never |  | 12,680 (62.26) | 0 |
| Previous |  | 6,504 (31.93) | 1 |
| Current |  | 1,183 (5.80) | 2 |
| **Drinking status, No. (%)** | 1558 |  |  |
| Daily or almost daily |  | 4,311 (21.17) | 1 |
| Three or four times a week |  | 5,799 (28.47) | 2 |
| Once or twice a week |  | 5,460 (26.81) | 3 |
| One to three times a month |  | 2,298 (11.28) | 4 |
| Special occasions only |  | 1,657 (8.14) | 5 |
| Never |  | 842 (4.13) | 6 |
| **Regular physical activity. (%)** | 884,894,904,914 |  |  |
| Yes |  | 15,291 (75.08) | 1 |
| No |  | 5,076 (24.92) | 0 |
| **Birth weigh**t, mean (SD)/kg | 20022 | 3.40 (0.41) | - |
| **LS BMD Mean (SD)/ g/cm2** | 23204 | 1.18 (0.20) | - |
| **LS BMC Mean (SD)/ g** | 23203 | 71.26 (18.78) | - |
| **LS area Mean (SD)/ cm2** | 23200 | 59.78 (8.27) | - |
| **LS aBMC** | 23203, 23200 | 71.26 (10.88) | - |
| **LaS BMD Mean (SD)/ g/cm2** | 23204 | 0.93 (0.14) | - |
| **LaS BMC Mean (SD)/ g** | 23234 | 212.89 (59.83) | - |
| **LaS area Mean (SD)/ cm2** | 23311 | 193.25 (28.31) | - |
| **LaS aBMC** | 23234, 23311 | 212.89 (29.33) |  |

**Note:** LS; lumbar spine, LaS; lateral spine.

**^a^ :** To keep the table simple and minimal, the number of individuals with LaS data we set the same as LS data.

**Table S4.** Characteristics of participants from the UK Biobank dataset in the observational analysis for the heel BMD measured by QUS.

| Variable | Field ID | Participants No. (%) (n=177,675) | Coding |
| --- | --- | --- | --- |
| **Sex No. (%)** | 31 |  |  |
| Female |  | 108,760 (61.21) | 0 |
| Male |  | 68,915 (38.79) | 1 |
| **Age**, mean (SD)/years | 21003 | 55.14 (8.12) | - |
| **weight**, mean (SD)/kg | 21001 | 77.16 (15.76) | - |
| **Smoking status, No. (%)** | 20116 |  |  |
| Never |  | 100,046 (56.30) | 0 |
| Previous |  | 60,364 (33.97) | 1 |
| Current |  | 17,265 (9.71) | 2 |
| **Drinking status, No. (%)** | 1558 |  |  |
| Daily or almost daily |  | 36,462 (20.52) | 1 |
| Three or four times a week |  | 43,520 (24.49) | 2 |
| Once or twice a week |  | 47,615 (26.80) | 3 |
| One to three times a month |  | 20,662 (11.62) | 4 |
| Special occasions only |  | 18,463 (10.39) | 5 |
| Never |  | 10,953 (6.16) | 6 |
| **Regular physical activity. (%)** | 884,894,904,914 |  |  |
| Yes |  | 130,275 (73.32) | 1 |
| No |  | 47,400 (26.68) | 0 |
| **Birth weight**, mean (SD) /kg | 20022 | 3.38 (0.42) | - |
| **Heel bone mineral density** (heel BMD), Mean (SD) / g/cm2 | 3148, 4105 | 0.54 (0.12) | - |

**Table S5.** Summary statistics for the 63 SNPs used in instrumental variable analysis to assess the effect of fetal birth weight on the hip bone parameters.

| **63 instrumental varibles** | | | | | **Fetal birth weight (Warrington et al. 2019)** | | | **Femoral neck BMD (Zheng. 2015)** | | | **total hip area (Styrkarsdottir et al. 2019)** | | | **Femoral neck area (Styrkarsdottir et al. 2019)** | | | **Intertrochanteric area (Styrkarsdottir et al. 2019)** | | | **Trochanter area (Styrkarsdottir et al. 2019)** | | |
| --- | --- | --- | --- | --- | --- | --- | --- | --- | --- | --- | --- | --- | --- | --- | --- | --- | --- | --- | --- | --- | --- | --- |
| **SNP** | **CHR** | | **BP** | **EA** | **β** | **SE** | ***P*** | **β** | **SE** | ***P*** | **β** | **SE** | ***P*** | **β** | **SE** | ***P*** | **β** | **SE** | ***P*** | **β** | **SE** | ***P*** |
| rs12401656 | | 1 | 43456767 | G | 0.029 | 0.006 | 1.60E-06 | -0.017 | 0.011 | 0.13 | -0.013 | 0.012 | 0.27 | -0.027 | 0.012 | 0.03 | -0.01 | 0.012 | 0.41 | -0.01 | 0.012 | 0.43 |
| rs80278614 | | 1 | 119412317 | A | 0.052 | 0.009 | 4.00E-08 | -0.024 | 0.017 | 0.17 | -0.006 | 0.008 | 0.46 | -0.012 | 0.009 | 0.16 | 0 | 0.008 | 0.97 | -0.01 | 0.008 | 0.22 |
| rs61830764 | | 1 | 212289976 | A | 0.018 | 0.004 | 3.00E-05 | -0.011 | 0.008 | 0.17 | 0.017 | 0.016 | 0.31 | -0.05 | 0.017 | 0 | 0.031 | 0.017 | 0.07 | -0.008 | 0.016 | 0.63 |
| rs2551347 | | 2 | 23912401 | T | 0.029 | 0.005 | 2.20E-09 | -0.014 | 0.009 | 0.11 | 0.004 | 0.009 | 0.66 | -0.013 | 0.01 | 0.19 | 0.002 | 0.009 | 0.83 | 0.007 | 0.009 | 0.46 |
| rs754868 | | 2 | 43185532 | G | 0.019 | 0.004 | 4.70E-06 | 0.007 | 0.008 | 0.39 | 0.002 | 0.009 | 0.83 | -0.002 | 0.009 | 0.83 | -0.005 | 0.009 | 0.62 | 0.012 | 0.01 | 0.22 |
| rs4953353 | | 2 | 46567276 | G | 0.019 | 0.004 | 6.50E-06 | 0.01 | 0.008 | 0.21 | -0.002 | 0.008 | 0.84 | -0.001 | 0.008 | 0.94 | -0.001 | 0.008 | 0.9 | -0.002 | 0.008 | 0.79 |
| rs56188432 | | 2 | 158406865 | G | 0.250 | 0.048 | 2.64E-07 | - | - | - | 0.19 | 0.063 | 0 | 0.073 | 0.064 | 0.26 | 0.187 | 0.064 | 0 | 0.088 | 0.064 | 0.17 |
| rs10181515 | | 2 | 227019461 | T | 0.021 | 0.005 | 1.30E-05 | 0.023 | 0.009 | 0.01 | -0.002 | 0.008 | 0.82 | 0.012 | 0.008 | 0.16 | 0.001 | 0.008 | 0.92 | -0.008 | 0.008 | 0.33 |
| rs10935733 | | 3 | 148622968 | T | 0.021 | 0.004 | 5.00E-07 | 0.019 | 0.008 | 0.03 | -0.011 | 0.008 | 0.19 | -0.005 | 0.008 | 0.57 | -0.015 | 0.008 | 0.07 | 0.002 | 0.008 | 0.78 |
| rs1482852 | | 3 | 156798294 | A | 0.054 | 0.004 | 7.60E-39 | -0.019 | 0.008 | 0.02 | 0.002 | 0.009 | 0.8 | -0.015 | 0.01 | 0.14 | 0.007 | 0.01 | 0.46 | -0.008 | 0.01 | 0.41 |
| rs11711420 | | 3 | 183349010 | T | 0.022 | 0.005 | 2.70E-06 | -0.01 | 0.009 | 0.26 | 0.026 | 0.009 | 0 | 0.026 | 0.009 | 0 | 0.02 | 0.009 | 0.02 | 0.015 | 0.009 | 0.09 |
| rs4144829 | | 4 | 17903654 | C | 0.032 | 0.005 | 1.10E-11 | 0.009 | 0.009 | 0.33 | 0.069 | 0.038 | 0.07 | 0.024 | 0.037 | 0.54 | 0.048 | 0.038 | 0.2 | 0.069 | 0.038 | 0.07 |
| rs116807401 | | 4 | 135121721 | C | 0.088 | 0.016 | 7.00E-08 | 0.035 | 0.031 | 0.27 | 0.028 | 0.009 | 0 | 0.032 | 0.009 | 0 | 0.018 | 0.009 | 0.05 | 0.025 | 0.009 | 0.01 |
| rs351930 | | 5 | 52003397 | T | 0.02 | 0.005 | 8.10E-05 | -0.016 | 0.009 | 0.08 | 0.007 | 0.01 | 0.47 | 0.033 | 0.01 | 0 | -0.001 | 0.009 | 0.95 | 0.007 | 0.01 | 0.51 |
| rs1547669 | | 6 | 33775641 | G | 0.018 | 0.004 | 9.30E-06 | -0.016 | 0.007 | 0.03 | -0.005 | 0.009 | 0.6 | -0.008 | 0.009 | 0.36 | -0.006 | 0.009 | 0.51 | 0.005 | 0.009 | 0.59 |
| rs6925689 | | 6 | 126865884 | T | 0.018 | 0.004 | 2.10E-05 | -0.029 | 0.008 | 0 | 0 | 0.196 | 1 | -0.017 | 0.008 | 0.05 | -0.001 | 0.008 | 0.88 | 0.007 | 0.008 | 0.43 |
| rs6930558 | | 6 | 141878920 | T | 0.022 | 0.005 | 3.30E-06 | -0.004 | 0.009 | 0.69 | 0.002 | 0.008 | 0.8 | 0.004 | 0.008 | 0.61 | 0 | 0.007 | 0.99 | 0.001 | 0.008 | 0.94 |
| rs10872678 | | 6 | 152039964 | T | 0.028 | 0.005 | 8.20E-10 | -0.015 | 0.009 | 0.08 | 0.029 | 0.009 | 0 | 0.028 | 0.01 | 0 | 0.021 | 0.009 | 0.02 | 0.015 | 0.009 | 0.11 |
| rs7772579 | | 6 | 152042502 | A | 0.027 | 0.005 | 5.80E-09 | -0.022 | 0.01 | 0.03 | -0.004 | 0.009 | 0.65 | -0.007 | 0.009 | 0.42 | -0.006 | 0.009 | 0.49 | 0.007 | 0.009 | 0.46 |
| rs138715366 | | 7 | 44246271 | C | 0.235 | 0.022 | 1.40E-25 | 0.005 | 0.046 | 0.92 | -0.003 | 0.012 | 0.79 | -0.002 | 0.012 | 0.9 | 0.003 | 0.012 | 0.8 | -0.016 | 0.013 | 0.21 |
| rs10265057 | | 7 | 47275737 | G | 0.036 | 0.007 | 4.60E-07 | 0.004 | 0.013 | 0.76 | -0.029 | 0.016 | 0.06 | -0.017 | 0.016 | 0.28 | -0.025 | 0.016 | 0.11 | -0.018 | 0.016 | 0.26 |
| rs112139215 | | 7 | 73034559 | A | 0.056 | 0.008 | 1.20E-11 | 0.015 | 0.015 | 0.32 | 0.061 | 0.064 | 0.34 | 0.053 | 0.064 | 0.42 | 0.019 | 0.061 | 0.77 | 0.123 | 0.065 | 0.06 |
| rs2282978 | | 7 | 92264410 | C | 0.021 | 0.004 | 1.60E-06 | 0.014 | 0.008 | 0.08 | 0.013 | 0.009 | 0.14 | 0.002 | 0.009 | 0.86 | 0.016 | 0.009 | 0.08 | -0.002 | 0.008 | 0.86 |
| rs732563 | | 8 | 23345526 | C | 0.019 | 0.004 | 6.10E-06 | 0.005 | 0.007 | 0.55 | -0.019 | 0.011 | 0.09 | 0.001 | 0.01 | 0.96 | -0.007 | 0.011 | 0.55 | -0.028 | 0.011 | 0.01 |
| rs34036147 | | 8 | 38366249 | T | 0.019 | 0.004 | 1.60E-05 | -0.011 | 0.008 | 0.22 | -0.009 | 0.009 | 0.34 | -0.004 | 0.009 | 0.68 | -0.013 | 0.009 | 0.16 | 0.005 | 0.009 | 0.57 |
| rs13266210 | | 8 | 41533514 | A | 0.03 | 0.005 | 3.10E-09 | 0.01 | 0.009 | 0.26 | 0.035 | 0.013 | 0.01 | 0.038 | 0.013 | 0 | 0.02 | 0.013 | 0.12 | 0.033 | 0.013 | 0.01 |
| rs72656010 | | 8 | 57122215 | T | 0.026 | 0.006 | 1.60E-05 | -0.006 | 0.011 | 0.59 | -0.005 | 0.008 | 0.58 | -0.011 | 0.008 | 0.17 | -0.007 | 0.008 | 0.39 | 0.007 | 0.008 | 0.42 |
| rs7819593 | | 8 | 106115172 | C | 0.023 | 0.005 | 2.10E-06 | 0.007 | 0.009 | 0.42 | -0.003 | 0.009 | 0.77 | -0.004 | 0.01 | 0.66 | -0.004 | 0.009 | 0.68 | -0.001 | 0.009 | 0.88 |
| rs9657468 | | 8 | 142362391 | G | 0.018 | 0.004 | 3.60E-05 | -0.01 | 0.008 | 0.23 | -0.012 | 0.009 | 0.16 | 0.013 | 0.009 | 0.14 | -0.016 | 0.009 | 0.07 | 0.001 | 0.008 | 0.92 |
| rs28457693 | | 9 | 98217348 | G | 0.04 | 0.007 | 1.70E-09 | -0.021 | 0.012 | 0.09 | 0.024 | 0.009 | 0.01 | 0.011 | 0.009 | 0.21 | 0.022 | 0.009 | 0.02 | 0.014 | 0.009 | 0.11 |
| rs1323438 | | 9 | 119115531 | C | 0.02 | 0.005 | 1.30E-05 | -0.001 | 0.008 | 0.9 | 0.007 | 0.011 | 0.51 | -0.008 | 0.011 | 0.48 | -0.002 | 0.01 | 0.89 | 0.019 | 0.011 | 0.1 |
| rs3933326 | | 9 | 123633948 | G | 0.023 | 0.004 | 2.20E-07 | 0.014 | 0.008 | 0.11 | -0.004 | 0.01 | 0.72 | 0.009 | 0.01 | 0.37 | -0.002 | 0.009 | 0.88 | -0.007 | 0.01 | 0.49 |
| rs28505901 | | 9 | 139241030 | A | 0.024 | 0.005 | 4.20E-07 | -0.019 | 0.009 | 0.05 | 0.002 | 0.008 | 0.84 | -0.018 | 0.009 | 0.04 | 0.006 | 0.009 | 0.49 | -0.006 | 0.009 | 0.49 |
| rs7076938 | | 10 | 115789375 | T | 0.029 | 0.005 | 2.90E-10 | -0.001 | 0.009 | 0.87 | -0.004 | 0.01 | 0.71 | -0.03 | 0.01 | 0 | 0.002 | 0.009 | 0.87 | -0.005 | 0.01 | 0.63 |
| rs11042596 | | 11 | 2118860 | T | 0.027 | 0.004 | 1.60E-09 | -0.014 | 0.008 | 0.09 | 0.006 | 0.009 | 0.53 | -0.021 | 0.009 | 0.02 | 0.009 | 0.009 | 0.31 | -0.003 | 0.009 | 0.74 |
| rs234864 | | 11 | 2857297 | A | 0.017 | 0.004 | 4.90E-05 | 0.012 | 0.008 | 0.12 | - | - | - | - | - | - | - | - | - | - | - | - |
| rs4444073 | | 11 | 10331664 | A | 0.023 | 0.004 | 2.20E-08 | 0.006 | 0.008 | 0.46 | -0.004 | 0.008 | 0.62 | -0.006 | 0.008 | 0.5 | 0.004 | 0.008 | 0.67 | -0.011 | 0.008 | 0.17 |
| rs11055030 | | 12 | 12878349 | G | 0.022 | 0.005 | 1.00E-06 | 0.005 | 0.008 | 0.53 | -0.006 | 0.009 | 0.51 | 0.001 | 0.009 | 0.92 | -0.007 | 0.009 | 0.47 | -0.006 | 0.009 | 0.55 |
| rs8756 | | 12 | 66359752 | C | 0.037 | 0.004 | 1.70E-19 | 0.008 | 0.007 | 0.31 | 0.019 | 0.008 | 0.02 | 0.016 | 0.009 | 0.06 | 0.024 | 0.009 | 0.01 | -0.002 | 0.008 | 0.81 |
| rs7968682 | | 12 | 66371880 | G | 0.037 | 0.004 | 4.90E-20 | 0.008 | 0.008 | 0.31 | 0.01 | 0.008 | 0.22 | -0.003 | 0.008 | 0.74 | 0.01 | 0.008 | 0.23 | 0.006 | 0.008 | 0.45 |
| rs1480470 | | 12 | 66412130 | G | 0.028 | 0.004 | 1.10E-10 | -0.001 | 0.008 | 0.91 | 0.01 | 0.008 | 0.23 | -0.003 | 0.008 | 0.74 | 0.01 | 0.008 | 0.24 | 0.006 | 0.008 | 0.49 |
| rs72681869 | | 14 | 50655357 | C | 0.108 | 0.021 | 2.70E-07 | -0.05 | 0.048 | 0.31 | 0.006 | 0.011 | 0.62 | 0.014 | 0.012 | 0.22 | 0.001 | 0.011 | 0.96 | 0.007 | 0.011 | 0.57 |
| rs6575803 | | 14 | 101257755 | C | 0.034 | 0.007 | 9.90E-07 | 0.01 | 0.013 | 0.46 | 0.013 | 0.036 | 0.72 | 0.029 | 0.037 | 0.45 | 0.032 | 0.037 | 0.39 | -0.026 | 0.037 | 0.5 |
| rs7402983 | | 15 | 99193276 | A | 0.027 | 0.004 | 4.60E-10 | -0.003 | 0.011 | 0.77 | 0.007 | 0.008 | 0.43 | 0.014 | 0.008 | 0.11 | 0.001 | 0.008 | 0.88 | 0.007 | 0.008 | 0.38 |
| rs40434 | | 16 | 55699525 | G | 0.017 | 0.004 | 4.80E-05 | -0.002 | 0.008 | 0.76 | -0.006 | 0.008 | 0.47 | 0.001 | 0.008 | 0.94 | 0 | 0.008 | 0.98 | -0.014 | 0.008 | 0.08 |
| rs222857 | | 17 | 7164563 | T | 0.026 | 0.004 | 5.80E-10 | -0.007 | 0.008 | 0.41 | -0.002 | 0.008 | 0.79 | -0.008 | 0.008 | 0.35 | -0.002 | 0.008 | 0.8 | 0.007 | 0.008 | 0.39 |
| rs4511593 | | 17 | 7455536 | T | 0.019 | 0.004 | 7.40E-06 | 0.001 | 0.008 | 0.93 | 0.003 | 0.008 | 0.7 | -0.007 | 0.009 | 0.43 | -0.006 | 0.008 | 0.52 | 0.024 | 0.009 | 0.01 |
| rs9909342 | | 17 | 25652275 | A | 0.019 | 0.004 | 6.70E-06 | 0.003 | 0.008 | 0.71 | -0.01 | 0.008 | 0.23 | 0.001 | 0.008 | 0.88 | -0.003 | 0.008 | 0.72 | -0.016 | 0.008 | 0.05 |
| rs11867479 | | 17 | 68090207 | T | 0.018 | 0.004 | 2.20E-05 | -0.01 | 0.008 | 0.23 | 0.005 | 0.008 | 0.59 | 0.019 | 0.009 | 0.03 | 0.002 | 0.008 | 0.84 | 0.004 | 0.008 | 0.66 |
| rs10221267 | | 17 | 68464662 | T | 0.018 | 0.004 | 1.90E-05 | -0.022 | 0.008 | 0 | -0.027 | 0.027 | 0.33 | 0.029 | 0.028 | 0.3 | -0.047 | 0.028 | 0.09 | 0.008 | 0.026 | 0.77 |
| rs73354194 | | 17 | 79905947 | C | 0.06 | 0.014 | 1.70E-05 | -0.019 | 0.026 | 0.48 | 0.015 | 0.008 | 0.07 | -0.008 | 0.008 | 0.33 | 0.01 | 0.008 | 0.25 | 0.018 | 0.009 | 0.03 |
| rs8106042 | | 19 | 7161849 | G | 0.023 | 0.005 | 6.60E-07 | 0.009 | 0.009 | 0.32 | -0.013 | 0.009 | 0.15 | -0.008 | 0.009 | 0.41 | -0.007 | 0.009 | 0.46 | -0.018 | 0.009 | 0.06 |
| rs41355649 | | 19 | 33790556 | G | 0.042 | 0.008 | 4.50E-07 | -0.011 | 0.016 | 0.49 | 0.012 | 0.016 | 0.49 | 0.029 | 0.017 | 0.09 | 0.018 | 0.017 | 0.29 | -0.017 | 0.017 | 0.31 |
| rs1129156 | | 19 | 40719076 | T | 0.022 | 0.005 | 1.90E-06 | -0.015 | 0.008 | 0.08 | 0.008 | 0.009 | 0.4 | 0.01 | 0.009 | 0.29 | 0.004 | 0.009 | 0.68 | 0.01 | 0.009 | 0.27 |
| rs147957154 | | 19 | 43431040 | T | 0.026 | 0.006 | 2.40E-05 | 0.001 | 0.011 | 0.9 | 0.005 | 0.008 | 0.56 | - | - | - | - | - | - | - | - | - |
| rs11698914 | | 20 | 31327144 | C | 0.029 | 0.005 | 2.80E-09 | -0.002 | 0.009 | 0.81 | - | - | - | -0.011 | 0.009 | 0.19 | 0.008 | 0.008 | 0.33 | 0 | 0.008 | 0.97 |
| rs1012167 | | 20 | 39159119 | C | 0.024 | 0.004 | 1.90E-08 | -0.009 | 0.008 | 0.24 | 0.018 | 0.01 | 0.06 | 0 | 0.009 | 0.97 | 0.017 | 0.01 | 0.09 | 0.011 | 0.01 | 0.28 |
| rs753381 | | 20 | 39797465 | T | 0.018 | 0.004 | 9.10E-06 | -0.003 | 0.007 | 0.69 | -0.004 | 0.008 | 0.66 | -0.013 | 0.008 | 0.11 | 0 | 0.008 | 0.98 | -0.004 | 0.008 | 0.6 |
| rs6026449 | | 20 | 57272617 | C | 0.018 | 0.004 | 3.20E-05 | 0 | 0.008 | 0.99 | -0.002 | 0.011 | 0.88 | -0.004 | 0.011 | 0.73 | 0.003 | 0.011 | 0.8 | -0.011 | 0.012 | 0.35 |
| rs73143584 | | 20 | 62445702 | A | 0.031 | 0.007 | 3.30E-06 | 0.001 | 0.012 | 0.91 | 0.014 | 0.008 | 0.08 | 0.011 | 0.008 | 0.21 | 0.011 | 0.008 | 0.17 | 0.008 | 0.008 | 0.31 |
| rs134594 | | 22 | 29468456 | C | 0.022 | 0.004 | 6.00E-07 | 0.002 | 0.01 | 0.82 | 0 | 0.008 | 0.96 | 0.012 | 0.009 | 0.16 | 0.001 | 0.008 | 0.91 | -0.006 | 0.008 | 0.52 |
| rs41311445 | | 22 | 42070374 | A | 0.034 | 0.007 | 1.30E-06 | -0.009 | 0.015 | 0.57 | 0.013 | 0.013 | 0.31 | 0.011 | 0.013 | 0.4 | 0.007 | 0.013 | 0.61 | 0.012 | 0.013 | 0.36 |
| rs7285579 | | 22 | 46441980 | C | 0.018 | 0.005 | 0.00011 | -0.013 | 0.009 | 0.18 | 0.028 | 0.009 | 0 | 0.016 | 0.009 | 0.09 | 0.018 | 0.009 | 0.05 | 0.023 | 0.009 | 0.01 |

**Table S6.** Summary statistics for the 104 SNPs used in instrumental variable analysis to assess the effect of fetal birth weight on the hip bone parameters.

| **104 instrument varibles** | | | | | **Fetal birth weight (Warrington et al. 2019)** | | | **Femoral neck BMD (Zheng. 2015)** | | | **total hip area (Styrkarsdottir et al. 2019)** | | | **Femoral neck area (Styrkarsdottir et al. 2019)** | | | **Intertrochanteric area (Styrkarsdottir et al. 2019)** | | | **Trochanter area (Styrkarsdottir et al. 2019)** | | |
| --- | --- | --- | --- | --- | --- | --- | --- | --- | --- | --- | --- | --- | --- | --- | --- | --- | --- | --- | --- | --- | --- | --- |
| **SNP** | **CHR** | | **BP** | **EA** | **β** | **SE** | ***P*** | **β** | **SE** | ***P*** | **β** | **SE** | ***P*** | **β** | **SE** | ***P*** | **β** | **SE** | ***P*** | **β** | **SE** | ***P*** |
| rs12401656 | | 1 | 43456767 | G | 0.029 | 0.006 | 1.56E-06 | -0.017 | 0.011 | 0.13 | 0.059 | 0.032 | 0.06 | -0.007 | 0.03 | 0.83 | 0.024 | 0.031 | 0.45 | 0.082 | 0.032 | 0.01 |
| rs80278614 | | 1 | 119412317 | A | 0.052 | 0.009 | 4.03E-08 | -0.024 | 0.017 | 0.17 | -0.013 | 0.012 | 0.27 | -0.027 | 0.012 | 0.03 | -0.01 | 0.012 | 0.41 | -0.01 | 0.012 | 0.43 |
| rs10913200 | | 1 | 176521655 | G | 0.038 | 0.013 | 2.64E-03 | -0.007 | 0.024 | 0.78 | -0.006 | 0.008 | 0.46 | -0.012 | 0.009 | 0.16 | 0 | 0.008 | 0.97 | -0.01 | 0.008 | 0.22 |
| rs61830764 | | 1 | 212289976 | A | 0.018 | 0.004 | 3.00E-05 | -0.011 | 0.008 | 0.17 | 0.017 | 0.016 | 0.31 | -0.05 | 0.017 | 0 | 0.031 | 0.017 | 0.07 | -0.008 | 0.016 | 0.63 |
| rs10495563 | | 2 | 9662210 | A | 0.016 | 0.004 | 2.82E-04 | -0.018 | 0.008 | 0.03 | 0.004 | 0.009 | 0.66 | -0.013 | 0.01 | 0.19 | 0.002 | 0.009 | 0.83 | 0.007 | 0.009 | 0.46 |
| rs11893688 | | 2 | 9695282 | T | 0.015 | 0.004 | 4.52E-04 | -0.016 | 0.008 | 0.05 | 0.016 | 0.009 | 0.06 | 0.018 | 0.009 | 0.04 | 0.007 | 0.009 | 0.4 | 0.018 | 0.009 | 0.04 |
| rs2551347 | | 2 | 23912401 | T | 0.029 | 0.005 | 2.20E-09 | -0.014 | 0.009 | 0.11 | 0.015 | 0.009 | 0.09 | 0.015 | 0.009 | 0.1 | 0.005 | 0.009 | 0.57 | 0.02 | 0.009 | 0.03 |
| rs754868 | | 2 | 43185532 | G | 0.019 | 0.004 | 4.72E-06 | 0.007 | 0.008 | 0.39 | -0.011 | 0.009 | 0.23 | 0.02 | 0.009 | 0.03 | -0.02 | 0.009 | 0.03 | 0.009 | 0.009 | 0.32 |
| rs17034876 | | 2 | 46484310 | T | 0.039 | 0.005 | 5.47E-17 | 0.01 | 0.008 | 0.25 | 0.002 | 0.009 | 0.83 | -0.002 | 0.009 | 0.83 | -0.005 | 0.009 | 0.62 | 0.012 | 0.01 | 0.22 |
| rs4953353 | | 2 | 46567276 | G | 0.019 | 0.004 | 6.45E-06 | 0.01 | 0.008 | 0.21 | -0.002 | 0.008 | 0.84 | -0.001 | 0.008 | 0.94 | -0.001 | 0.008 | 0.9 | -0.002 | 0.008 | 0.79 |
| rs56188432 | | 2 | 158406865 | G | 0.250 | 0.049 | 2.64E-07 | - | - | - | -0.01 | 0.009 | 0.27 | 0 | 0.008 | 0.97 | -0.011 | 0.009 | 0.24 | -0.003 | 0.009 | 0.74 |
| rs560887 | | 2 | 169763148 | C | -0.025 | 0.004 | 2.78E-08 | 0.001 | 0.008 | 0.87 | 0.19 | 0.063 | 0 | 0.073 | 0.064 | 0.26 | 0.187 | 0.064 | 0 | 0.088 | 0.064 | 0.17 |
| rs10181515 | | 2 | 227019461 | T | 0.021 | 0.005 | 1.30E-05 | 0.023 | 0.009 | 0.01 | -0.002 | 0.008 | 0.82 | 0.012 | 0.008 | 0.16 | 0.001 | 0.008 | 0.92 | -0.008 | 0.008 | 0.33 |
| rs9855896 | | 3 | 14287150 | G | -0.014 | 0.005 | 6.14E-03 | 0.018 | 0.009 | 0.06 | -0.011 | 0.008 | 0.19 | -0.005 | 0.008 | 0.57 | -0.015 | 0.008 | 0.07 | 0.002 | 0.008 | 0.78 |
| rs2168443 | | 3 | 46947087 | T | 0.01 | 0.004 | 1.50E-02 | 0.003 | 0.008 | 0.7 | 0.008 | 0.01 | 0.44 | 0.004 | 0.01 | 0.67 | 0.008 | 0.01 | 0.44 | -0.001 | 0.009 | 0.89 |
| rs11708067 | | 3 | 123065778 | G | 0.056 | 0.005 | 6.26E-32 | -0.012 | 0.009 | 0.19 | 0.002 | 0.009 | 0.8 | -0.015 | 0.01 | 0.14 | 0.007 | 0.01 | 0.46 | -0.008 | 0.01 | 0.41 |
| rs10935733 | | 3 | 148622968 | T | 0.021 | 0.004 | 5.04E-07 | 0.019 | 0.008 | 0.03 | 0.026 | 0.009 | 0 | 0.026 | 0.009 | 0 | 0.02 | 0.009 | 0.02 | 0.015 | 0.009 | 0.09 |
| rs4679760 | | 3 | 155855418 | G | -0.009 | 0.004 | 3.33E-02 | -0.003 | 0.008 | 0.68 | -0.023 | 0.009 | 0.01 | 0.001 | 0.008 | 0.94 | -0.027 | 0.009 | 0 | -0.005 | 0.008 | 0.55 |
| rs1482852 | | 3 | 156798294 | A | 0.054 | 0.004 | 7.56E-39 | -0.019 | 0.008 | 0.02 | 0.009 | 0.008 | 0.3 | -0.004 | 0.008 | 0.65 | 0.011 | 0.008 | 0.2 | 0.005 | 0.008 | 0.55 |
| rs11711420 | | 3 | 183349010 | T | 0.022 | 0.005 | 2.67E-06 | -0.01 | 0.009 | 0.26 | 0.002 | 0.009 | 0.84 | 0 | 0.009 | 0.97 | 0.006 | 0.01 | 0.52 | -0.004 | 0.009 | 0.71 |
| rs4144829 | | 4 | 17903654 | C | 0.032 | 0.005 | 1.12E-11 | 0.009 | 0.009 | 0.33 | 0.069 | 0.038 | 0.07 | 0.024 | 0.037 | 0.54 | 0.048 | 0.038 | 0.2 | 0.069 | 0.038 | 0.07 |
| rs2174633 | | 4 | 17917781 | A | 0.031 | 0.005 | 3.62E-11 | 0.009 | 0.009 | 0.29 | 0.038 | 0.008 | 0 | 0.06 | 0.008 | 0 | 0.026 | 0.008 | 0 | 0.021 | 0.008 | 0.01 |
| rs116807401 | | 4 | 135121721 | C | 0.088 | 0.016 | 6.99E-08 | 0.035 | 0.031 | 0.27 | 0.029 | 0.009 | 0 | 0.031 | 0.009 | 0 | 0.019 | 0.009 | 0.03 | 0.024 | 0.009 | 0.01 |
| rs6845999 | | 4 | 145565826 | T | 0.017 | 0.004 | 2.80E-05 | -0.01 | 0.008 | 0.19 | 0.028 | 0.009 | 0 | 0.032 | 0.009 | 0 | 0.018 | 0.009 | 0.05 | 0.025 | 0.009 | 0.01 |
| rs2131354 | | 4 | 145599908 | A | 0.016 | 0.004 | 1.86E-04 | -0.01 | 0.008 | 0.18 | 0.033 | 0.008 | 0 | 0.059 | 0.008 | 0 | 0.019 | 0.008 | 0.02 | 0.023 | 0.008 | 0 |
| rs351930 | | 5 | 52003397 | T | 0.02 | 0.005 | 8.10E-05 | -0.016 | 0.009 | 0.08 | 0.007 | 0.01 | 0.47 | 0.033 | 0.01 | 0 | -0.001 | 0.009 | 0.95 | 0.007 | 0.01 | 0.51 |
| rs35261542 | | 6 | 20675792 | C | 0.049 | 0.005 | 3.23E-26 | -0.006 | 0.009 | 0.5 | -0.005 | 0.009 | 0.6 | -0.008 | 0.009 | 0.36 | -0.006 | 0.009 | 0.51 | 0.005 | 0.009 | 0.59 |
| rs9267812 | | 6 | 32128394 | T | 0.015 | 0.006 | 1.25E-02 | -0.024 | 0.014 | 0.09 | 0 | 0.196 | 1 | -0.017 | 0.008 | 0.05 | -0.001 | 0.008 | 0.88 | 0.007 | 0.008 | 0.43 |
| rs1547669 | | 6 | 33775641 | G | 0.018 | 0.004 | 9.28E-06 | -0.016 | 0.007 | 0.03 | -0.013 | 0.01 | 0.18 | -0.012 | 0.01 | 0.21 | -0.01 | 0.01 | 0.33 | -0.004 | 0.009 | 0.67 |
| rs75104038 | | 6 | 34190104 | A | 0.024 | 0.009 | 5.96E-03 | -0.001 | 0.016 | 0.94 | 0.002 | 0.008 | 0.8 | 0.004 | 0.008 | 0.61 | 0 | 0.007 | 0.99 | 0.001 | 0.008 | 0.94 |
| rs75034466 | | 6 | 34199815 | T | 0.02 | 0.01 | 4.01E-02 | 0.008 | 0.018 | 0.67 | 0.029 | 0.009 | 0 | 0.028 | 0.01 | 0 | 0.021 | 0.009 | 0.02 | 0.015 | 0.009 | 0.11 |
| rs6925689 | | 6 | 126865884 | T | 0.018 | 0.004 | 2.10E-05 | -0.029 | 0.008 | 0 | 0.045 | 0.023 | 0.04 | 0.022 | 0.023 | 0.34 | 0.037 | 0.023 | 0.11 | 0.033 | 0.023 | 0.14 |
| rs6930558 | | 6 | 141878920 | T | 0.022 | 0.005 | 3.34E-06 | -0.004 | 0.009 | 0.69 | 0.046 | 0.021 | 0.03 | 0.017 | 0.021 | 0.43 | 0.035 | 0.021 | 0.1 | 0.042 | 0.021 | 0.05 |
| rs10872678 | | 6 | 152039964 | T | 0.028 | 0.005 | 8.23E-10 | -0.015 | 0.009 | 0.08 | -0.004 | 0.009 | 0.65 | -0.007 | 0.009 | 0.42 | -0.006 | 0.009 | 0.49 | 0.007 | 0.009 | 0.46 |
| rs7772579 | | 6 | 152042502 | A | 0.027 | 0.005 | 5.84E-09 | -0.022 | 0.01 | 0.03 | -0.026 | 0.012 | 0.03 | -0.006 | 0.012 | 0.61 | -0.028 | 0.012 | 0.02 | -0.002 | 0.011 | 0.89 |
| rs34776209 | | 7 | 23513093 | C | 0.015 | 0.005 | 1.30E-03 | -0.014 | 0.009 | 0.12 | -0.003 | 0.012 | 0.79 | -0.002 | 0.012 | 0.9 | 0.003 | 0.012 | 0.8 | -0.016 | 0.013 | 0.21 |
| rs138715366 | | 7 | 44246271 | C | 0.235 | 0.022 | 1.43E-25 | 0.005 | 0.046 | 0.92 | 0.001 | 0.01 | 0.95 | -0.015 | 0.011 | 0.18 | 0.007 | 0.011 | 0.49 | -0.007 | 0.011 | 0.52 |
| rs10265133 | | 7 | 45895604 | G | -0.02 | 0.006 | 9.74E-04 | -0.02 | 0.012 | 0.08 | -0.029 | 0.016 | 0.06 | -0.017 | 0.016 | 0.28 | -0.025 | 0.016 | 0.11 | -0.018 | 0.016 | 0.26 |
| rs10265057 | | 7 | 47275737 | G | 0.036 | 0.007 | 4.58E-07 | 0.004 | 0.013 | 0.76 | 0.003 | 0.008 | 0.7 | 0.006 | 0.009 | 0.49 | 0.003 | 0.008 | 0.76 | -0.001 | 0.008 | 0.87 |
| rs2237467 | | 7 | 50733316 | A | 0.011 | 0.005 | 2.16E-02 | -0.012 | 0.009 | 0.18 | 0.061 | 0.064 | 0.34 | 0.053 | 0.064 | 0.42 | 0.019 | 0.061 | 0.77 | 0.123 | 0.065 | 0.06 |
| rs112139215 | | 7 | 73034559 | A | 0.056 | 0.008 | 1.20E-11 | 0.015 | 0.015 | 0.32 | 0.001 | 0.009 | 0.91 | 0.014 | 0.01 | 0.19 | -0.002 | 0.01 | 0.84 | 0.001 | 0.009 | 0.95 |
| rs2282978 | | 7 | 92264410 | C | 0.021 | 0.004 | 1.58E-06 | 0.014 | 0.008 | 0.08 | 0.013 | 0.009 | 0.14 | 0.002 | 0.009 | 0.86 | 0.016 | 0.009 | 0.08 | -0.002 | 0.008 | 0.86 |
| rs13231367 | | 7 | 127509070 | G | 0.009 | 0.005 | 4.68E-02 | -0.01 | 0.008 | 0.26 | 0.026 | 0.01 | 0.01 | 0.019 | 0.01 | 0.06 | 0.02 | 0.01 | 0.05 | 0.02 | 0.01 | 0.05 |
| rs6467157 | | 7 | 127660763 | T | 0.014 | 0.005 | 2.98E-03 | -0.008 | 0.008 | 0.35 | 0.009 | 0.009 | 0.29 | 0.009 | 0.009 | 0.3 | 0.009 | 0.009 | 0.32 | 0 | 0.008 | 0.99 |
| rs732563 | | 8 | 23345526 | C | 0.019 | 0.004 | 6.05E-06 | 0.005 | 0.007 | 0.55 | -0.019 | 0.011 | 0.09 | 0.001 | 0.01 | 0.96 | -0.007 | 0.011 | 0.55 | -0.028 | 0.011 | 0.01 |
| rs34036147 | | 8 | 38366249 | T | 0.019 | 0.004 | 1.60E-05 | -0.011 | 0.008 | 0.22 | -0.009 | 0.009 | 0.34 | -0.004 | 0.009 | 0.68 | -0.013 | 0.009 | 0.16 | 0.005 | 0.009 | 0.57 |
| rs13266210 | | 8 | 41533514 | A | 0.03 | 0.005 | 3.05E-09 | 0.01 | 0.009 | 0.26 | 0.007 | 0.011 | 0.5 | 0.024 | 0.011 | 0.04 | -0.004 | 0.011 | 0.72 | 0.024 | 0.011 | 0.03 |
| rs72656010 | | 8 | 57122215 | T | 0.026 | 0.006 | 1.60E-05 | -0.006 | 0.011 | 0.59 | 0.035 | 0.013 | 0.01 | 0.038 | 0.013 | 0 | 0.02 | 0.013 | 0.12 | 0.033 | 0.013 | 0.01 |
| rs6995390 | | 8 | 77611012 | T | -0.014 | 0.006 | 1.02E-02 | -0.016 | 0.01 | 0.11 | -0.005 | 0.008 | 0.58 | -0.011 | 0.008 | 0.17 | -0.007 | 0.008 | 0.39 | 0.007 | 0.008 | 0.42 |
| rs7819593 | | 8 | 106115172 | C | 0.023 | 0.005 | 2.10E-06 | 0.007 | 0.009 | 0.42 | -0.003 | 0.009 | 0.77 | -0.004 | 0.01 | 0.66 | -0.004 | 0.009 | 0.68 | -0.001 | 0.009 | 0.88 |
| rs9657468 | | 8 | 142362391 | G | 0.018 | 0.004 | 3.60E-05 | -0.01 | 0.008 | 0.23 | -0.012 | 0.009 | 0.16 | 0.013 | 0.009 | 0.14 | -0.016 | 0.009 | 0.07 | 0.001 | 0.008 | 0.92 |
| rs28457693 | | 9 | 98217348 | G | 0.04 | 0.007 | 1.70E-09 | -0.021 | 0.012 | 0.09 | 0.024 | 0.009 | 0.01 | 0.011 | 0.009 | 0.21 | 0.022 | 0.009 | 0.02 | 0.014 | 0.009 | 0.11 |
| rs1411424 | | 9 | 113892963 | A | 0.012 | 0.004 | 3.92E-03 | 0.002 | 0.007 | 0.77 | -0.007 | 0.008 | 0.39 | 0.003 | 0.008 | 0.74 | -0.005 | 0.008 | 0.54 | -0.007 | 0.008 | 0.4 |
| rs2418135 | | 9 | 113901309 | A | 0.012 | 0.004 | 2.91E-03 | 0.001 | 0.007 | 0.86 | -0.007 | 0.008 | 0.38 | 0.004 | 0.008 | 0.63 | -0.004 | 0.008 | 0.6 | -0.009 | 0.008 | 0.29 |
| rs72760655 | | 9 | 116916214 | C | -0.009 | 0.004 | 4.42E-02 | -0.029 | 0.008 | 0 | 0.007 | 0.011 | 0.51 | -0.008 | 0.011 | 0.48 | -0.002 | 0.01 | 0.89 | 0.019 | 0.011 | 0.1 |
| rs1323438 | | 9 | 119115531 | C | 0.02 | 0.005 | 1.30E-05 | -0.001 | 0.008 | 0.9 | -0.004 | 0.01 | 0.72 | 0.009 | 0.01 | 0.37 | -0.002 | 0.009 | 0.88 | -0.007 | 0.01 | 0.49 |
| rs3933326 | | 9 | 123633948 | G | 0.023 | 0.004 | 2.16E-07 | 0.014 | 0.008 | 0.11 | 0.002 | 0.008 | 0.84 | -0.018 | 0.009 | 0.04 | 0.006 | 0.009 | 0.49 | -0.006 | 0.009 | 0.49 |
| rs28505901 | | 9 | 139241030 | A | 0.024 | 0.005 | 4.17E-07 | -0.019 | 0.009 | 0.05 | -0.01 | 0.009 | 0.23 | -0.003 | 0.008 | 0.74 | -0.005 | 0.008 | 0.53 | -0.011 | 0.009 | 0.21 |
| rs5030938 | | 10 | 70975916 | T | 0.019 | 0.004 | 1.60E-05 | 0.007 | 0.008 | 0.39 | 0.021 | 0.01 | 0.03 | 0.012 | 0.01 | 0.23 | 0.02 | 0.01 | 0.04 | 0.011 | 0.01 | 0.27 |
| rs9645500 | | 10 | 70986723 | G | 0.019 | 0.004 | 1.00E-05 | 0.004 | 0.008 | 0.65 | 0.004 | 0.008 | 0.62 | -0.015 | 0.008 | 0.08 | 0 | 0.008 | 0.97 | 0.013 | 0.008 | 0.13 |
| rs1112718 | | 10 | 94479107 | G | 0.036 | 0.004 | 1.51E-17 | 0.015 | 0.008 | 0.06 | -0.007 | 0.009 | 0.45 | -0.002 | 0.009 | 0.82 | 0.008 | 0.009 | 0.38 | -0.03 | 0.009 | 0 |
| rs10509669 | | 10 | 95969913 | A | -0.02 | 0.005 | 2.60E-05 | -0.017 | 0.009 | 0.06 | -0.004 | 0.01 | 0.71 | -0.03 | 0.01 | 0 | 0.002 | 0.009 | 0.87 | -0.005 | 0.01 | 0.63 |
| rs562974282 | | 10 | 104201070 | G | -0.126 | 0.054 | 1.90E-02 | - | - | - | - | - | - | - | - | - | - | - | - | - | - | - |
| rs7076938 | | 10 | 115789375 | T | 0.029 | 0.005 | 2.91E-10 | -0.001 | 0.009 | 0.87 | 0.005 | 0.008 | 0.58 | 0 | 0.008 | 0.99 | -0.002 | 0.008 | 0.85 | 0.016 | 0.008 | 0.05 |
| rs71486610 | | 10 | 124134803 | C | 0.016 | 0.004 | 1.70E-04 | -0.037 | 0.008 | 0 | -0.011 | 0.009 | 0.22 | -0.004 | 0.009 | 0.64 | 0.006 | 0.009 | 0.53 | -0.035 | 0.009 | 0 |
| rs11042596 | | 11 | 2118860 | T | 0.027 | 0.004 | 1.61E-09 | -0.014 | 0.008 | 0.09 | 0.006 | 0.009 | 0.53 | -0.021 | 0.009 | 0.02 | 0.009 | 0.009 | 0.31 | -0.003 | 0.009 | 0.74 |
| rs234864 | | 11 | 2857297 | A | 0.017 | 0.004 | 4.90E-05 | 0.012 | 0.008 | 0.12 | - | - | - | - | - | - | - | - | - | - | - | - |
| rs2168101 | | 11 | 8255408 | C | -0.015 | 0.005 | 1.03E-03 | 0.012 | 0.009 | 0.18 | -0.012 | 0.009 | 0.18 | 0 | 0.008 | 0.96 | -0.012 | 0.009 | 0.17 | -0.003 | 0.009 | 0.71 |
| rs4444073 | | 11 | 10331664 | A | 0.023 | 0.004 | 2.20E-08 | 0.006 | 0.008 | 0.46 | -0.004 | 0.008 | 0.62 | -0.006 | 0.008 | 0.5 | 0.004 | 0.008 | 0.67 | -0.011 | 0.008 | 0.17 |
| rs667515 | | 11 | 69449076 | G | 0.013 | 0.004 | 1.51E-03 | 0.003 | 0.008 | 0.71 | -0.03 | 0.009 | 0 | -0.01 | 0.009 | 0.24 | -0.03 | 0.009 | 0 | -0.008 | 0.009 | 0.36 |
| rs76895963 | | 12 | 4384844 | G | 0.051 | 0.016 | 1.28E-03 | -0.102 | 0.03 | 0 | -0.006 | 0.009 | 0.51 | 0.001 | 0.009 | 0.92 | -0.007 | 0.009 | 0.47 | -0.006 | 0.009 | 0.55 |
| rs11055030 | | 12 | 12878349 | G | 0.022 | 0.005 | 9.98E-07 | 0.005 | 0.008 | 0.53 | 0.019 | 0.008 | 0.02 | 0.016 | 0.009 | 0.06 | 0.024 | 0.009 | 0.01 | -0.002 | 0.008 | 0.81 |
| rs8756 | | 12 | 66359752 | C | 0.037 | 0.004 | 1.71E-19 | 0.008 | 0.007 | 0.31 | -0.01 | 0.008 | 0.22 | 0.006 | 0.008 | 0.48 | -0.005 | 0.008 | 0.54 | -0.015 | 0.008 | 0.07 |
| rs7968682 | | 12 | 66371880 | G | 0.037 | 0.004 | 4.87E-20 | 0.008 | 0.008 | 0.31 | 0.114 | 0.033 | 0 | 0.031 | 0.033 | 0.35 | 0.073 | 0.033 | 0.03 | 0.11 | 0.033 | 0 |
| rs1480470 | | 12 | 66412130 | G | 0.028 | 0.004 | 1.07E-10 | -0.001 | 0.008 | 0.91 | 0.01 | 0.008 | 0.22 | -0.003 | 0.008 | 0.74 | 0.01 | 0.008 | 0.23 | 0.006 | 0.008 | 0.45 |
| rs2647873 | | 12 | 103081192 | A | 0.009 | 0.004 | 3.34E-02 | -0.005 | 0.007 | 0.51 | 0.01 | 0.008 | 0.23 | -0.003 | 0.008 | 0.74 | 0.01 | 0.008 | 0.24 | 0.006 | 0.008 | 0.49 |
| rs34217484 | | 13 | 48854550 | A | 0.012 | 0.005 | 1.14E-02 | 0.007 | 0.009 | 0.4 | 0.01 | 0.009 | 0.24 | -0.006 | 0.009 | 0.54 | 0.014 | 0.009 | 0.12 | -0.002 | 0.008 | 0.85 |
| rs72681869 | | 14 | 50655357 | C | 0.108 | 0.021 | 2.73E-07 | -0.05 | 0.048 | 0.31 | 0.006 | 0.011 | 0.62 | 0.014 | 0.012 | 0.22 | 0.001 | 0.011 | 0.96 | 0.007 | 0.011 | 0.57 |
| rs6575803 | | 14 | 101257755 | C | 0.034 | 0.007 | 9.89E-07 | 0.01 | 0.013 | 0.46 | 0.013 | 0.036 | 0.72 | 0.029 | 0.037 | 0.45 | 0.032 | 0.037 | 0.39 | -0.026 | 0.037 | 0.5 |
| rs75844534 | | 15 | 38667117 | A | 0.036 | 0.006 | 1.54E-08 | -0.003 | 0.012 | 0.83 | 0.012 | 0.009 | 0.16 | -0.001 | 0.008 | 0.89 | 0.014 | 0.009 | 0.1 | 0.002 | 0.008 | 0.77 |
| rs339969 | | 15 | 60883281 | A | 0.011 | 0.004 | 1.23E-02 | -0.007 | 0.008 | 0.4 | -0.01 | 0.009 | 0.26 | -0.017 | 0.009 | 0.06 | -0.006 | 0.009 | 0.48 | -0.007 | 0.009 | 0.4 |
| rs3784789 | | 15 | 75082552 | G | -0.018 | 0.004 | 2.40E-05 | -0.007 | 0.008 | 0.4 | -0.005 | 0.009 | 0.57 | -0.019 | 0.009 | 0.03 | 0 | 0.008 | 0.98 | -0.006 | 0.009 | 0.49 |
| rs4932373 | | 15 | 91429287 | A | 0.01 | 0.004 | 2.07E-02 | 0.001 | 0.009 | 0.87 | 0.007 | 0.008 | 0.43 | 0.014 | 0.008 | 0.11 | 0.001 | 0.008 | 0.88 | 0.007 | 0.008 | 0.38 |
| rs7402983 | | 15 | 99193276 | A | 0.027 | 0.004 | 4.61E-10 | -0.003 | 0.011 | 0.77 | 0.007 | 0.012 | 0.57 | -0.005 | 0.012 | 0.71 | 0.017 | 0.012 | 0.18 | -0.017 | 0.012 | 0.19 |
| rs40434 | | 16 | 55699525 | G | 0.017 | 0.004 | 4.80E-05 | -0.002 | 0.008 | 0.76 | -0.006 | 0.008 | 0.47 | 0.001 | 0.008 | 0.94 | 0 | 0.008 | 0.98 | -0.014 | 0.008 | 0.08 |
| rs222857 | | 17 | 7164563 | T | 0.026 | 0.004 | 5.77E-10 | -0.007 | 0.008 | 0.41 | -0.002 | 0.008 | 0.79 | -0.008 | 0.008 | 0.35 | -0.002 | 0.008 | 0.8 | 0.007 | 0.008 | 0.39 |
| rs4511593 | | 17 | 7455536 | T | 0.019 | 0.004 | 7.39E-06 | 0.001 | 0.008 | 0.93 | 0.003 | 0.008 | 0.7 | -0.007 | 0.009 | 0.43 | -0.006 | 0.008 | 0.52 | 0.024 | 0.009 | 0.01 |
| rs9909342 | | 17 | 25652275 | A | 0.019 | 0.004 | 6.71E-06 | 0.003 | 0.008 | 0.71 | -0.01 | 0.008 | 0.23 | 0.001 | 0.008 | 0.88 | -0.003 | 0.008 | 0.72 | -0.016 | 0.008 | 0.05 |
| rs11867479 | | 17 | 68090207 | T | 0.018 | 0.004 | 2.20E-05 | -0.01 | 0.008 | 0.23 | 0.005 | 0.008 | 0.59 | 0.019 | 0.009 | 0.03 | 0.002 | 0.008 | 0.84 | 0.004 | 0.008 | 0.66 |
| rs10221267 | | 17 | 68464662 | T | 0.018 | 0.004 | 1.90E-05 | -0.022 | 0.008 | 0 | -0.027 | 0.027 | 0.33 | 0.029 | 0.028 | 0.3 | -0.047 | 0.028 | 0.09 | 0.008 | 0.026 | 0.77 |
| rs73354194 | | 17 | 79905947 | C | 0.06 | 0.014 | 1.70E-05 | -0.019 | 0.026 | 0.48 | 0.015 | 0.008 | 0.07 | -0.008 | 0.008 | 0.33 | 0.01 | 0.008 | 0.25 | 0.018 | 0.009 | 0.03 |
| rs8106042 | | 19 | 7161849 | G | 0.023 | 0.005 | 6.58E-07 | 0.009 | 0.009 | 0.32 | -0.013 | 0.009 | 0.15 | -0.008 | 0.009 | 0.41 | -0.007 | 0.009 | 0.46 | -0.018 | 0.009 | 0.06 |
| rs41355649 | | 19 | 33790556 | G | 0.042 | 0.008 | 4.52E-07 | -0.011 | 0.016 | 0.49 | 0.012 | 0.016 | 0.49 | 0.029 | 0.017 | 0.09 | 0.018 | 0.017 | 0.29 | -0.017 | 0.017 | 0.31 |
| rs1129156 | | 19 | 40719076 | T | 0.022 | 0.005 | 1.86E-06 | -0.015 | 0.008 | 0.08 | 0.008 | 0.009 | 0.4 | 0.01 | 0.009 | 0.29 | 0.004 | 0.009 | 0.68 | 0.01 | 0.009 | 0.27 |
| rs147957154 | | 19 | 43431040 | T | 0.026 | 0.006 | 2.40E-05 | 0.001 | 0.011 | 0.9 | - | - | - | - | - | - | - | - | - | - | - | - |
| rs1203876 | | 20 | 22540915 | C | 0.055 | 0.01 | 1.28E-08 | -0.007 | 0.017 | 0.7 | 0.005 | 0.008 | 0.56 | -0.011 | 0.009 | 0.19 | 0.008 | 0.008 | 0.33 | 0 | 0.008 | 0.97 |
| rs11698914 | | 20 | 31327144 | C | 0.029 | 0.005 | 2.75E-09 | -0.002 | 0.009 | 0.81 | 0.018 | 0.01 | 0.06 | 0 | 0.009 | 0.97 | 0.017 | 0.01 | 0.09 | 0.011 | 0.01 | 0.28 |
| rs1012167 | | 20 | 39159119 | C | 0.024 | 0.004 | 1.86E-08 | -0.009 | 0.008 | 0.24 | 0.02 | 0.022 | 0.37 | -0.005 | 0.021 | 0.82 | 0.006 | 0.021 | 0.78 | 0.05 | 0.022 | 0.02 |
| rs753381 | | 20 | 39797465 | T | 0.018 | 0.004 | 9.05E-06 | -0.003 | 0.007 | 0.69 | -0.004 | 0.008 | 0.66 | -0.013 | 0.008 | 0.11 | 0 | 0.008 | 0.98 | -0.004 | 0.008 | 0.6 |
| rs6026449 | | 20 | 57272617 | C | 0.018 | 0.004 | 3.20E-05 | 0 | 0.008 | 0.99 | -0.002 | 0.011 | 0.88 | -0.004 | 0.011 | 0.73 | 0.003 | 0.011 | 0.8 | -0.011 | 0.012 | 0.35 |
| rs73143584 | | 20 | 62445702 | A | 0.031 | 0.007 | 3.28E-06 | 0.001 | 0.012 | 0.91 | 0.014 | 0.008 | 0.08 | 0.011 | 0.008 | 0.21 | 0.011 | 0.008 | 0.17 | 0.008 | 0.008 | 0.31 |
| rs134594 | | 22 | 29468456 | C | 0.022 | 0.004 | 6.02E-07 | 0.002 | 0.01 | 0.82 | 0 | 0.008 | 0.96 | 0.012 | 0.009 | 0.16 | 0.001 | 0.008 | 0.91 | -0.006 | 0.008 | 0.52 |
| rs41311445 | | 22 | 42070374 | A | 0.034 | 0.007 | 1.31E-06 | -0.009 | 0.015 | 0.57 | 0.013 | 0.013 | 0.31 | 0.011 | 0.013 | 0.4 | 0.007 | 0.013 | 0.61 | 0.012 | 0.013 | 0.36 |
| rs7285579 | | 22 | 46441980 | C | 0.018 | 0.005 | 1.06E-04 | -0.013 | 0.009 | 0.18 | 0.028 | 0.009 | 0 | 0.016 | 0.009 | 0.09 | 0.018 | 0.009 | 0.05 | 0.023 | 0.009 | 0.01 |

**Table S7.** Summary statistics for the 205 SNPs used in instrumental variable analysis to assess the effect of fetal birth weight on the hip bone parameters.

| **205 instrument varibles** | | | | | **Fetal birth weight (Warrington et al. 2019)** | | | **Femoral neck BMD (Zheng. 2015)** | | | **total hip area (Styrkarsdottir et al. 2019)** | | | **Femoral neck area (Styrkarsdottir et al. 2019)** | | | **Intertrochanteric area (Styrkarsdottir et al. 2019)** | | | **Trochanter area (Styrkarsdottir et al. 2019)** | | |
| --- | --- | --- | --- | --- | --- | --- | --- | --- | --- | --- | --- | --- | --- | --- | --- | --- | --- | --- | --- | --- | --- | --- |
| **SNP** | **CHR** | | **BP** | **EA** | **β** | **SE** | ***P*** | **β** | **SE** | ***P*** | **β** | **SE** | ***P*** | **β** | **SE** | ***P*** | **β** | **SE** | ***P*** | **β** | **SE** | ***P*** |
| rs17367504 | | 1 | 11862778 | G | 0.012 | 0.003 | 8.33E-04 | 0.014 | 0.01 | 0.17 | -0.004 | 0.011 | 0.76 | 0.009 | 0.011 | 0.45 | -0.009 | 0.011 | 0.43 | 0.005 | 0.011 | 0.69 |
| rs12401656 | | 1 | 43456767 | G | 0.025 | 0.004 | 3.36E-11 | -0.017 | 0.011 | 0.13 | -0.013 | 0.012 | 0.27 | -0.027 | 0.012 | 0.03 | -0.01 | 0.012 | 0.41 | -0.01 | 0.012 | 0.43 |
| rs80278614 | | 1 | 119412317 | A | 0.04 | 0.006 | 6.45E-12 | -0.024 | 0.017 | 0.17 | 0.017 | 0.016 | 0.31 | -0.05 | 0.017 | 0 | 0.031 | 0.017 | 0.07 | -0.008 | 0.016 | 0.63 |
| rs905938 | | 1 | 154991389 | C | 0.026 | 0.003 | 2.77E-19 | 0.003 | 0.009 | 0.73 | 0.021 | 0.009 | 0.02 | -0.002 | 0.009 | 0.79 | 0.016 | 0.009 | 0.07 | 0.019 | 0.009 | 0.03 |
| rs670523 | | 1 | 155878732 | G | 0.019 | 0.003 | 7.58E-12 | 0.009 | 0.009 | 0.29 | -0.011 | 0.009 | 0.21 | -0.011 | 0.009 | 0.23 | -0.011 | 0.009 | 0.2 | 0.001 | 0.008 | 0.91 |
| rs72480273 | | 1 | 161644871 | C | 0.023 | 0.003 | 3.95E-11 | 0.003 | 0.01 | 0.79 | -0.003 | 0.009 | 0.76 | 0.013 | 0.01 | 0.19 | -0.009 | 0.01 | 0.34 | 0.005 | 0.01 | 0.65 |
| rs10913200 | | 1 | 176521655 | G | 0.051 | 0.008 | 2.02E-10 | -0.007 | 0.024 | 0.78 | 0.059 | 0.032 | 0.06 | -0.007 | 0.03 | 0.83 | 0.024 | 0.031 | 0.45 | 0.082 | 0.032 | 0.01 |
| rs61830764 | | 1 | 212289976 | A | 0.017 | 0.003 | 1.12E-09 | -0.011 | 0.008 | 0.17 | -0.006 | 0.008 | 0.46 | -0.012 | 0.009 | 0.16 | 0 | 0.008 | 0.97 | -0.01 | 0.008 | 0.22 |
| rs3806315 | | 1 | 214724668 | A | 0.018 | 0.003 | 2.78E-11 | 0.001 | 0.01 | 0.92 | 0.008 | 0.008 | 0.33 | 0.016 | 0.009 | 0.07 | -0.001 | 0.008 | 0.88 | 0.017 | 0.009 | 0.05 |
| rs708122 | | 1 | 228216997 | C | 0.017 | 0.003 | 2.45E-09 | -0.016 | 0.008 | 0.05 | 0.01 | 0.009 | 0.25 | -0.003 | 0.009 | 0.77 | 0.011 | 0.009 | 0.21 | -0.001 | 0.008 | 0.95 |
| rs10495563 | | 2 | 9662210 | A | 0.022 | 0.003 | 2.06E-16 | -0.018 | 0.008 | 0.03 | 0.016 | 0.009 | 0.06 | 0.018 | 0.009 | 0.04 | 0.007 | 0.009 | 0.4 | 0.018 | 0.009 | 0.04 |
| rs11893688 | | 2 | 9695282 | T | 0.022 | 0.003 | 1.29E-15 | -0.016 | 0.008 | 0.05 | 0.015 | 0.009 | 0.09 | 0.015 | 0.009 | 0.1 | 0.005 | 0.009 | 0.57 | 0.02 | 0.009 | 0.03 |
| rs2551347 | | 2 | 23912401 | T | 0.024 | 0.003 | 1.93E-16 | -0.014 | 0.009 | 0.11 | 0.002 | 0.009 | 0.83 | -0.002 | 0.009 | 0.83 | -0.005 | 0.009 | 0.62 | 0.012 | 0.01 | 0.22 |
| rs1179494 | | 2 | 36809496 | G | 0.01 | 0.003 | 1.50E-04 | -0.013 | 0.008 | 0.12 | -0.007 | 0.008 | 0.42 | 0.016 | 0.009 | 0.06 | -0.008 | 0.009 | 0.36 | -0.005 | 0.008 | 0.55 |
| rs754868 | | 2 | 43185532 | G | 0.016 | 0.003 | 6.73E-10 | 0.007 | 0.008 | 0.39 | -0.002 | 0.008 | 0.82 | 0.012 | 0.008 | 0.16 | 0.001 | 0.008 | 0.92 | -0.008 | 0.008 | 0.33 |
| rs4952673 | | 2 | 43423870 | A | 0.007 | 0.003 | 3.83E-03 | -0.004 | 0.008 | 0.65 | 0.008 | 0.008 | 0.34 | 0.011 | 0.008 | 0.17 | 0.015 | 0.008 | 0.06 | -0.016 | 0.008 | 0.05 |
| rs17034876 | | 2 | 46484310 | T | 0.042 | 0.003 | 3.11E-47 | 0.01 | 0.008 | 0.25 | -0.011 | 0.009 | 0.23 | 0.02 | 0.009 | 0.03 | -0.02 | 0.009 | 0.03 | 0.009 | 0.009 | 0.32 |
| rs4953353 | | 2 | 46567276 | G | 0.018 | 0.003 | 3.45E-11 | 0.01 | 0.008 | 0.21 | -0.002 | 0.008 | 0.84 | -0.001 | 0.008 | 0.94 | -0.001 | 0.008 | 0.9 | -0.002 | 0.008 | 0.79 |
| rs186606513 | | 2 | 97482001 | G | 0.061 | 0.01 | 2.68E-09 | -0.066 | 0.037 | 0.08 | 0.001 | 0.03 | 0.97 | -0.055 | 0.034 | 0.1 | 0.006 | 0.031 | 0.86 | 0.012 | 0.032 | 0.73 |
| rs56188432 | | 2 | 158406865 | G | 0.258 | 0.031 | 1.38E-16 | - | - | - | 0.19 | 0.063 | 0 | 0.073 | 0.064 | 0.26 | 0.187 | 0.064 | 0 | 0.088 | 0.064 | 0.17 |
| rs560887 | | 2 | 169763148 | C | -0.008 | 0.003 | 5.78E-03 | 0.001 | 0.008 | 0.87 | -0.01 | 0.009 | 0.27 | 0 | 0.008 | 0.97 | -0.011 | 0.009 | 0.24 | -0.003 | 0.009 | 0.74 |
| rs2280235 | | 2 | 191843830 | G | 0.018 | 0.003 | 6.90E-10 | -0.019 | 0.009 | 0.03 | 0 | 0.009 | 0.98 | 0.005 | 0.009 | 0.6 | -0.003 | 0.009 | 0.74 | 0.005 | 0.009 | 0.59 |
| rs10181515 | | 2 | 227019461 | T | 0.021 | 0.003 | 2.13E-12 | 0.023 | 0.009 | 0.01 | 0.004 | 0.009 | 0.66 | -0.013 | 0.01 | 0.19 | 0.002 | 0.009 | 0.83 | 0.007 | 0.009 | 0.46 |
| rs9855896 | | 3 | 14287150 | G | 0.004 | 0.003 | 1.86E-01 | 0.018 | 0.009 | 0.06 | 0.002 | 0.009 | 0.84 | 0 | 0.009 | 0.97 | 0.006 | 0.01 | 0.52 | -0.004 | 0.009 | 0.71 |
| rs2168443 | | 3 | 46947087 | T | 0.017 | 0.003 | 3.89E-10 | 0.003 | 0.008 | 0.7 | -0.023 | 0.009 | 0.01 | 0.001 | 0.008 | 0.94 | -0.027 | 0.009 | 0 | -0.005 | 0.008 | 0.55 |
| rs11708067 | | 3 | 123065778 | G | 0.041 | 0.003 | 1.59E-42 | -0.012 | 0.009 | 0.19 | 0.008 | 0.01 | 0.44 | 0.004 | 0.01 | 0.67 | 0.008 | 0.01 | 0.44 | -0.001 | 0.009 | 0.89 |
| rs9851257 | | 3 | 123125711 | T | 0.02 | 0.003 | 2.36E-12 | 0.009 | 0.008 | 0.29 | 0 | 0.008 | 0.97 | -0.007 | 0.009 | 0.44 | -0.003 | 0.009 | 0.78 | 0.012 | 0.009 | 0.2 |
| rs6440006 | | 3 | 141142691 | A | 0.01 | 0.003 | 7.32E-05 | -0.009 | 0.008 | 0.24 | -0.008 | 0.008 | 0.32 | -0.004 | 0.008 | 0.6 | -0.014 | 0.008 | 0.09 | 0.008 | 0.008 | 0.34 |
| rs2306700 | | 3 | 142123841 | T | 0.023 | 0.004 | 1.81E-09 | 0.024 | 0.011 | 0.03 | 0.024 | 0.013 | 0.06 | -0.002 | 0.012 | 0.86 | 0.02 | 0.013 | 0.11 | 0.016 | 0.013 | 0.22 |
| rs10935733 | | 3 | 148622968 | T | 0.019 | 0.003 | 2.33E-13 | 0.019 | 0.008 | 0.03 | -0.011 | 0.008 | 0.19 | -0.005 | 0.008 | 0.57 | -0.015 | 0.008 | 0.07 | 0.002 | 0.008 | 0.78 |
| rs4679760 | | 3 | 155855418 | G | 0.009 | 0.003 | 3.18E-04 | -0.003 | 0.008 | 0.68 | 0.009 | 0.008 | 0.3 | -0.004 | 0.008 | 0.65 | 0.011 | 0.008 | 0.2 | 0.005 | 0.008 | 0.55 |
| rs1482852 | | 3 | 156798294 | A | 0.05 | 0.003 | 1.58E-82 | -0.019 | 0.008 | 0.02 | 0.026 | 0.009 | 0 | 0.026 | 0.009 | 0 | 0.02 | 0.009 | 0.02 | 0.015 | 0.009 | 0.09 |
| rs11711420 | | 3 | 183349010 | T | 0.019 | 0.003 | 3.22E-10 | -0.01 | 0.009 | 0.26 | 0.002 | 0.009 | 0.8 | -0.015 | 0.01 | 0.14 | 0.007 | 0.01 | 0.46 | -0.008 | 0.01 | 0.41 |
| rs4144829 | | 4 | 17903654 | C | 0.036 | 0.003 | 4.32E-34 | 0.009 | 0.009 | 0.33 | 0.028 | 0.009 | 0 | 0.032 | 0.009 | 0 | 0.018 | 0.009 | 0.05 | 0.025 | 0.009 | 0.01 |
| rs2174633 | | 4 | 17917781 | A | 0.035 | 0.003 | 7.05E-33 | 0.009 | 0.009 | 0.29 | 0.029 | 0.009 | 0 | 0.031 | 0.009 | 0 | 0.019 | 0.009 | 0.03 | 0.024 | 0.009 | 0.01 |
| rs2189234 | | 4 | 106075498 | G | 0.015 | 0.003 | 1.21E-08 | -0.002 | 0.008 | 0.84 | 0.005 | 0.008 | 0.56 | -0.011 | 0.009 | 0.19 | 0.003 | 0.008 | 0.71 | 0.006 | 0.008 | 0.46 |
| rs6533183 | | 4 | 106133184 | C | 0.022 | 0.003 | 6.78E-16 | 0.018 | 0.008 | 0.02 | -0.003 | 0.008 | 0.76 | -0.011 | 0.009 | 0.23 | -0.007 | 0.009 | 0.43 | 0.008 | 0.009 | 0.35 |
| rs116807401 | | 4 | 135121721 | C | 0.077 | 0.01 | 2.23E-13 | 0.035 | 0.031 | 0.27 | 0.069 | 0.038 | 0.07 | 0.024 | 0.037 | 0.54 | 0.048 | 0.038 | 0.2 | 0.069 | 0.038 | 0.07 |
| rs6845999 | | 4 | 145565826 | T | 0.026 | 0.003 | 1.50E-24 | -0.01 | 0.008 | 0.19 | 0.033 | 0.008 | 0 | 0.059 | 0.008 | 0 | 0.019 | 0.008 | 0.02 | 0.023 | 0.008 | 0 |
| rs2131354 | | 4 | 145599908 | A | 0.026 | 0.003 | 3.49E-24 | -0.01 | 0.008 | 0.18 | 0.038 | 0.008 | 0 | 0.06 | 0.008 | 0 | 0.026 | 0.008 | 0 | 0.021 | 0.008 | 0.01 |
| rs4579095 | | 4 | 174726635 | G | 0.003 | 0.003 | 3.19E-01 | -0.015 | 0.008 | 0.05 | -0.013 | 0.009 | 0.14 | -0.003 | 0.009 | 0.71 | -0.013 | 0.009 | 0.15 | -0.006 | 0.009 | 0.48 |
| rs1818782 | | 5 | 39424628 | C | 0.016 | 0.003 | 4.17E-09 | 0.005 | 0.008 | 0.55 | -0.021 | 0.009 | 0.01 | -0.019 | 0.009 | 0.03 | -0.024 | 0.009 | 0.01 | 0.002 | 0.008 | 0.83 |
| rs351930 | | 5 | 52003397 | T | 0.019 | 0.003 | 2.85E-09 | -0.016 | 0.009 | 0.08 | 0.007 | 0.01 | 0.47 | 0.033 | 0.01 | 0 | -0.001 | 0.009 | 0.95 | 0.007 | 0.01 | 0.51 |
| rs854037 | | 5 | 57091783 | A | 0.027 | 0.003 | 9.41E-16 | 0.006 | 0.01 | 0.55 | -0.01 | 0.01 | 0.3 | -0.011 | 0.01 | 0.28 | -0.003 | 0.01 | 0.74 | -0.018 | 0.01 | 0.08 |
| rs28365970 | | 5 | 67585723 | C | 0.02 | 0.003 | 1.65E-11 | -0.013 | 0.009 | 0.12 | 0.025 | 0.009 | 0.01 | 0.019 | 0.009 | 0.05 | 0.011 | 0.009 | 0.22 | 0.032 | 0.009 | 0 |
| rs6871635 | | 5 | 133830395 | G | 0.016 | 0.003 | 3.02E-09 | -0.002 | 0.008 | 0.85 | 0 | 0.008 | 0.97 | 0.009 | 0.008 | 0.31 | 0.006 | 0.008 | 0.49 | -0.014 | 0.008 | 0.09 |
| rs1981627 | | 5 | 133838180 | G | 0.017 | 0.003 | 8.36E-11 | -0.001 | 0.008 | 0.88 | 0.001 | 0.008 | 0.89 | 0.008 | 0.008 | 0.32 | 0.006 | 0.008 | 0.49 | -0.013 | 0.008 | 0.13 |
| rs2946179 | | 5 | 157886627 | C | 0.02 | 0.003 | 1.14E-11 | -0.013 | 0.008 | 0.14 | -0.005 | 0.009 | 0.59 | 0.006 | 0.009 | 0.5 | -0.002 | 0.009 | 0.81 | -0.01 | 0.009 | 0.31 |
| rs34471628 | | 5 | 172196752 | A | 0.018 | 0.007 | 9.41E-03 | 0.043 | 0.019 | 0.03 | -0.033 | 0.019 | 0.08 | -0.015 | 0.019 | 0.42 | -0.039 | 0.019 | 0.04 | -0.001 | 0.017 | 0.96 |
| rs9379084 | | 6 | 7231843 | G | 0.022 | 0.004 | 1.22E-07 | 0.008 | 0.011 | 0.47 | -0.011 | 0.014 | 0.44 | -0.024 | 0.014 | 0.09 | -0.029 | 0.014 | 0.03 | 0.042 | 0.014 | 0 |
| rs35261542 | | 6 | 20675792 | C | 0.041 | 0.003 | 2.77E-45 | -0.006 | 0.009 | 0.5 | -0.013 | 0.01 | 0.18 | -0.012 | 0.01 | 0.21 | -0.01 | 0.01 | 0.33 | -0.004 | 0.009 | 0.67 |
| rs9379832 | | 6 | 26186200 | A | 0.022 | 0.003 | 1.06E-13 | -0.007 | 0.009 | 0.42 | - | - | - | - | - | - | - | - | - | - | - | - |
| rs9366778 | | 6 | 31269173 | G | 0.018 | 0.003 | 2.86E-11 | -0.003 | 0.008 | 0.71 | 0.018 | 0.008 | 0.03 | 0.021 | 0.009 | 0.01 | 0.017 | 0.008 | 0.04 | 0.001 | 0.008 | 0.87 |
| rs6911024 | | 6 | 31368451 | T | 0.017 | 0.004 | 1.28E-04 | -0.006 | 0.012 | 0.62 | -0.013 | 0.014 | 0.37 | -0.016 | 0.015 | 0.27 | -0.014 | 0.015 | 0.34 | 0.003 | 0.014 | 0.84 |
| rs9267812 | | 6 | 32128394 | T | 0.023 | 0.004 | 3.11E-09 | -0.024 | 0.014 | 0.09 | -0.026 | 0.012 | 0.03 | -0.006 | 0.012 | 0.61 | -0.028 | 0.012 | 0.02 | -0.002 | 0.011 | 0.89 |
| rs1547669 | | 6 | 33775641 | G | 0.018 | 0.003 | 6.21E-12 | -0.016 | 0.007 | 0.03 | 0 | 0.196 | 1 | -0.017 | 0.008 | 0.05 | -0.001 | 0.008 | 0.88 | 0.007 | 0.008 | 0.43 |
| rs75104038 | | 6 | 34190104 | A | 0.045 | 0.006 | 4.28E-16 | -0.001 | 0.016 | 0.94 | 0.046 | 0.021 | 0.03 | 0.017 | 0.021 | 0.43 | 0.035 | 0.021 | 0.1 | 0.042 | 0.021 | 0.05 |
| rs75034466 | | 6 | 34199815 | T | 0.046 | 0.006 | 1.79E-13 | 0.008 | 0.018 | 0.67 | 0.045 | 0.023 | 0.04 | 0.022 | 0.023 | 0.34 | 0.037 | 0.023 | 0.11 | 0.033 | 0.023 | 0.14 |
| rs6911621 | | 6 | 35529025 | T | 0.018 | 0.003 | 1.64E-11 | -0.008 | 0.008 | 0.32 | 0.022 | 0.008 | 0.01 | 0.018 | 0.009 | 0.04 | 0.014 | 0.008 | 0.1 | 0.017 | 0.008 | 0.05 |
| rs9348981 | | 6 | 35687249 | T | 0.021 | 0.003 | 2.16E-13 | -0.003 | 0.008 | 0.7 | 0.029 | 0.009 | 0 | 0.015 | 0.009 | 0.12 | 0.021 | 0.009 | 0.02 | 0.021 | 0.009 | 0.02 |
| rs7744700 | | 6 | 53349401 | T | 0.02 | 0.003 | 1.64E-11 | 0.008 | 0.009 | 0.39 | -0.003 | 0.008 | 0.7 | 0 | 0.008 | 0.99 | 0 | 0.008 | 0.96 | -0.003 | 0.008 | 0.72 |
| rs76094073 | | 6 | 109288036 | G | 0.027 | 0.004 | 1.63E-11 | -0.006 | 0.011 | 0.6 | 0.017 | 0.012 | 0.17 | 0.009 | 0.012 | 0.45 | 0.022 | 0.012 | 0.07 | -0.011 | 0.012 | 0.36 |
| rs6568554 | | 6 | 109290319 | A | 0.021 | 0.004 | 1.11E-08 | -0.01 | 0.011 | 0.39 | 0.014 | 0.011 | 0.23 | 0.006 | 0.011 | 0.59 | 0.015 | 0.011 | 0.18 | -0.004 | 0.011 | 0.73 |
| rs6925689 | | 6 | 126865884 | T | 0.015 | 0.003 | 6.40E-09 | -0.029 | 0.008 | 0 | 0.002 | 0.008 | 0.8 | 0.004 | 0.008 | 0.61 | 0 | 0.007 | 0.99 | 0.001 | 0.008 | 0.94 |
| rs6569647 | | 6 | 130337266 | T | 0.02 | 0.003 | 6.31E-10 | -0.015 | 0.009 | 0.11 | 0.011 | 0.01 | 0.26 | 0.003 | 0.009 | 0.8 | 0.009 | 0.01 | 0.37 | 0.001 | 0.009 | 0.9 |
| rs1415701 | | 6 | 130345835 | G | 0.018 | 0.003 | 1.35E-09 | -0.008 | 0.009 | 0.35 | 0.01 | 0.009 | 0.28 | 0 | 0.008 | 0.99 | 0.007 | 0.009 | 0.43 | 0.004 | 0.009 | 0.67 |
| rs6930558 | | 6 | 141878920 | T | 0.022 | 0.003 | 3.44E-13 | -0.004 | 0.009 | 0.69 | 0.029 | 0.009 | 0 | 0.028 | 0.01 | 0 | 0.021 | 0.009 | 0.02 | 0.015 | 0.009 | 0.11 |
| rs962554 | | 6 | 142734204 | T | 0.017 | 0.003 | 3.75E-09 | 0.008 | 0.008 | 0.35 | 0.024 | 0.009 | 0.01 | 0.011 | 0.01 | 0.27 | 0.03 | 0.009 | 0 | -0.001 | 0.009 | 0.93 |
| rs10872678 | | 6 | 152039964 | T | 0.032 | 0.003 | 9.78E-29 | -0.015 | 0.009 | 0.08 | -0.005 | 0.009 | 0.6 | -0.008 | 0.009 | 0.36 | -0.006 | 0.009 | 0.51 | 0.005 | 0.009 | 0.59 |
| rs7772579 | | 6 | 152042502 | A | 0.031 | 0.003 | 6.43E-28 | -0.022 | 0.01 | 0.03 | -0.004 | 0.009 | 0.65 | -0.007 | 0.009 | 0.42 | -0.006 | 0.009 | 0.49 | 0.007 | 0.009 | 0.46 |
| rs2934844 | | 6 | 166142456 | T | 0.021 | 0.003 | 1.80E-13 | 0.005 | 0.008 | 0.59 | 0.01 | 0.009 | 0.28 | 0.003 | 0.009 | 0.76 | 0.005 | 0.009 | 0.55 | 0.011 | 0.009 | 0.24 |
| rs1724889 | | 7 | 2741021 | G | 0.016 | 0.003 | 1.45E-07 | -0.023 | 0.009 | 0.01 | 0.01 | 0.009 | 0.3 | -0.001 | 0.009 | 0.88 | 0.011 | 0.009 | 0.26 | 0.005 | 0.009 | 0.58 |
| rs4719648 | | 7 | 2756832 | C | 0.019 | 0.003 | 2.57E-13 | -0.006 | 0.008 | 0.45 | -0.006 | 0.008 | 0.49 | 0.015 | 0.009 | 0.08 | -0.009 | 0.008 | 0.27 | 0.001 | 0.008 | 0.89 |
| rs59084784 | | 7 | 22739562 | A | 0.017 | 0.003 | 2.42E-09 | -0.005 | 0.008 | 0.51 | -0.018 | 0.009 | 0.05 | 0.002 | 0.008 | 0.86 | -0.01 | 0.009 | 0.28 | -0.023 | 0.009 | 0.01 |
| rs7808457 | | 7 | 22798265 | A | 0.01 | 0.003 | 7.10E-05 | 0.005 | 0.008 | 0.51 | -0.016 | 0.008 | 0.05 | -0.007 | 0.008 | 0.37 | -0.014 | 0.008 | 0.1 | -0.008 | 0.008 | 0.33 |
| rs34776209 | | 7 | 23513093 | C | 0.023 | 0.003 | 8.53E-15 | -0.014 | 0.009 | 0.12 | 0.026 | 0.01 | 0.01 | 0.019 | 0.01 | 0.06 | 0.02 | 0.01 | 0.05 | 0.02 | 0.01 | 0.05 |
| rs2908279 | | 7 | 44174857 | T | 0.011 | 0.003 | 2.17E-05 | 0.001 | 0.008 | 0.9 | 0.014 | 0.008 | 0.1 | -0.005 | 0.008 | 0.55 | 0.018 | 0.008 | 0.03 | 0.001 | 0.008 | 0.92 |
| rs2971669 | | 7 | 44231778 | T | 0.011 | 0.003 | 4.17E-04 | 0.012 | 0.01 | 0.26 | -0.018 | 0.011 | 0.1 | -0.014 | 0.011 | 0.21 | -0.017 | 0.011 | 0.11 | -0.005 | 0.011 | 0.66 |
| rs138715366 | | 7 | 44246271 | C | 0.24 | 0.015 | 4.04E-61 | 0.005 | 0.046 | 0.92 | 0.061 | 0.064 | 0.34 | 0.053 | 0.064 | 0.42 | 0.019 | 0.061 | 0.77 | 0.123 | 0.065 | 0.06 |
| rs10265133 | | 7 | 45895604 | G | 0.001 | 0.004 | 8.59E-01 | -0.02 | 0.012 | 0.08 | 0.001 | 0.01 | 0.95 | -0.015 | 0.011 | 0.18 | 0.007 | 0.011 | 0.49 | -0.007 | 0.011 | 0.52 |
| rs11983722 | | 7 | 46298647 | A | 0.032 | 0.005 | 3.10E-09 | 0.027 | 0.015 | 0.09 | -0.014 | 0.015 | 0.34 | -0.011 | 0.015 | 0.48 | 0.002 | 0.014 | 0.9 | -0.042 | 0.015 | 0.01 |
| rs10265057 | | 7 | 47275737 | G | 0.027 | 0.004 | 1.30E-09 | 0.004 | 0.013 | 0.76 | -0.003 | 0.012 | 0.79 | -0.002 | 0.012 | 0.9 | 0.003 | 0.012 | 0.8 | -0.016 | 0.013 | 0.21 |
| rs2237467 | | 7 | 50733316 | A | 0.018 | 0.003 | 5.34E-09 | -0.012 | 0.009 | 0.18 | 0.001 | 0.009 | 0.91 | 0.014 | 0.01 | 0.19 | -0.002 | 0.01 | 0.84 | 0.001 | 0.009 | 0.95 |
| rs112139215 | | 7 | 73034559 | A | 0.047 | 0.005 | 2.81E-20 | 0.015 | 0.015 | 0.32 | -0.029 | 0.016 | 0.06 | -0.017 | 0.016 | 0.28 | -0.025 | 0.016 | 0.11 | -0.018 | 0.016 | 0.26 |
| rs2282978 | | 7 | 92264410 | C | 0.018 | 0.003 | 1.66E-11 | 0.014 | 0.008 | 0.08 | 0.013 | 0.009 | 0.14 | 0.002 | 0.009 | 0.86 | 0.016 | 0.009 | 0.08 | -0.002 | 0.008 | 0.86 |
| rs45446698 | | 7 | 99332948 | G | 0.025 | 0.007 | 1.74E-04 | -0.082 | 0.022 | 0 | 0.002 | 0.016 | 0.93 | -0.005 | 0.017 | 0.76 | -0.015 | 0.017 | 0.38 | 0.035 | 0.017 | 0.04 |
| rs13231367 | | 7 | 127509070 | G | 0.017 | 0.003 | 4.36E-09 | -0.01 | 0.008 | 0.26 | 0.003 | 0.008 | 0.7 | 0.006 | 0.009 | 0.49 | 0.003 | 0.008 | 0.76 | -0.001 | 0.008 | 0.87 |
| rs6467157 | | 7 | 127660763 | T | 0.02 | 0.003 | 1.47E-11 | -0.008 | 0.008 | 0.35 | 0.009 | 0.009 | 0.29 | 0.009 | 0.009 | 0.3 | 0.009 | 0.009 | 0.32 | 0 | 0.008 | 0.99 |
| rs3918226 | | 7 | 150690176 | C | 0.015 | 0.005 | 1.93E-03 | 0 | 0.015 | 0.99 | 0.008 | 0.014 | 0.59 | -0.015 | 0.015 | 0.32 | 0.006 | 0.014 | 0.68 | 0.007 | 0.014 | 0.65 |
| rs62496903 | | 8 | 6446938 | T | 0.033 | 0.005 | 6.74E-12 | -0.028 | 0.014 | 0.05 | 0.023 | 0.014 | 0.11 | 0.006 | 0.014 | 0.69 | 0.027 | 0.014 | 0.06 | -0.002 | 0.013 | 0.9 |
| rs732563 | | 8 | 23345526 | C | 0.017 | 0.003 | 1.29E-11 | 0.005 | 0.007 | 0.55 | -0.005 | 0.008 | 0.58 | -0.011 | 0.008 | 0.17 | -0.007 | 0.008 | 0.39 | 0.007 | 0.008 | 0.42 |
| rs11778247 | | 8 | 23403378 | G | 0.014 | 0.003 | 8.20E-05 | 0.012 | 0.01 | 0.26 | -0.012 | 0.011 | 0.27 | -0.023 | 0.011 | 0.04 | -0.013 | 0.011 | 0.24 | 0.005 | 0.011 | 0.67 |
| rs34036147 | | 8 | 38366249 | T | 0.018 | 0.003 | 8.37E-11 | -0.011 | 0.008 | 0.22 | -0.009 | 0.009 | 0.34 | -0.004 | 0.009 | 0.68 | -0.013 | 0.009 | 0.16 | 0.005 | 0.009 | 0.57 |
| rs13266210 | | 8 | 41533514 | A | 0.027 | 0.003 | 1.54E-17 | 0.01 | 0.009 | 0.26 | -0.019 | 0.011 | 0.09 | 0.001 | 0.01 | 0.96 | -0.007 | 0.011 | 0.55 | -0.028 | 0.011 | 0.01 |
| rs72656010 | | 8 | 57122215 | T | 0.028 | 0.004 | 1.39E-13 | -0.006 | 0.011 | 0.59 | 0.035 | 0.013 | 0.01 | 0.038 | 0.013 | 0 | 0.02 | 0.013 | 0.12 | 0.033 | 0.013 | 0.01 |
| rs6995390 | | 8 | 77611012 | T | 0.006 | 0.003 | 7.85E-02 | -0.016 | 0.01 | 0.11 | 0.007 | 0.011 | 0.5 | 0.024 | 0.011 | 0.04 | -0.004 | 0.011 | 0.72 | 0.024 | 0.011 | 0.03 |
| rs7819593 | | 8 | 106115172 | C | 0.022 | 0.003 | 6.22E-13 | 0.007 | 0.009 | 0.42 | -0.003 | 0.009 | 0.77 | -0.004 | 0.01 | 0.66 | -0.004 | 0.009 | 0.68 | -0.001 | 0.009 | 0.88 |
| rs10283100 | | 8 | 120596023 | G | 0.042 | 0.006 | 7.01E-13 | -0.023 | 0.016 | 0.14 | -0.041 | 0.017 | 0.02 | -0.016 | 0.017 | 0.34 | -0.016 | 0.017 | 0.35 | -0.066 | 0.017 | 0 |
| rs13271368 | | 8 | 126506140 | C | 0.02 | 0.003 | 2.30E-11 | -0.012 | 0.009 | 0.2 | 0.008 | 0.01 | 0.45 | 0.001 | 0.01 | 0.92 | 0.018 | 0.01 | 0.08 | -0.023 | 0.01 | 0.03 |
| rs13257363 | | 8 | 142252580 | G | 0.018 | 0.003 | 2.01E-11 | 0.001 | 0.008 | 0.92 | 0.001 | 0.008 | 0.86 | -0.008 | 0.008 | 0.32 | 0 | 0.008 | 0.97 | 0.007 | 0.008 | 0.4 |
| rs9657468 | | 8 | 142362391 | G | 0.015 | 0.003 | 7.93E-08 | -0.01 | 0.008 | 0.23 | -0.012 | 0.009 | 0.16 | 0.013 | 0.009 | 0.14 | -0.016 | 0.009 | 0.07 | 0.001 | 0.008 | 0.92 |
| rs7854962 | | 9 | 96900505 | C | 0.022 | 0.003 | 1.02E-11 | 0.011 | 0.009 | 0.25 | 0.003 | 0.009 | 0.78 | -0.022 | 0.01 | 0.03 | 0.004 | 0.01 | 0.7 | 0.007 | 0.01 | 0.5 |
| rs28457693 | | 9 | 98217348 | G | 0.044 | 0.004 | 9.89E-26 | -0.021 | 0.012 | 0.09 | 0.007 | 0.011 | 0.51 | -0.008 | 0.011 | 0.48 | -0.002 | 0.01 | 0.89 | 0.019 | 0.011 | 0.1 |
| rs1411424 | | 9 | 113892963 | A | 0.02 | 0.003 | 1.54E-14 | 0.002 | 0.007 | 0.77 | -0.007 | 0.008 | 0.39 | 0.003 | 0.008 | 0.74 | -0.005 | 0.008 | 0.54 | -0.007 | 0.008 | 0.4 |
| rs2418135 | | 9 | 113901309 | A | 0.02 | 0.003 | 1.53E-14 | 0.001 | 0.007 | 0.86 | -0.007 | 0.008 | 0.38 | 0.004 | 0.008 | 0.63 | -0.004 | 0.008 | 0.6 | -0.009 | 0.008 | 0.29 |
| rs72760655 | | 9 | 116916214 | C | 0.007 | 0.003 | 1.02E-02 | -0.029 | 0.008 | 0 | -0.01 | 0.009 | 0.23 | -0.003 | 0.008 | 0.74 | -0.005 | 0.008 | 0.53 | -0.011 | 0.009 | 0.21 |
| rs1323438 | | 9 | 119115531 | C | 0.019 | 0.003 | 5.58E-11 | -0.001 | 0.008 | 0.9 | 0.024 | 0.009 | 0.01 | 0.011 | 0.009 | 0.21 | 0.022 | 0.009 | 0.02 | 0.014 | 0.009 | 0.11 |
| rs3933326 | | 9 | 123633948 | G | 0.021 | 0.003 | 2.33E-14 | 0.014 | 0.008 | 0.11 | 0.002 | 0.008 | 0.84 | -0.018 | 0.009 | 0.04 | 0.006 | 0.009 | 0.49 | -0.006 | 0.009 | 0.49 |
| rs10985827 | | 9 | 125701608 | G | 0.03 | 0.004 | 6.10E-16 | -0.016 | 0.012 | 0.18 | 0.018 | 0.011 | 0.11 | 0.021 | 0.011 | 0.06 | -0.006 | 0.011 | 0.59 | 0.049 | 0.011 | 0 |
| rs28505901 | | 9 | 139241030 | A | 0.024 | 0.003 | 2.46E-15 | -0.019 | 0.009 | 0.05 | -0.004 | 0.01 | 0.72 | 0.009 | 0.01 | 0.37 | -0.002 | 0.009 | 0.88 | -0.007 | 0.01 | 0.49 |
| rs4350272 | | 10 | 25056118 | A | 0.017 | 0.003 | 3.64E-09 | -0.005 | 0.008 | 0.55 | 0.003 | 0.009 | 0.71 | 0.008 | 0.009 | 0.39 | -0.002 | 0.008 | 0.85 | 0.011 | 0.009 | 0.24 |
| rs5030938 | | 10 | 70975916 | T | 0.024 | 0.003 | 1.23E-17 | 0.007 | 0.008 | 0.39 | -0.007 | 0.009 | 0.45 | -0.002 | 0.009 | 0.82 | 0.008 | 0.009 | 0.38 | -0.03 | 0.009 | 0 |
| rs9645500 | | 10 | 70986723 | G | 0.024 | 0.003 | 1.75E-18 | 0.004 | 0.008 | 0.65 | -0.011 | 0.009 | 0.22 | -0.004 | 0.009 | 0.64 | 0.006 | 0.009 | 0.53 | -0.035 | 0.009 | 0 |
| rs1112718 | | 10 | 94479107 | G | 0.026 | 0.003 | 3.78E-23 | 0.015 | 0.008 | 0.06 | 0.004 | 0.008 | 0.62 | -0.015 | 0.008 | 0.08 | 0 | 0.008 | 0.97 | 0.013 | 0.008 | 0.13 |
| rs10509669 | | 10 | 95969913 | A | -0.001 | 0.003 | 6.85E-01 | -0.017 | 0.009 | 0.06 | 0.021 | 0.01 | 0.03 | 0.012 | 0.01 | 0.23 | 0.02 | 0.01 | 0.04 | 0.011 | 0.01 | 0.27 |
| rs3740360 | | 10 | 96025491 | C | 0.026 | 0.004 | 4.03E-10 | -0.004 | 0.011 | 0.7 | -0.005 | 0.012 | 0.69 | 0.016 | 0.013 | 0.24 | -0.01 | 0.013 | 0.44 | -0.001 | 0.012 | 0.93 |
| rs2274224 | | 10 | 96039597 | C | 0.021 | 0.003 | 9.78E-17 | -0.009 | 0.01 | 0.37 | 0.024 | 0.008 | 0 | 0.015 | 0.008 | 0.08 | 0.022 | 0.008 | 0.01 | 0.008 | 0.008 | 0.32 |
| rs562974282 | | 10 | 104201070 | G | 0.02098 | 0.033593 | 5.32E-01 | - | - | - | - | - | - | - | - | - | - | - | - | - | - | - |
| rs10883846 | | 10 | 104958244 | C | 0.017 | 0.003 | 1.30E-10 | -0.007 | 0.008 | 0.39 | 0.019 | 0.008 | 0.03 | -0.001 | 0.008 | 0.91 | 0.023 | 0.009 | 0.01 | 0.004 | 0.008 | 0.67 |
| rs7903146 | | 10 | 114758349 | T | 0.011 | 0.003 | 5.27E-05 | 0.013 | 0.009 | 0.15 | -0.037 | 0.009 | 0 | 0.005 | 0.009 | 0.58 | -0.031 | 0.009 | 0 | -0.025 | 0.009 | 0.01 |
| rs7076938 | | 10 | 115789375 | T | 0.032 | 0.003 | 2.14E-28 | -0.001 | 0.009 | 0.87 | -0.004 | 0.01 | 0.71 | -0.03 | 0.01 | 0 | 0.002 | 0.009 | 0.87 | -0.005 | 0.01 | 0.63 |
| rs1801253 | | 10 | 115805056 | C | 0.031 | 0.003 | 1.38E-25 | 0.001 | 0.008 | 0.93 | -0.004 | 0.01 | 0.65 | -0.03 | 0.01 | 0 | 0 | 0.009 | 0.98 | -0.004 | 0.01 | 0.7 |
| rs71486610 | | 10 | 124134803 | C | 0.02 | 0.003 | 3.21E-15 | -0.037 | 0.008 | 0 | 0.005 | 0.008 | 0.58 | 0 | 0.008 | 0.99 | -0.002 | 0.008 | 0.85 | 0.016 | 0.008 | 0.05 |
| rs11042596 | | 11 | 2118860 | T | 0.027 | 0.003 | 4.33E-22 | -0.014 | 0.008 | 0.09 | 0.006 | 0.009 | 0.53 | -0.021 | 0.009 | 0.02 | 0.009 | 0.009 | 0.31 | -0.003 | 0.009 | 0.74 |
| rs234864 | | 11 | 2857297 | A | 0.016 | 0.003 | 1.66E-09 | 0.012 | 0.008 | 0.12 | - | - | - | - | - | - | - | - | - | - | - | - |
| rs2168101 | | 11 | 8255408 | C | 0.007 | 0.003 | 1.13E-02 | 0.012 | 0.009 | 0.18 | -0.012 | 0.009 | 0.18 | 0 | 0.008 | 0.96 | -0.012 | 0.009 | 0.17 | -0.003 | 0.009 | 0.71 |
| rs4444073 | | 11 | 10331664 | A | 0.02 | 0.003 | 2.69E-15 | 0.006 | 0.008 | 0.46 | -0.004 | 0.008 | 0.62 | -0.006 | 0.008 | 0.5 | 0.004 | 0.008 | 0.67 | -0.011 | 0.008 | 0.17 |
| rs12574749 | | 11 | 32405355 | C | 0.015 | 0.003 | 1.10E-07 | -0.01 | 0.008 | 0.22 | -0.013 | 0.009 | 0.15 | 0.01 | 0.009 | 0.27 | -0.008 | 0.009 | 0.36 | -0.021 | 0.009 | 0.02 |
| rs5030317 | | 11 | 32410337 | C | 0.017 | 0.003 | 2.71E-09 | 0.012 | 0.009 | 0.18 | 0.017 | 0.009 | 0.06 | -0.008 | 0.009 | 0.4 | 0.015 | 0.009 | 0.11 | 0.017 | 0.009 | 0.08 |
| rs10437653 | | 11 | 46297631 | A | 0.01 | 0.003 | 1.53E-04 | 0.011 | 0.007 | 0.13 | -0.004 | 0.008 | 0.6 | 0.013 | 0.008 | 0.11 | -0.001 | 0.008 | 0.91 | -0.011 | 0.008 | 0.18 |
| rs10734564 | | 11 | 48160429 | A | 0.005 | 0.003 | 1.06E-01 | 0.009 | 0.01 | 0.38 | 0.021 | 0.011 | 0.05 | 0.011 | 0.011 | 0.31 | 0.02 | 0.011 | 0.06 | 0.007 | 0.01 | 0.5 |
| rs667515 | | 11 | 69449076 | G | 0.018 | 0.003 | 9.32E-12 | 0.003 | 0.008 | 0.71 | -0.03 | 0.009 | 0 | -0.01 | 0.009 | 0.24 | -0.03 | 0.009 | 0 | -0.008 | 0.009 | 0.36 |
| rs61885091 | | 11 | 69791952 | A | 0.023 | 0.004 | 4.77E-10 | -0.008 | 0.011 | 0.47 | -0.011 | 0.012 | 0.34 | -0.009 | 0.012 | 0.46 | -0.013 | 0.012 | 0.29 | 0.001 | 0.011 | 0.92 |
| rs10830963 | | 11 | 92708710 | G | 0.019 | 0.003 | 2.79E-11 | 0.005 | 0.009 | 0.57 | -0.006 | 0.009 | 0.51 | -0.002 | 0.009 | 0.81 | -0.007 | 0.009 | 0.44 | -0.001 | 0.009 | 0.92 |
| rs10895278 | | 11 | 102095335 | C | 0.011 | 0.003 | 6.56E-05 | -0.021 | 0.008 | 0.01 | 0.009 | 0.009 | 0.3 | 0.015 | 0.009 | 0.1 | 0.01 | 0.009 | 0.25 | -0.005 | 0.009 | 0.55 |
| rs76895963 | | 12 | 4384844 | G | 0.076 | 0.01 | 2.45E-13 | -0.102 | 0.03 | 0 | 0.114 | 0.033 | 0 | 0.031 | 0.033 | 0.35 | 0.073 | 0.033 | 0.03 | 0.11 | 0.033 | 0 |
| rs11055030 | | 12 | 12878349 | G | 0.02 | 0.003 | 3.85E-12 | 0.005 | 0.008 | 0.53 | -0.006 | 0.009 | 0.51 | 0.001 | 0.009 | 0.92 | -0.007 | 0.009 | 0.47 | -0.006 | 0.009 | 0.55 |
| rs2306547 | | 12 | 26877885 | C | 0.019 | 0.003 | 4.35E-13 | 0.005 | 0.007 | 0.47 | 0.008 | 0.008 | 0.35 | 0.015 | 0.008 | 0.07 | 0.008 | 0.008 | 0.34 | -0.004 | 0.008 | 0.59 |
| rs11051061 | | 12 | 30914668 | A | 0.011 | 0.003 | 1.05E-04 | 0.01 | 0.009 | 0.24 | -0.017 | 0.01 | 0.09 | 0 | 0.009 | 0.97 | -0.024 | 0.01 | 0.02 | 0.011 | 0.01 | 0.27 |
| rs6582623 | | 12 | 46613394 | C | 0.024 | 0.004 | 1.07E-09 | -0.018 | 0.011 | 0.11 | -0.018 | 0.012 | 0.13 | -0.004 | 0.011 | 0.73 | -0.015 | 0.012 | 0.2 | -0.01 | 0.012 | 0.38 |
| rs180438 | | 12 | 47187260 | G | 0.011 | 0.003 | 7.52E-04 | -0.005 | 0.009 | 0.63 | 0.02 | 0.011 | 0.07 | -0.015 | 0.011 | 0.17 | 0.017 | 0.011 | 0.1 | 0.018 | 0.011 | 0.1 |
| rs8756 | | 12 | 66359752 | C | 0.041 | 0.003 | 2.40E-59 | 0.008 | 0.007 | 0.31 | 0.01 | 0.008 | 0.23 | -0.003 | 0.008 | 0.74 | 0.01 | 0.008 | 0.24 | 0.006 | 0.008 | 0.49 |
| rs7968682 | | 12 | 66371880 | G | 0.042 | 0.003 | 4.24E-60 | 0.008 | 0.008 | 0.31 | 0.01 | 0.008 | 0.22 | -0.003 | 0.008 | 0.74 | 0.01 | 0.008 | 0.23 | 0.006 | 0.008 | 0.45 |
| rs1480470 | | 12 | 66412130 | G | 0.024 | 0.003 | 1.37E-19 | -0.001 | 0.008 | 0.91 | 0.019 | 0.008 | 0.02 | 0.016 | 0.009 | 0.06 | 0.024 | 0.009 | 0.01 | -0.002 | 0.008 | 0.81 |
| rs1533688 | | 12 | 102772745 | C | 0.005 | 0.003 | 9.03E-02 | -0.007 | 0.009 | 0.44 | 0.003 | 0.009 | 0.79 | -0.001 | 0.009 | 0.95 | 0.009 | 0.01 | 0.34 | -0.014 | 0.01 | 0.16 |
| rs2647873 | | 12 | 103081192 | A | 0.018 | 0.003 | 2.86E-12 | -0.005 | 0.007 | 0.51 | -0.01 | 0.008 | 0.22 | 0.006 | 0.008 | 0.48 | -0.005 | 0.008 | 0.54 | -0.015 | 0.008 | 0.07 |
| rs17033114 | | 12 | 103123339 | T | 0.016 | 0.006 | 7.75E-03 | -0.007 | 0.017 | 0.67 | -0.051 | 0.016 | 0 | -0.004 | 0.016 | 0.83 | -0.051 | 0.017 | 0 | -0.022 | 0.017 | 0.2 |
| rs3184504 | | 12 | 111884608 | C | 0.023 | 0.003 | 2.57E-19 | 0.021 | 0.008 | 0.01 | -0.024 | 0.008 | 0 | -0.018 | 0.009 | 0.04 | -0.019 | 0.008 | 0.02 | -0.012 | 0.008 | 0.14 |
| rs9549046 | | 13 | 40647206 | A | 0.029 | 0.004 | 7.97E-13 | 0.005 | 0.013 | 0.72 | 0.015 | 0.013 | 0.25 | -0.016 | 0.013 | 0.21 | 0.005 | 0.012 | 0.72 | 0.025 | 0.013 | 0.05 |
| rs34217484 | | 13 | 48854550 | A | 0.019 | 0.003 | 6.76E-11 | 0.007 | 0.009 | 0.4 | 0.01 | 0.009 | 0.24 | -0.006 | 0.009 | 0.54 | 0.014 | 0.009 | 0.12 | -0.002 | 0.008 | 0.85 |
| rs9318511 | | 13 | 78601413 | C | 0.027 | 0.004 | 5.98E-12 | 0.013 | 0.011 | 0.25 | -0.004 | 0.014 | 0.8 | -0.011 | 0.015 | 0.45 | 0.001 | 0.013 | 0.94 | -0.011 | 0.014 | 0.46 |
| rs72681869 | | 14 | 50655357 | C | 0.084 | 0.013 | 5.94E-10 | -0.05 | 0.048 | 0.31 | 0.013 | 0.036 | 0.72 | 0.029 | 0.037 | 0.45 | 0.032 | 0.037 | 0.39 | -0.026 | 0.037 | 0.5 |
| rs6575803 | | 14 | 101257755 | C | 0.032 | 0.004 | 1.29E-12 | 0.01 | 0.013 | 0.46 | 0.006 | 0.011 | 0.62 | 0.014 | 0.012 | 0.22 | 0.001 | 0.011 | 0.96 | 0.007 | 0.011 | 0.57 |
| rs75844534 | | 15 | 38667117 | A | 0.026 | 0.004 | 4.90E-11 | -0.003 | 0.012 | 0.83 | 0.007 | 0.012 | 0.57 | -0.005 | 0.012 | 0.71 | 0.017 | 0.012 | 0.18 | -0.017 | 0.012 | 0.19 |
| rs2928148 | | 15 | 41401550 | A | 0.006 | 0.003 | 1.78E-02 | 0.007 | 0.008 | 0.36 | -0.006 | 0.008 | 0.49 | -0.017 | 0.008 | 0.04 | 0 | 0.007 | 0.97 | -0.006 | 0.008 | 0.47 |
| rs339969 | | 15 | 60883281 | A | 0.017 | 0.003 | 2.18E-10 | -0.007 | 0.008 | 0.4 | 0.012 | 0.009 | 0.16 | -0.001 | 0.008 | 0.89 | 0.014 | 0.009 | 0.1 | 0.002 | 0.008 | 0.77 |
| rs3784789 | | 15 | 75082552 | G | -0.004 | 0.003 | 1.52E-01 | -0.007 | 0.008 | 0.4 | -0.01 | 0.009 | 0.26 | -0.017 | 0.009 | 0.06 | -0.006 | 0.009 | 0.48 | -0.007 | 0.009 | 0.4 |
| rs12909648 | | 15 | 86224570 | G | 0.012 | 0.003 | 1.73E-06 | -0.003 | 0.008 | 0.68 | 0.002 | 0.008 | 0.84 | 0.003 | 0.008 | 0.71 | 0.007 | 0.008 | 0.37 | -0.012 | 0.008 | 0.15 |
| rs12443252 | | 15 | 91064690 | T | 0.006 | 0.003 | 1.68E-02 | 0.023 | 0.008 | 0 | 0.015 | 0.008 | 0.08 | -0.001 | 0.008 | 0.87 | 0.005 | 0.008 | 0.56 | 0.025 | 0.008 | 0 |
| rs7183988 | | 15 | 91428589 | G | 0.018 | 0.003 | 1.67E-12 | 0.008 | 0.008 | 0.37 | 0.008 | 0.008 | 0.34 | -0.012 | 0.008 | 0.16 | 0.006 | 0.008 | 0.45 | 0.01 | 0.008 | 0.21 |
| rs4932373 | | 15 | 91429287 | A | 0.02 | 0.003 | 3.04E-13 | 0.001 | 0.009 | 0.87 | -0.005 | 0.009 | 0.57 | -0.019 | 0.009 | 0.03 | 0 | 0.008 | 0.98 | -0.006 | 0.009 | 0.49 |
| rs55958435 | | 15 | 96852638 | A | 0.025 | 0.003 | 1.56E-16 | -0.015 | 0.009 | 0.09 | 0.001 | 0.009 | 0.92 | 0.013 | 0.01 | 0.16 | -0.002 | 0.009 | 0.84 | 0.004 | 0.009 | 0.65 |
| rs7402983 | | 15 | 99193276 | A | 0.024 | 0.003 | 2.63E-19 | -0.003 | 0.011 | 0.77 | 0.007 | 0.008 | 0.43 | 0.014 | 0.008 | 0.11 | 0.001 | 0.008 | 0.88 | 0.007 | 0.008 | 0.38 |
| rs11630479 | | 15 | 99240481 | G | 0.014 | 0.003 | 8.85E-07 | 0.002 | 0.008 | 0.84 | 0.013 | 0.009 | 0.14 | 0.009 | 0.009 | 0.31 | 0.008 | 0.009 | 0.37 | 0.016 | 0.009 | 0.08 |
| rs2045457 | | 16 | 20046115 | G | 0.016 | 0.003 | 6.32E-09 | -0.004 | 0.008 | 0.59 | 0.014 | 0.008 | 0.11 | 0.009 | 0.009 | 0.29 | 0.016 | 0.009 | 0.06 | -0.005 | 0.008 | 0.6 |
| rs40434 | | 16 | 55699525 | G | 0.017 | 0.003 | 3.00E-10 | -0.002 | 0.008 | 0.76 | -0.006 | 0.008 | 0.47 | 0.001 | 0.008 | 0.94 | 0 | 0.008 | 0.98 | -0.014 | 0.008 | 0.08 |
| rs28544888 | | 16 | 55741204 | C | 0.026 | 0.005 | 1.63E-08 | 0.005 | 0.013 | 0.72 | -0.005 | 0.013 | 0.72 | 0.016 | 0.014 | 0.26 | -0.006 | 0.013 | 0.67 | -0.006 | 0.013 | 0.65 |
| rs11641308 | | 16 | 75312023 | T | 0.007 | 0.003 | 1.26E-02 | 0.016 | 0.008 | 0.05 | -0.015 | 0.009 | 0.07 | -0.013 | 0.009 | 0.13 | -0.016 | 0.009 | 0.07 | -0.002 | 0.008 | 0.78 |
| rs222857 | | 17 | 7164563 | T | 0.026 | 0.003 | 1.06E-24 | -0.007 | 0.008 | 0.41 | -0.01 | 0.008 | 0.23 | 0.001 | 0.008 | 0.88 | -0.003 | 0.008 | 0.72 | -0.016 | 0.008 | 0.05 |
| rs2428362 | | 17 | 7180274 | T | 0.025 | 0.003 | 1.80E-22 | - | - | - | - | - | - | - | - | - | - | - | - | - | - | - |
| rs4511593 | | 17 | 7455536 | T | 0.017 | 0.003 | 1.09E-10 | 0.001 | 0.008 | 0.93 | 0.005 | 0.008 | 0.59 | 0.019 | 0.009 | 0.03 | 0.002 | 0.008 | 0.84 | 0.004 | 0.008 | 0.66 |
| rs78378222 | | 17 | 7571752 | G | 0.079 | 0.012 | 1.83E-11 | -0.048 | 0.035 | 0.18 | 0.02 | 0.029 | 0.5 | -0.006 | 0.028 | 0.84 | 0.007 | 0.028 | 0.82 | 0.037 | 0.03 | 0.23 |
| rs9909342 | | 17 | 25652275 | A | 0.018 | 0.003 | 2.17E-11 | 0.003 | 0.008 | 0.71 | 0.015 | 0.008 | 0.07 | -0.008 | 0.008 | 0.33 | 0.01 | 0.008 | 0.25 | 0.018 | 0.009 | 0.03 |
| rs7223535 | | 17 | 29211667 | G | 0.021 | 0.003 | 2.12E-13 | -0.015 | 0.009 | 0.08 | -0.014 | 0.009 | 0.13 | 0.001 | 0.009 | 0.93 | 0.005 | 0.009 | 0.63 | -0.04 | 0.009 | 0 |
| rs11867479 | | 17 | 68090207 | T | 0.017 | 0.003 | 1.08E-10 | -0.01 | 0.008 | 0.23 | 0.003 | 0.008 | 0.7 | -0.007 | 0.009 | 0.43 | -0.006 | 0.008 | 0.52 | 0.024 | 0.009 | 0.01 |
| rs10221267 | | 17 | 68464662 | T | 0.017 | 0.003 | 6.52E-11 | -0.022 | 0.008 | 0 | -0.002 | 0.008 | 0.79 | -0.008 | 0.008 | 0.35 | -0.002 | 0.008 | 0.8 | 0.007 | 0.008 | 0.39 |
| rs73354194 | | 17 | 79905947 | C | 0.061 | 0.009 | 1.01E-11 | -0.019 | 0.026 | 0.48 | -0.027 | 0.027 | 0.33 | 0.029 | 0.028 | 0.3 | -0.047 | 0.028 | 0.09 | 0.008 | 0.026 | 0.77 |
| rs9912553 | | 17 | 79959703 | G | 0.014 | 0.003 | 1.74E-06 | 0.019 | 0.009 | 0.04 | 0.022 | 0.009 | 0.02 | 0.017 | 0.009 | 0.06 | 0.006 | 0.009 | 0.54 | 0.038 | 0.009 | 0 |
| rs11082304 | | 18 | 20720973 | T | 0.016 | 0.003 | 4.22E-10 | -0.004 | 0.008 | 0.59 | 0.01 | 0.008 | 0.24 | -0.003 | 0.008 | 0.68 | 0.011 | 0.008 | 0.16 | 0.005 | 0.008 | 0.53 |
| rs2779165 | | 19 | 4915447 | G | 0.022 | 0.003 | 7.59E-11 | 0.003 | 0.01 | 0.78 | 0.004 | 0.01 | 0.71 | 0 | 0.01 | 0.99 | 0.007 | 0.01 | 0.51 | -0.004 | 0.01 | 0.7 |
| rs8106042 | | 19 | 7161849 | G | 0.02 | 0.003 | 2.17E-12 | 0.009 | 0.009 | 0.32 | 0.008 | 0.009 | 0.4 | 0.01 | 0.009 | 0.29 | 0.004 | 0.009 | 0.68 | 0.01 | 0.009 | 0.27 |
| rs2967676 | | 19 | 8789666 | A | 0.021 | 0.004 | 1.14E-08 | 0.027 | 0.01 | 0.01 | 0.002 | 0.011 | 0.83 | 0.005 | 0.011 | 0.64 | 0.003 | 0.011 | 0.77 | 0.005 | 0.011 | 0.66 |
| rs41355649 | | 19 | 33790556 | G | 0.034 | 0.005 | 1.17E-10 | -0.011 | 0.016 | 0.49 | 0.012 | 0.016 | 0.49 | 0.029 | 0.017 | 0.09 | 0.018 | 0.017 | 0.29 | -0.017 | 0.017 | 0.31 |
| rs1129156 | | 19 | 40719076 | T | 0.017 | 0.003 | 2.53E-09 | -0.015 | 0.008 | 0.08 | -0.013 | 0.009 | 0.15 | -0.008 | 0.009 | 0.41 | -0.007 | 0.009 | 0.46 | -0.018 | 0.009 | 0.06 |
| rs147957154 | | 19 | 43431040 | T | 0.023 | 0.004 | 2.75E-09 | 0.001 | 0.011 | 0.9 | - | - | - | - | - | - | - | - | - | - | - | - |
| rs516246 | | 19 | 49206172 | C | 0.018 | 0.003 | 9.34E-12 | 0.015 | 0.008 | 0.05 | 0.007 | 0.008 | 0.39 | -0.004 | 0.008 | 0.6 | 0.01 | 0.008 | 0.26 | 0.001 | 0.008 | 0.87 |
| rs255773 | | 19 | 54723546 | C | 0.018 | 0.003 | 1.32E-11 | 0.004 | 0.008 | 0.62 | -0.002 | 0.008 | 0.83 | 0.001 | 0.008 | 0.88 | -0.007 | 0.008 | 0.41 | 0.009 | 0.008 | 0.29 |
| rs147110934 | | 19 | 55993436 | G | 0.052 | 0.009 | 1.62E-09 | -0.013 | 0.032 | 0.68 | 0.051 | 0.044 | 0.25 | 0.078 | 0.045 | 0.09 | -0.018 | 0.043 | 0.69 | 0.133 | 0.045 | 0 |
| rs12461110 | | 19 | 56320663 | A | 0.006 | 0.003 | 2.76E-02 | 0.005 | 0.008 | 0.54 | -0.009 | 0.008 | 0.29 | -0.008 | 0.008 | 0.38 | -0.006 | 0.008 | 0.45 | -0.006 | 0.008 | 0.45 |
| rs304001 | | 19 | 56423668 | G | 0.009 | 0.003 | 8.52E-04 | -0.011 | 0.007 | 0.13 | -0.006 | 0.008 | 0.46 | -0.009 | 0.008 | 0.27 | -0.006 | 0.008 | 0.49 | 0.002 | 0.008 | 0.85 |
| rs6040076 | | 20 | 10658882 | C | 0.019 | 0.003 | 4.35E-13 | 0.013 | 0.008 | 0.1 | 0.001 | 0.008 | 0.89 | -0.004 | 0.008 | 0.6 | -0.001 | 0.008 | 0.9 | 0.004 | 0.008 | 0.6 |
| rs6033062 | | 20 | 11207419 | A | 0.016 | 0.003 | 5.17E-10 | 0.005 | 0.007 | 0.49 | 0.001 | 0.008 | 0.9 | -0.014 | 0.008 | 0.1 | 0.011 | 0.008 | 0.2 | -0.016 | 0.008 | 0.06 |
| rs1203876 | | 20 | 22540915 | C | 0.038 | 0.006 | 9.44E-10 | -0.007 | 0.017 | 0.7 | 0.02 | 0.022 | 0.37 | -0.005 | 0.021 | 0.82 | 0.006 | 0.021 | 0.78 | 0.05 | 0.022 | 0.02 |
| rs11698914 | | 20 | 31327144 | C | 0.032 | 0.003 | 1.21E-24 | -0.002 | 0.009 | 0.81 | 0.018 | 0.01 | 0.06 | 0 | 0.009 | 0.97 | 0.017 | 0.01 | 0.09 | 0.011 | 0.01 | 0.28 |
| rs181451002 | | 20 | 32466219 | G | 0.02 | 0.009 | 2.63E-02 | -0.022 | 0.032 | 0.5 | - | - | - | - | - | - | - | - | - | - | - | - |
| rs2889874 | | 20 | 33715777 | G | 0.016 | 0.003 | 9.44E-10 | 0.01 | 0.008 | 0.21 | 0.009 | 0.008 | 0.27 | 0.011 | 0.008 | 0.18 | -0.003 | 0.008 | 0.72 | 0.023 | 0.008 | 0.01 |
| rs1012167 | | 20 | 39159119 | C | 0.024 | 0.003 | 1.20E-19 | -0.009 | 0.008 | 0.24 | 0.005 | 0.008 | 0.56 | -0.011 | 0.009 | 0.19 | 0.008 | 0.008 | 0.33 | 0 | 0.008 | 0.97 |
| rs753381 | | 20 | 39797465 | T | 0.015 | 0.003 | 3.43E-09 | -0.003 | 0.007 | 0.69 | 0.014 | 0.008 | 0.08 | 0.011 | 0.008 | 0.21 | 0.011 | 0.008 | 0.17 | 0.008 | 0.008 | 0.31 |
| rs6026449 | | 20 | 57272617 | C | 0.017 | 0.003 | 2.49E-10 | 0 | 0.008 | 0.99 | -0.004 | 0.008 | 0.66 | -0.013 | 0.008 | 0.11 | 0 | 0.008 | 0.98 | -0.004 | 0.008 | 0.6 |
| rs73143584 | | 20 | 62445702 | A | 0.029 | 0.004 | 1.80E-11 | 0.001 | 0.012 | 0.91 | -0.002 | 0.011 | 0.88 | -0.004 | 0.011 | 0.73 | 0.003 | 0.011 | 0.8 | -0.011 | 0.012 | 0.35 |
| rs2229742 | | 21 | 16339172 | G | 0.027 | 0.004 | 7.35E-11 | 0.004 | 0.012 | 0.73 | -0.017 | 0.013 | 0.21 | 0.002 | 0.013 | 0.9 | -0.027 | 0.013 | 0.04 | 0.01 | 0.013 | 0.47 |
| rs220193 | | 21 | 43581308 | A | 0.021 | 0.003 | 4.07E-11 | 0.009 | 0.009 | 0.35 | 0.012 | 0.01 | 0.24 | 0.006 | 0.01 | 0.56 | 0.022 | 0.01 | 0.03 | -0.021 | 0.01 | 0.04 |
| rs134594 | | 22 | 29468456 | C | 0.017 | 0.003 | 5.76E-10 | 0.002 | 0.01 | 0.82 | 0 | 0.008 | 0.96 | 0.012 | 0.009 | 0.16 | 0.001 | 0.008 | 0.91 | -0.006 | 0.008 | 0.52 |
| rs41311445 | | 22 | 42070374 | A | 0.033 | 0.004 | 3.33E-13 | -0.009 | 0.015 | 0.57 | 0.013 | 0.013 | 0.31 | 0.011 | 0.013 | 0.4 | 0.007 | 0.013 | 0.61 | 0.012 | 0.013 | 0.36 |
| rs7285579 | | 22 | 46441980 | C | 0.017 | 0.003 | 2.66E-09 | -0.013 | 0.009 | 0.18 | 0.028 | 0.009 | 0 | 0.016 | 0.009 | 0.09 | 0.018 | 0.009 | 0.05 | 0.023 | 0.009 | 0.01 |

**Table S8.** P Values for Associations of 205 birth weight variants with potential risk factors

| **SNP** | **CHR** | | **BP** | P_Alcohol_dependence (41) | P_AN (42) | P_BMI (43) | P_CigarettesPerDay (44) | P_College_compile (45) | P_DrinksPerWeek (44) | P_EduYears (46) | P_UC (47) | P_IBD (47) | P_CD (47) | P_VD (48) | P_RA (49) |
| --- | --- | --- | --- | --- | --- | --- | --- | --- | --- | --- | --- | --- | --- | --- | --- |
| rs17367504 | | 1 | 11862778 | 8.83E-01 | 1.48E-01 | 2.18E-02 | 3.39E-01 | 5.44E-01 | 4.57E-02 | 8.13E-01 | 2.43E-01 | 2.08E-01 | 1.99E-01 | 2.94E-04 | 3.21E-02 |
| rs12401656 | | 1 | 43456767 | 4.14E-01 | 1.71E-01 | - | 8.10E-01 | - | 9.57E-01 | - | 5.16E-01 | 1.87E-01 | 8.13E-02 | 3.02E-01 | 3.81E-01 |
| rs80278614 | | 1 | 119412317 | 7.69E-01 | 6.06E-01 | - | 7.27E-03 | - | 2.03E-01 | - | 4.35E-01 | 5.36E-01 | 8.90E-01 | 1.18E-01 | - |
| rs905938 | | 1 | 154991389 | 5.89E-01 | 5.30E-01 | 9.78E-05 | 3.04E-01 | 6.85E-01 | 1.93E-01 | 5.45E-01 | 1.35E-01 | 4.27E-02 | 1.64E-01 | 1.37E-04 | 3.47E-02 |
| rs670523 | | 1 | 155878732 | 4.52E-01 | 7.38E-02 | 1.16E-02 | 4.57E-01 | 5.49E-01 | 1.34E-01 | 7.71E-01 | 1.71E-01 | 2.39E-03 | 1.21E-05 | - | 6.01E-01 |
| rs72480273 | | 1 | 161644871 | 6.50E-01 | 2.73E-01 | - | 4.74E-02 | - | 8.34E-01 | - | 4.19E-01 | 5.46E-01 | 8.10E-01 | 4.80E-02 | 1.52E-02 |
| rs10913200 | | 1 | 176521655 | 8.24E-01 | 3.15E-01 | - | 3.54E-01 | - | 4.31E-01 | - | 5.76E-01 | 3.77E-01 | 6.62E-01 | 2.53E-01 | 9.97E-01 |
| rs61830764 | | 1 | 212289976 | 2.51E-01 | 2.57E-01 | - | 9.06E-01 | - | 6.05E-01 | - | 7.49E-01 | 6.02E-01 | 1.29E-01 | 1.99E-01 | 6.29E-01 |
| rs3806315 | | 1 | 214724668 | 7.51E-02 | 8.44E-01 | - | 6.46E-01 | - | - | - | 7.11E-02 | 3.90E-02 | 1.11E-01 | 2.37E-01 | 4.45E-01 |
| rs708122 | | 1 | 228216997 | 3.08E-01 | 8.10E-01 | 9.03E-01 | 3.55E-01 | 4.80E-01 | 9.54E-01 | 1.69E-01 | 6.69E-01 | 5.94E-01 | 3.15E-01 | 6.66E-01 | 9.28E-01 |
| rs10495563 | | 2 | 9662210 | 4.27E-01 | 5.02E-01 | 8.73E-01 | 8.11E-01 | 4.36E-02 | 2.50E-01 | 1.43E-01 | 1.58E-02 | 1.01E-02 | 6.20E-01 | 7.16E-01 | 9.06E-01 |
| rs11893688 | | 2 | 9695282 | 4.73E-01 | 4.38E-01 | 6.17E-01 | 7.60E-01 | 2.57E-02 | 6.71E-01 | 9.58E-02 | 1.20E-02 | 7.62E-03 | 5.61E-01 | 7.00E-01 | 9.06E-01 |
| rs2551347 | | 2 | 23912401 | 6.52E-02 | 9.24E-01 | - | 9.20E-01 | - | 6.54E-05 | - | 6.91E-01 | 6.84E-01 | 7.89E-01 | 8.84E-03 | 6.27E-01 |
| rs1179494 | | 2 | 36809496 | 3.62E-01 | 4.12E-01 | 3.55E-01 | 8.70E-01 | 4.37E-01 | 7.99E-01 | 1.72E-01 | 5.13E-01 | 3.56E-01 | 3.94E-01 | 9.71E-01 | 9.79E-01 |
| rs754868 | | 2 | 43185532 | 6.91E-01 | 1.12E-01 | 2.65E-01 | 1.52E-01 | 1.37E-01 | 8.09E-01 | 6.95E-01 | 1.55E-01 | 3.31E-01 | 7.04E-01 | 3.46E-01 | 4.54E-01 |
| rs4952673 | | 2 | 43423870 | 8.25E-01 | 7.80E-01 | 1.47E-01 | 1.84E-01 | 8.20E-01 | 1.10E-01 | 9.53E-01 | 5.18E-01 | 9.30E-01 | 6.88E-01 | 8.67E-01 | 4.25E-02 |
| rs17034876 | | 2 | 46484310 | 2.69E-01 | 6.59E-01 | 3.06E-01 | 9.17E-01 | 3.61E-01 | 1.34E-01 | 8.27E-01 | 8.51E-01 | 6.54E-01 | 9.74E-01 | 2.55E-01 | 8.73E-01 |
| rs4953353 | | 2 | 46567276 | 1.33E-02 | 9.96E-01 | 5.49E-01 | 5.80E-01 | 6.03E-01 | 4.19E-01 | 1.57E-01 | 2.06E-01 | 2.70E-01 | 7.29E-01 | 2.82E-01 | 8.12E-01 |
| rs186606513 | | 2 | 97482001 | - | 7.67E-01 | - | 2.08E-01 | - | 7.47E-01 | - | 8.29E-01 | 5.66E-01 | 2.74E-01 | 6.68E-01 | - |
| rs56188432 | | 2 | 158406865 | - | - | - | - | - | - | - | - | - | - | 5.75E-01 | - |
| rs560887 | | 2 | 169763148 | 5.95E-01 | 3.93E-01 | 2.84E-01 | 5.50E-01 | 9.10E-01 | 1.35E-01 | 9.20E-01 | 5.96E-01 | 5.01E-01 | 7.35E-02 | 4.79E-01 | 9.99E-01 |
| rs2280235 | | 2 | 191843830 | 4.59E-01 | 1.29E-02 | 1.22E-01 | 5.53E-02 | 6.80E-01 | 6.31E-01 | 3.01E-01 | 7.87E-01 | 3.11E-01 | 2.08E-01 | 8.62E-02 | 8.07E-04 |
| rs10181515 | | 2 | 227019461 | 3.30E-01 | 2.30E-02 | 9.71E-05 | 6.46E-01 | 5.76E-01 | 6.17E-02 | 8.70E-01 | 4.91E-01 | 6.86E-01 | 9.62E-01 | 9.57E-02 | 2.43E-01 |
| rs9855896 | | 3 | 14287150 | 1.69E-01 | 1.30E-01 | 9.83E-01 | 6.98E-01 | 5.35E-01 | 7.28E-01 | 6.55E-01 | 7.02E-01 | 2.37E-01 | 5.30E-02 | 8.44E-01 | 3.52E-01 |
| rs2168443 | | 3 | 46947087 | 8.34E-01 | 4.57E-01 | - | 7.90E-01 | - | 7.75E-01 | - | 1.37E-01 | 5.25E-01 | 2.63E-01 | 1.48E-02 | 9.32E-01 |
| rs11708067 | | 3 | 123065778 | 6.26E-01 | 3.98E-02 | 6.23E-05 | 1.09E-01 | 7.32E-01 | 4.99E-01 | 8.43E-01 | 4.34E-01 | 2.48E-01 | 5.19E-01 | 9.10E-01 | 3.05E-01 |
| rs9851257 | | 3 | 123125711 | 5.17E-01 | 6.07E-01 | - | 3.76E-02 | - | 1.11E-01 | - | 6.07E-01 | 5.55E-01 | 8.51E-01 | 7.29E-01 | 3.79E-01 |
| rs6440006 | | 3 | 141142691 | 3.99E-01 | 6.53E-01 | 7.35E-02 | 1.54E-01 | 4.87E-01 | 4.68E-01 | 2.01E-01 | 4.31E-01 | 7.79E-02 | 1.57E-04 | 1.20E-04 | 5.93E-02 |
| rs2306700 | | 3 | 142123841 | 2.38E-01 | 4.20E-02 | 8.65E-02 | 1.46E-01 | 7.10E-01 | 2.35E-04 | 9.51E-01 | 4.11E-01 | 8.61E-01 | 1.75E-01 | 7.85E-01 | 4.12E-01 |
| rs10935733 | | 3 | 148622968 | 9.62E-01 | 2.86E-01 | - | 4.70E-01 | - | 1.88E-01 | - | 8.09E-01 | 8.89E-01 | 3.95E-01 | 8.56E-02 | 1.40E-01 |
| rs4679760 | | 3 | 155855418 | 2.72E-01 | 2.19E-01 | - | - | - | - | - | 5.81E-01 | 2.67E-01 | 3.01E-01 | 5.18E-01 | 9.85E-01 |
| rs1482852 | | 3 | 156798294 | 9.51E-01 | 1.35E-01 | 3.01E-01 | 5.22E-01 | 2.87E-01 | 5.72E-01 | 2.90E-01 | 4.47E-01 | 3.87E-01 | 4.07E-01 | 4.98E-02 | 2.16E-01 |
| rs11711420 | | 3 | 183349010 | 1.13E-01 | 2.82E-03 | - | 8.55E-01 | - | 1.70E-01 | - | 1.43E-01 | 5.18E-02 | 1.01E-01 | 8.39E-01 | 2.64E-01 |
| rs4144829 | | 4 | 17903654 | 1.85E-01 | 4.91E-01 | - | 7.24E-02 | - | 5.91E-01 | - | 3.83E-01 | 2.31E-01 | 5.29E-01 | 1.02E-01 | 9.28E-01 |
| rs2174633 | | 4 | 17917781 | 1.81E-01 | 4.80E-01 | 9.43E-01 | 8.42E-02 | 6.26E-03 | 6.04E-01 | 1.27E-03 | 3.85E-01 | 2.07E-01 | 4.66E-01 | 1.43E-01 | 9.50E-01 |
| rs2189234 | | 4 | 106075498 | 1.92E-01 | 9.01E-02 | 6.89E-01 | 2.28E-02 | 3.76E-03 | 3.79E-03 | 2.74E-03 | 9.77E-05 | 2.25E-04 | 3.40E-02 | 2.20E-01 | 8.30E-01 |
| rs6533183 | | 4 | 106133184 | 3.79E-01 | 8.97E-01 | - | 3.04E-01 | - | 7.96E-04 | - | 5.27E-03 | 3.73E-02 | 8.14E-01 | 7.87E-01 | 2.74E-01 |
| rs116807401 | | 4 | 135121721 | 7.99E-01 | 6.64E-01 | - | 2.45E-01 | - | 3.47E-01 | - | 5.06E-01 | 2.82E-01 | 2.44E-01 | 4.44E-01 | - |
| rs6845999 | | 4 | 145565826 | 7.10E-01 | 6.52E-01 | 5.55E-01 | 7.86E-01 | 8.82E-01 | 2.00E-01 | 7.73E-01 | 3.23E-01 | 1.02E-01 | 1.42E-02 | 9.71E-02 | 6.24E-01 |
| rs2131354 | | 4 | 145599908 | 2.18E-01 | 5.17E-01 | - | 9.31E-01 | - | 1.77E-01 | - | 5.75E-01 | 2.67E-01 | 5.10E-02 | 1.09E-01 | 7.84E-01 |
| rs4579095 | | 4 | 174726635 | 8.63E-01 | 5.55E-01 | 6.35E-01 | 9.45E-01 | 9.88E-02 | 7.99E-01 | 2.07E-01 | 5.73E-01 | 9.37E-01 | 8.55E-01 | 6.37E-01 | 2.95E-02 |
| rs1818782 | | 5 | 39424628 | 3.89E-01 | 3.50E-01 | - | 5.88E-01 | - | 4.55E-02 | - | 8.39E-01 | 3.56E-01 | 1.77E-01 | 1.32E-04 | 7.35E-01 |
| rs351930 | | 5 | 52003397 | 8.77E-01 | 2.53E-02 | 9.49E-01 | 7.35E-01 | 5.59E-02 | 5.34E-01 | 6.29E-01 | 4.87E-01 | 9.66E-01 | 8.47E-01 | 6.18E-01 | 5.34E-02 |
| rs854037 | | 5 | 57091783 | 6.24E-01 | 5.84E-01 | 4.53E-01 | 5.98E-01 | 1.34E-02 | 7.00E-01 | 1.14E-01 | 7.21E-01 | 9.25E-01 | 2.86E-01 | 2.26E-01 | 1.66E-03 |
| rs28365970 | | 5 | 67585723 | 4.68E-01 | 8.00E-01 | - | 6.70E-01 | - | 9.90E-01 | - | 2.41E-03 | 1.36E-01 | 2.62E-01 | 2.49E-02 | 4.36E-01 |
| rs6871635 | | 5 | 133830395 | 6.33E-02 | 4.50E-01 | 8.91E-02 | 2.64E-01 | 3.42E-02 | 9.27E-01 | 4.14E-01 | 2.09E-01 | 1.40E-01 | 7.73E-01 | 1.74E-02 | 2.21E-01 |
| rs1981627 | | 5 | 133838180 | 1.80E-01 | 6.51E-01 | 4.84E-02 | 1.19E-01 | 5.49E-02 | 7.05E-01 | 5.82E-01 | 3.22E-01 | 3.22E-01 | 9.55E-01 | 2.89E-02 | 2.81E-01 |
| rs2946179 | | 5 | 157886627 | 9.46E-01 | 2.39E-01 | - | 6.98E-01 | - | 4.68E-01 | - | 2.41E-01 | 7.64E-03 | 5.02E-03 | 2.60E-01 | 5.38E-01 |
| rs34471628 | | 5 | 172196752 | 9.82E-01 | 6.70E-01 | - | 1.63E-01 | - | 3.44E-02 | - | 4.21E-01 | 8.53E-04 | 1.38E-06 | 3.10E-01 | 5.37E-01 |
| rs9379084 | | 6 | 7231843 | 6.60E-01 | 8.99E-01 | 3.03E-03 | 2.62E-02 | 5.66E-01 | 4.32E-01 | 7.40E-01 | 7.57E-01 | 6.39E-01 | 6.96E-01 | 8.60E-05 | 4.26E-01 |
| rs35261542 | | 6 | 20675792 | 5.94E-01 | 3.25E-01 | - | 8.97E-01 | - | 9.69E-01 | - | 6.20E-01 | 4.16E-01 | 1.19E-01 | 1.60E-01 | 3.65E-01 |
| rs9379832 | | 6 | 26186200 | 3.52E-01 | 8.76E-01 | - | 1.95E-06 | - | 4.21E-01 | - | 5.43E-03 | 6.70E-02 | 3.63E-01 | 4.39E-01 | 5.39E-02 |
| rs9366778 | | 6 | 31269173 | 5.48E-01 | 7.94E-02 | 1.03E-01 | 6.48E-03 | 8.72E-01 | 1.53E-01 | 7.39E-01 | - | - | - | 1.15E-01 | 4.56E-04 |
| rs6911024 | | 6 | 31368451 | 2.17E-01 | 9.61E-01 | 8.65E-01 | 9.53E-01 | 1.24E-01 | 4.19E-01 | 1.71E-02 | - | - | - | 9.30E-05 | 1.03E-08 |
| rs9267812 | | 6 | 32128394 | 2.67E-02 | 2.15E-01 | - | 3.23E-01 | - | 5.32E-01 | - | - | - | - | 9.91E-02 | 1.76E-73 |
| rs1547669 | | 6 | 33775641 | 2.26E-01 | 7.99E-03 | 5.81E-01 | 5.31E-01 | 3.56E-03 | 3.99E-04 | 2.71E-04 | 6.33E-01 | 6.81E-01 | 1.58E-01 | 8.13E-03 | 1.63E-27 |
| rs75104038 | | 6 | 34190104 | 1.44E-01 | 5.72E-03 | - | 7.35E-01 | - | 6.79E-01 | - | 6.31E-01 | 3.90E-01 | 2.13E-01 | 1.23E-03 | - |
| rs75034466 | | 6 | 34199815 | 4.84E-01 | 1.01E-01 | - | 4.02E-01 | - | 8.98E-01 | - | 1.71E-01 | 2.13E-01 | 4.25E-01 | 1.94E-03 | - |
| rs6911621 | | 6 | 35529025 | 1.69E-01 | 4.16E-01 | 8.01E-02 | 5.66E-01 | 6.45E-01 | 4.35E-02 | 3.69E-01 | 6.22E-02 | 1.32E-01 | 8.39E-01 | 8.49E-02 | 7.44E-01 |
| rs9348981 | | 6 | 35687249 | 3.39E-01 | 7.69E-01 | - | 2.45E-01 | - | 5.89E-01 | - | 5.40E-02 | 2.21E-02 | 3.07E-01 | 9.91E-01 | 5.85E-02 |
| rs7744700 | | 6 | 53349401 | 9.34E-01 | 9.61E-01 | 1.87E-01 | 6.65E-01 | 1.63E-01 | 7.24E-02 | 5.35E-02 | 5.13E-01 | 6.35E-01 | 9.59E-01 | 6.47E-02 | 6.61E-01 |
| rs76094073 | | 6 | 109288036 | 3.50E-01 | 4.91E-01 | - | 7.63E-02 | - | 3.96E-01 | - | 1.58E-02 | 3.94E-02 | 8.15E-01 | 7.87E-02 | - |
| rs6568554 | | 6 | 109290319 | 9.19E-01 | 7.84E-01 | - | - | - | - | - | 5.90E-02 | 1.77E-01 | 9.09E-01 | 4.45E-02 | 7.82E-02 |
| rs6925689 | | 6 | 126865884 | 7.02E-01 | 1.44E-01 | - | 3.45E-01 | - | 5.94E-01 | - | 9.72E-01 | 7.68E-02 | 6.61E-02 | 2.28E-01 | 4.98E-01 |
| rs6569647 | | 6 | 130337266 | 2.40E-02 | 7.34E-01 | 5.46E-01 | 1.01E-01 | 5.44E-02 | 7.80E-01 | 3.76E-01 | 9.09E-01 | 7.91E-01 | 4.23E-01 | 4.86E-01 | 9.52E-01 |
| rs1415701 | | 6 | 130345835 | 1.60E-01 | 7.52E-01 | 4.29E-02 | 1.96E-01 | 6.78E-01 | 3.73E-01 | 8.54E-01 | 4.02E-01 | 2.28E-01 | 2.87E-01 | 2.04E-01 | 1.15E-01 |
| rs6930558 | | 6 | 141878920 | 2.29E-02 | 1.73E-01 | 1.56E-01 | 3.54E-01 | 1.66E-01 | 9.28E-01 | 9.41E-01 | 1.48E-01 | 2.98E-01 | 7.35E-01 | 4.78E-01 | 3.69E-01 |
| rs962554 | | 6 | 142734204 | 6.10E-01 | 3.27E-01 | 1.11E-01 | 7.15E-01 | 5.79E-01 | 6.27E-01 | 8.36E-02 | 4.75E-01 | 3.16E-01 | 2.90E-01 | 5.29E-01 | 5.95E-01 |
| rs10872678 | | 6 | 152039964 | 1.42E-01 | 2.69E-01 | 8.70E-01 | 9.94E-01 | 4.58E-01 | 6.01E-01 | 9.16E-01 | 6.80E-01 | 9.96E-01 | 9.98E-01 | 3.43E-01 | 7.47E-01 |
| rs7772579 | | 6 | 152042502 | 1.51E-01 | 2.14E-01 | 6.51E-01 | 9.58E-01 | 6.54E-01 | 6.14E-01 | 8.58E-01 | 5.96E-01 | 9.36E-01 | 9.08E-01 | 4.57E-01 | 7.33E-01 |
| rs2934844 | | 6 | 166142456 | 1.91E-01 | 8.00E-01 | - | 4.68E-01 | - | 1.72E-01 | - | 1.45E-01 | 1.57E-01 | 5.94E-01 | 2.48E-01 | 8.70E-01 |
| rs1724889 | | 7 | 2741021 | 9.00E-01 | 5.61E-01 | - | 1.37E-01 | - | 5.49E-01 | - | 1.83E-02 | 7.50E-02 | 8.69E-01 | 9.20E-01 | 6.64E-02 |
| rs4719648 | | 7 | 2756832 | 1.39E-01 | 4.75E-01 | - | 2.46E-01 | - | 4.42E-01 | - | 1.49E-02 | 6.46E-02 | 8.98E-01 | 2.42E-01 | 4.72E-01 |
| rs59084784 | | 7 | 22739562 | 8.15E-01 | 4.06E-01 | - | 7.78E-01 | - | 2.76E-01 | - | 1.07E-03 | 2.51E-04 | 1.77E-02 | 3.87E-01 | 8.47E-01 |
| rs7808457 | | 7 | 22798265 | 5.35E-01 | 7.56E-01 | 1.41E-01 | 3.57E-01 | 7.48E-02 | 3.08E-02 | 3.57E-01 | 8.96E-02 | 1.37E-01 | 7.63E-01 | 1.55E-02 | 8.11E-01 |
| rs34776209 | | 7 | 23513093 | 7.41E-01 | 9.26E-01 | - | 6.37E-04 | - | 7.10E-03 | - | 9.22E-01 | 6.20E-01 | 2.12E-01 | 6.95E-01 | 5.20E-01 |
| rs2908279 | | 7 | 44174857 | 1.68E-01 | 2.03E-01 | - | 1.54E-01 | - | 3.33E-01 | - | 1.93E-01 | 3.02E-02 | 3.12E-02 | 4.41E-02 | 7.67E-01 |
| rs2971669 | | 7 | 44231778 | 3.14E-02 | 8.96E-01 | 4.23E-01 | 4.25E-01 | 1.80E-01 | 4.68E-02 | 4.11E-01 | 5.51E-02 | 8.68E-02 | 5.78E-01 | 2.72E-02 | 4.76E-01 |
| rs138715366 | | 7 | 44246271 | - | - | - | 6.16E-01 | - | 8.13E-01 | - | 5.98E-01 | 6.36E-01 | 7.12E-01 | 8.81E-01 | - |
| rs10265133 | | 7 | 45895604 | 8.05E-02 | - | - | 7.73E-01 | - | 7.86E-01 | - | 1.88E-01 | 7.87E-01 | 2.02E-01 | 3.14E-01 | 7.88E-01 |
| rs11983722 | | 7 | 46298647 | 7.11E-01 | 4.59E-02 | 9.43E-01 | 1.11E-01 | 6.65E-01 | 3.91E-01 | 4.16E-01 | 7.12E-01 | 7.07E-01 | 9.33E-01 | 7.51E-01 | 4.53E-01 |
| rs10265057 | | 7 | 47275737 | 2.56E-01 | - | 7.64E-01 | 1.21E-01 | 6.97E-01 | 6.58E-01 | 3.10E-01 | 5.79E-01 | 5.29E-01 | 2.94E-01 | 4.89E-02 | 8.51E-01 |
| rs2237467 | | 7 | 50733316 | 5.79E-01 | 4.72E-02 | 5.57E-02 | 9.73E-01 | 2.04E-01 | 9.85E-01 | 2.11E-01 | 7.12E-01 | 5.27E-01 | 6.97E-01 | 7.38E-01 | 3.83E-01 |
| rs112139215 | | 7 | 73034559 | 5.56E-01 | 1.96E-01 | - | 7.90E-01 | - | 2.72E-01 | - | 9.94E-01 | 3.29E-01 | 4.93E-02 | 3.56E-01 | - |
| rs2282978 | | 7 | 92264410 | 8.62E-01 | 2.21E-01 | 9.87E-01 | 1.20E-02 | 3.92E-01 | 2.34E-01 | 1.07E-01 | 1.01E-01 | 2.58E-02 | 2.55E-02 | 5.67E-01 | 3.21E-05 |
| rs45446698 | | 7 | 99332948 | 4.70E-01 | 9.71E-01 | - | 1.11E-03 | - | 9.21E-01 | - | 1.09E-02 | 4.43E-03 | 2.34E-02 | 5.26E-01 | 1.34E-01 |
| rs13231367 | | 7 | 127509070 | 7.59E-01 | 8.03E-01 | 7.75E-01 | 8.36E-02 | 1.39E-01 | 2.73E-02 | 3.56E-01 | 5.74E-01 | 9.49E-01 | 7.29E-01 | 7.28E-01 | 1.23E-01 |
| rs6467157 | | 7 | 127660763 | 1.43E-01 | 6.93E-01 | 9.05E-01 | 5.88E-02 | 1.04E-01 | 2.25E-02 | 3.06E-01 | 5.36E-01 | 7.76E-01 | 7.24E-01 | 7.20E-01 | 1.33E-01 |
| rs3918226 | | 7 | 150690176 | 6.69E-01 | 8.72E-01 | 4.70E-01 | 1.55E-02 | - | 6.04E-01 | - | 3.29E-03 | 5.02E-02 | 9.57E-01 | 3.02E-01 | 1.96E-01 |
| rs62496903 | | 8 | 6446938 | 3.52E-01 | 2.89E-01 | - | 4.11E-01 | - | 2.50E-01 | - | 4.29E-01 | 5.87E-01 | 4.07E-01 | 5.90E-01 | 5.57E-01 |
| rs732563 | | 8 | 23345526 | 5.67E-01 | 4.51E-01 | 1.44E-01 | 7.73E-01 | 3.43E-01 | 7.57E-01 | 3.90E-01 | 7.99E-01 | 6.36E-01 | 8.75E-01 | 2.28E-02 | 7.16E-01 |
| rs11778247 | | 8 | 23403378 | 4.41E-01 | 1.21E-01 | - | 3.61E-01 | - | 2.14E-03 | - | 1.88E-01 | 2.26E-01 | 5.15E-01 | 4.88E-01 | 6.13E-01 |
| rs34036147 | | 8 | 38366249 | 7.81E-01 | 5.10E-01 | - | 2.25E-01 | - | 6.95E-01 | - | 4.50E-02 | 1.17E-01 | 4.29E-01 | 5.17E-01 | 2.28E-01 |
| rs13266210 | | 8 | 41533514 | 4.55E-03 | 3.98E-01 | 3.49E-01 | 1.98E-01 | 2.02E-01 | 5.73E-01 | 7.06E-02 | 9.56E-02 | 1.25E-01 | 6.33E-01 | 7.10E-01 | 5.50E-01 |
| rs72656010 | | 8 | 57122215 | 1.27E-01 | 8.95E-02 | - | 1.18E-01 | - | 9.26E-01 | - | 5.64E-01 | 4.96E-01 | 4.04E-01 | 3.14E-02 | 6.54E-03 |
| rs6995390 | | 8 | 77611012 | 1.54E-01 | 4.67E-02 | 2.33E-01 | 2.45E-01 | 3.87E-01 | 1.42E-01 | 7.28E-01 | 1.50E-02 | 2.18E-01 | 3.96E-01 | 1.95E-01 | 8.72E-01 |
| rs7819593 | | 8 | 106115172 | 4.34E-01 | 9.31E-01 | 5.61E-01 | 1.65E-01 | 2.34E-01 | 9.27E-01 | 4.63E-02 | 1.80E-01 | 5.12E-01 | 8.74E-01 | 7.08E-01 | 3.53E-01 |
| rs10283100 | | 8 | 120596023 | 1.81E-01 | 9.45E-01 | 4.97E-01 | 6.53E-01 | 5.14E-01 | 6.03E-01 | 9.96E-01 | 9.49E-01 | 8.78E-01 | 8.66E-01 | 5.73E-01 | 6.78E-01 |
| rs13271368 | | 8 | 126506140 | 1.62E-01 | 5.36E-01 | - | 1.07E-01 | - | 5.29E-04 | - | 1.49E-01 | 5.10E-01 | 1.31E-01 | 2.85E-01 | 6.63E-01 |
| rs13257363 | | 8 | 142252580 | 5.23E-01 | 5.73E-01 | - | 1.72E-02 | - | 4.07E-01 | - | 5.89E-01 | 8.42E-01 | 3.82E-01 | 3.52E-02 | 6.68E-01 |
| rs9657468 | | 8 | 142362391 | 8.56E-02 | 2.31E-01 | - | 3.62E-01 | - | 9.74E-01 | - | 3.13E-01 | 2.74E-01 | 8.31E-01 | 5.94E-01 | 1.83E-01 |
| rs7854962 | | 9 | 96900505 | 9.33E-01 | 5.82E-01 | - | 6.90E-01 | - | 1.02E-01 | - | 5.12E-01 | 8.40E-01 | 8.47E-01 | 4.95E-01 | 4.35E-01 |
| rs28457693 | | 9 | 98217348 | 7.78E-01 | 8.17E-02 | - | 7.50E-02 | - | 6.28E-02 | - | 3.65E-02 | 7.55E-02 | 3.99E-01 | 3.83E-01 | 3.16E-02 |
| rs1411424 | | 9 | 113892963 | 5.62E-01 | 4.50E-01 | 9.35E-01 | 2.29E-01 | 6.45E-01 | 5.30E-01 | 8.96E-01 | 5.18E-01 | 6.76E-01 | 9.38E-01 | 4.34E-02 | 1.82E-01 |
| rs2418135 | | 9 | 113901309 | 7.05E-01 | 3.87E-01 | 8.71E-01 | 2.28E-01 | 8.11E-01 | 5.52E-01 | 9.60E-01 | 1.86E-01 | 2.19E-01 | 6.40E-01 | 8.33E-02 | 1.67E-01 |
| rs72760655 | | 9 | 116916214 | 6.50E-02 | 2.58E-01 | - | 7.64E-02 | - | 5.82E-01 | - | 1.86E-02 | 1.86E-01 | 6.44E-01 | 2.92E-01 | 9.90E-01 |
| rs1323438 | | 9 | 119115531 | 7.82E-01 | 2.67E-01 | 5.30E-02 | 4.06E-01 | 7.64E-01 | 5.57E-01 | 9.36E-01 | 9.81E-01 | 8.62E-01 | 9.20E-01 | 4.08E-01 | 7.43E-01 |
| rs3933326 | | 9 | 123633948 | 7.49E-01 | 3.22E-01 | 4.75E-01 | 6.16E-01 | 8.97E-01 | 6.19E-01 | 9.00E-01 | 7.23E-01 | 6.89E-01 | 6.88E-01 | 7.29E-01 | 2.31E-03 |
| rs10985827 | | 9 | 125701608 | 5.70E-01 | 6.70E-01 | - | 7.85E-01 | - | 1.43E-03 | - | 8.13E-01 | 6.83E-01 | 7.54E-01 | - | 2.10E-01 |
| rs28505901 | | 9 | 139241030 | 2.34E-01 | - | - | 8.77E-01 | - | 6.40E-01 | - | 4.92E-04 | 8.19E-05 | 2.44E-02 | 1.27E-01 | 1.88E-01 |
| rs4350272 | | 10 | 25056118 | 8.95E-01 | 2.04E-02 | 8.38E-02 | 3.19E-01 | 4.32E-01 | 5.93E-01 | 8.03E-01 | 7.18E-03 | 1.07E-03 | 2.47E-02 | 3.26E-01 | 4.92E-02 |
| rs5030938 | | 10 | 70975916 | 1.31E-01 | 3.83E-01 | 6.61E-01 | 4.53E-01 | 5.49E-01 | 6.39E-01 | 7.08E-01 | 6.99E-01 | 8.17E-01 | 4.41E-01 | 2.21E-04 | 8.13E-01 |
| rs9645500 | | 10 | 70986723 | 2.01E-01 | 3.71E-01 | 6.28E-01 | 3.51E-01 | 6.89E-01 | 6.00E-01 | 9.70E-01 | 8.05E-01 | 7.53E-01 | 4.80E-01 | 1.43E-04 | 9.39E-01 |
| rs1112718 | | 10 | 94479107 | 1.55E-01 | 5.36E-01 | - | 8.26E-01 | - | 2.99E-02 | - | 4.60E-04 | 2.46E-04 | 2.68E-02 | 4.68E-04 | 1.70E-01 |
| rs10509669 | | 10 | 95969913 | 1.87E-01 | 8.75E-01 | 3.46E-02 | 9.81E-01 | 3.66E-01 | 4.15E-01 | 9.45E-01 | 1.75E-01 | 7.76E-01 | 1.63E-01 | 5.21E-01 | 9.66E-01 |
| rs3740360 | | 10 | 96025491 | 1.21E-01 | 3.72E-02 | 3.67E-01 | 9.70E-02 | 5.19E-01 | 2.61E-01 | 1.40E-01 | 4.77E-01 | 9.72E-02 | 5.81E-01 | 9.30E-01 | 6.36E-01 |
| rs2274224 | | 10 | 96039597 | 4.59E-01 | 1.75E-01 | 8.68E-03 | 8.84E-01 | 8.72E-01 | 5.48E-01 | 3.13E-01 | 1.95E-01 | 3.14E-01 | 4.99E-01 | 1.51E-01 | - |
| rs562974282 | | 10 | 104201070 | - | - | - | 9.31E-01 | - | 4.53E-02 | - | - | - | - | 7.58E-01 | - |
| rs10883846 | | 10 | 104958244 | 6.42E-01 | 9.60E-01 | - | 9.54E-01 | - | 4.36E-02 | - | 3.73E-02 | 4.63E-01 | 2.33E-01 | 3.28E-01 | 2.26E-01 |
| rs7903146 | | 10 | 114758349 | 7.13E-01 | 9.18E-02 | 1.11E-11 | 2.31E-01 | 9.59E-01 | 5.34E-01 | 4.44E-01 | 5.48E-02 | 5.11E-02 | 2.26E-01 | 5.96E-01 | 7.41E-03 |
| rs7076938 | | 10 | 115789375 | 2.85E-01 | 4.30E-01 | 2.83E-01 | 7.45E-01 | 4.97E-01 | 9.23E-01 | 4.41E-01 | 5.35E-01 | 5.06E-01 | 8.23E-01 | 7.06E-01 | 2.30E-01 |
| rs1801253 | | 10 | 115805056 | 3.15E-01 | 5.36E-01 | 1.94E-01 | 6.31E-01 | 3.80E-01 | 9.50E-01 | 4.04E-01 | 4.35E-01 | 4.00E-01 | 8.24E-01 | 7.91E-01 | 2.35E-01 |
| rs71486610 | | 10 | 124134803 | 8.58E-01 | 4.17E-01 | - | 4.74E-01 | - | 8.63E-01 | - | 4.96E-01 | 2.36E-02 | 1.92E-02 | 2.81E-02 | 8.14E-04 |
| rs11042596 | | 11 | 2118860 | 8.23E-01 | 7.24E-01 | - | 3.97E-01 | - | 1.77E-01 | - | 5.37E-03 | 8.97E-03 | 3.03E-01 | 2.18E-01 | 1.12E-02 |
| rs234864 | | 11 | 2857297 | 7.93E-01 | 6.57E-01 | - | 7.92E-01 | - | 4.32E-01 | - | 6.32E-01 | 2.66E-01 | 4.55E-01 | 3.36E-01 | 9.85E-01 |
| rs2168101 | | 11 | 8255408 | 6.91E-01 | 1.96E-01 | 3.81E-01 | 9.52E-01 | 9.50E-01 | 2.80E-01 | - | 1.27E-01 | 4.00E-01 | 5.78E-01 | 9.87E-04 | 6.13E-01 |
| rs4444073 | | 11 | 10331664 | 1.30E-01 | 1.36E-01 | 5.47E-01 | 4.14E-01 | 1.10E-01 | 2.88E-01 | 1.21E-01 | 7.81E-01 | 1.95E-01 | 3.03E-01 | 6.49E-02 | 4.69E-02 |
| rs12574749 | | 11 | 32405355 | 9.76E-01 | 3.70E-02 | 1.28E-01 | 8.77E-01 | 8.74E-01 | 1.24E-02 | 8.37E-01 | 7.05E-01 | 8.94E-01 | 9.20E-01 | 2.40E-01 | 9.18E-02 |
| rs5030317 | | 11 | 32410337 | 9.06E-01 | 3.54E-01 | 2.36E-01 | 9.94E-01 | 3.74E-01 | 1.96E-02 | 8.32E-01 | 8.95E-01 | 7.40E-01 | 9.17E-01 | 1.55E-01 | 7.26E-02 |
| rs10437653 | | 11 | 46297631 | 8.56E-02 | 2.72E-03 | 9.78E-01 | 7.86E-01 | 2.02E-01 | 2.82E-01 | 4.32E-01 | 7.00E-01 | 2.79E-01 | 5.35E-01 | 3.32E-03 | 3.58E-01 |
| rs10734564 | | 11 | 48160429 | 2.55E-01 | 2.87E-01 | - | 1.47E-01 | - | 9.39E-01 | - | 6.63E-01 | 2.32E-01 | 1.77E-01 | 5.46E-02 | 2.06E-01 |
| rs667515 | | 11 | 69449076 | 4.45E-01 | 5.37E-01 | 2.14E-04 | 2.36E-02 | 8.56E-02 | 2.52E-01 | 1.65E-01 | 9.19E-01 | 2.59E-01 | 7.33E-02 | 1.92E-01 | 2.46E-01 |
| rs61885091 | | 11 | 69791952 | 3.29E-01 | - | - | 6.57E-02 | - | 1.24E-01 | - | 7.31E-01 | 8.80E-01 | 1.81E-01 | 2.64E-01 | - |
| rs10830963 | | 11 | 92708710 | 4.13E-01 | 8.28E-01 | 8.77E-02 | 7.71E-01 | 2.37E-01 | 6.60E-01 | 1.32E-01 | 6.53E-01 | 3.99E-01 | 2.38E-01 | 4.26E-01 | 7.06E-01 |
| rs10895278 | | 11 | 102095335 | 8.29E-01 | 4.15E-01 | 3.17E-01 | 9.67E-01 | 2.97E-01 | 5.12E-02 | 5.78E-01 | 2.62E-01 | 2.36E-01 | 2.25E-01 | 4.18E-01 | 9.40E-01 |
| rs76895963 | | 12 | 4384844 | - | - | - | 8.07E-01 | - | 2.38E-01 | - | 1.30E-01 | 2.56E-01 | 6.55E-01 | 3.21E-03 | - |
| rs11055030 | | 12 | 12878349 | 7.23E-01 | 1.56E-01 | - | 2.46E-01 | - | 6.32E-02 | - | 1.87E-01 | 1.01E-01 | 4.22E-01 | 7.10E-01 | 5.10E-01 |
| rs2306547 | | 12 | 26877885 | 8.55E-01 | 8.15E-01 | 2.34E-01 | 7.34E-01 | 6.03E-02 | 2.21E-01 | 3.75E-01 | 3.11E-01 | 4.61E-01 | 9.29E-01 | 9.44E-01 | 4.72E-01 |
| rs11051061 | | 12 | 30914668 | 9.48E-01 | 6.34E-02 | 3.53E-01 | 3.41E-01 | 1.12E-01 | 7.57E-01 | 5.17E-02 | 4.94E-01 | 1.41E-01 | 2.52E-01 | 3.17E-01 | - |
| rs6582623 | | 12 | 46613394 | 7.86E-01 | 8.66E-01 | 2.69E-01 | 3.65E-01 | 6.55E-01 | 2.61E-01 | 5.77E-01 | 1.28E-01 | 4.19E-01 | 9.64E-01 | 9.39E-01 | 2.30E-02 |
| rs180438 | | 12 | 47187260 | 9.13E-01 | 9.24E-01 | 6.70E-01 | 4.16E-01 | 5.72E-01 | 3.45E-01 | 5.14E-01 | 4.08E-01 | 9.86E-01 | 2.05E-01 | 1.89E-01 | 1.23E-01 |
| rs8756 | | 12 | 66359752 | 3.45E-01 | 2.73E-02 | 3.43E-03 | 2.27E-01 | 2.36E-02 | 9.39E-02 | 1.75E-03 | 6.35E-01 | 8.00E-01 | 6.46E-01 | 7.01E-02 | 6.25E-02 |
| rs7968682 | | 12 | 66371880 | 4.65E-01 | 2.98E-02 | 5.21E-03 | 3.30E-01 | 8.62E-02 | 9.04E-02 | 1.68E-03 | 6.82E-01 | 8.57E-01 | 6.63E-01 | 7.30E-02 | 6.84E-02 |
| rs1480470 | | 12 | 66412130 | 3.29E-01 | 1.49E-01 | - | 6.00E-01 | - | 7.22E-01 | - | 6.22E-01 | 6.51E-01 | 8.63E-01 | 8.38E-01 | 7.71E-01 |
| rs1533688 | | 12 | 102772745 | 8.83E-01 | 4.89E-01 | - | 7.56E-01 | - | 1.95E-01 | - | 4.11E-01 | 4.45E-01 | 9.11E-01 | 3.45E-01 | 3.79E-03 |
| rs2647873 | | 12 | 103081192 | 8.10E-01 | 5.22E-01 | 8.24E-02 | 1.77E-01 | 1.85E-01 | 8.91E-01 | 1.16E-01 | 9.20E-01 | 6.53E-01 | 6.34E-01 | 9.24E-01 | 5.26E-01 |
| rs17033114 | | 12 | 103123339 | 1.29E-01 | 8.22E-01 | 5.36E-01 | 4.68E-01 | 2.18E-01 | 5.40E-01 | 6.50E-01 | 6.22E-01 | 2.64E-01 | 3.56E-01 | 1.55E-01 | 3.37E-01 |
| rs3184504 | | 12 | 111884608 | 7.00E-02 | 1.14E-02 | 9.35E-06 | 1.48E-01 | 9.07E-02 | 9.33E-01 | 3.61E-02 | 1.24E-03 | 2.00E-05 | 2.05E-03 | 1.50E-01 | 3.02E-07 |
| rs9549046 | | 13 | 40647206 | 7.81E-01 | 1.07E-01 | - | 2.44E-01 | - | 4.30E-01 | - | 4.73E-01 | 6.46E-01 | 5.81E-01 | 8.96E-01 | 1.70E-01 |
| rs34217484 | | 13 | 48854550 | 3.98E-01 | 2.60E-01 | - | 2.70E-01 | - | 7.67E-01 | - | 5.38E-01 | 4.65E-01 | 9.49E-01 | 5.64E-01 | 9.78E-01 |
| rs9318511 | | 13 | 78601413 | 6.41E-02 | 7.16E-01 | - | 3.81E-01 | - | 9.82E-02 | - | 9.63E-01 | 7.08E-01 | 2.34E-01 | 2.05E-01 | 1.48E-03 |
| rs72681869 | | 14 | 50655357 | - | - | - | 3.31E-01 | - | 5.22E-01 | - | 8.26E-01 | 7.63E-01 | 2.55E-01 | 4.00E-06 | - |
| rs6575803 | | 14 | 101257755 | 8.14E-01 | 9.53E-01 | 7.78E-01 | 6.43E-01 | - | 5.47E-01 | - | 6.78E-01 | 9.67E-01 | 8.58E-01 | 3.97E-01 | 9.82E-01 |
| rs75844534 | | 15 | 38667117 | 8.63E-01 | 1.06E-01 | - | 4.54E-02 | - | 6.00E-01 | - | 4.69E-01 | 3.54E-01 | 1.91E-01 | 3.33E-01 | - |
| rs2928148 | | 15 | 41401550 | 9.15E-01 | 6.59E-01 | 9.78E-01 | 1.74E-01 | 1.53E-01 | 2.74E-04 | 1.35E-01 | 1.88E-02 | 3.86E-02 | 9.42E-01 | 1.21E-02 | 3.08E-01 |
| rs339969 | | 15 | 60883281 | 9.25E-01 | 8.77E-01 | 2.70E-03 | 1.12E-01 | 7.59E-01 | 1.76E-02 | 9.58E-01 | 3.24E-01 | 7.40E-01 | 6.63E-01 | 1.30E-02 | 9.95E-01 |
| rs3784789 | | 15 | 75082552 | 6.45E-01 | 1.08E-01 | 1.40E-03 | 9.56E-02 | 1.08E-01 | 1.12E-03 | 8.02E-03 | 2.38E-01 | 8.93E-02 | 2.05E-01 | 1.87E-01 | 5.47E-01 |
| rs12909648 | | 15 | 86224570 | 7.02E-01 | 9.21E-01 | 6.46E-01 | 1.39E-02 | 7.17E-02 | 5.81E-02 | 2.50E-01 | 1.14E-02 | 9.59E-02 | 8.19E-01 | 6.34E-01 | 2.57E-01 |
| rs12443252 | | 15 | 91064690 | 4.57E-01 | 8.71E-01 | - | 9.50E-01 | - | 9.15E-01 | - | 2.03E-01 | 5.53E-01 | 6.18E-01 | 1.27E-02 | 1.25E-01 |
| rs7183988 | | 15 | 91428589 | 8.71E-01 | 6.03E-04 | - | 1.08E-01 | - | 2.15E-02 | - | 3.06E-02 | 1.49E-02 | 1.30E-01 | 8.24E-04 | 9.61E-01 |
| rs4932373 | | 15 | 91429287 | 2.60E-01 | 1.06E-03 | - | 9.20E-01 | - | 9.42E-02 | - | 7.77E-03 | 3.32E-04 | 1.18E-03 | 4.60E-03 | 3.46E-01 |
| rs55958435 | | 15 | 96852638 | 4.88E-03 | 5.32E-01 | - | 3.37E-01 | - | 7.33E-01 | - | 3.78E-01 | 1.45E-01 | 1.26E-01 | 6.20E-01 | 6.30E-01 |
| rs7402983 | | 15 | 99193276 | 2.98E-01 | 4.68E-01 | - | 7.35E-03 | - | 4.93E-02 | - | 1.60E-01 | 2.43E-01 | 7.46E-01 | 8.86E-01 | 5.16E-01 |
| rs11630479 | | 15 | 99240481 | 9.45E-01 | 4.53E-01 | 9.43E-01 | 2.09E-02 | 9.73E-01 | 1.81E-01 | 4.56E-01 | 6.18E-01 | 7.04E-01 | 8.82E-01 | 4.27E-01 | 1.11E-01 |
| rs2045457 | | 16 | 20046115 | 7.19E-01 | 9.86E-01 | 1.71E-05 | 4.25E-01 | 5.50E-01 | 1.74E-04 | 8.46E-01 | 7.85E-05 | 2.92E-02 | 5.95E-01 | 9.18E-02 | 8.92E-01 |
| rs40434 | | 16 | 55699525 | 5.46E-01 | 2.64E-01 | - | 3.68E-01 | - | 4.63E-01 | - | 8.86E-01 | 9.41E-01 | 2.62E-01 | 8.36E-03 | 7.59E-01 |
| rs28544888 | | 16 | 55741204 | 5.89E-01 | 8.15E-01 | - | 7.23E-02 | - | 6.75E-01 | - | 9.83E-01 | 7.49E-01 | 2.58E-01 | 6.60E-01 | 6.54E-01 |
| rs11641308 | | 16 | 75312023 | 9.96E-01 | 4.92E-01 | - | 8.02E-01 | - | 3.87E-02 | - | 7.30E-03 | 4.26E-03 | 5.14E-02 | 4.95E-01 | 8.58E-02 |
| rs222857 | | 17 | 7164563 | 9.89E-01 | 2.00E-02 | 1.34E-01 | 5.13E-01 | 9.03E-01 | 2.74E-01 | 2.54E-01 | 4.26E-01 | 5.78E-01 | 9.62E-01 | 3.11E-01 | 8.64E-02 |
| rs2428362 | | 17 | 7180274 | 9.63E-01 | 2.05E-02 | - | - | - | - | - | 3.51E-01 | 6.95E-01 | 7.13E-01 | 6.57E-01 | 5.76E-02 |
| rs4511593 | | 17 | 7455536 | 2.30E-01 | 1.79E-01 | 5.49E-01 | 1.70E-01 | 6.04E-02 | 9.98E-01 | 2.55E-01 | 7.57E-01 | 2.72E-01 | 3.14E-01 | 9.09E-01 | 8.69E-01 |
| rs78378222 | | 17 | 7571752 | 9.47E-01 | 1.12E-01 | - | 1.11E-01 | - | 9.91E-01 | - | 3.82E-01 | 9.65E-01 | 1.68E-01 | 5.38E-02 | - |
| rs9909342 | | 17 | 25652275 | 4.46E-01 | 9.82E-01 | 3.30E-01 | 7.52E-01 | 4.57E-01 | 1.50E-01 | 2.00E-01 | 3.61E-01 | 5.91E-01 | 9.72E-01 | 8.17E-01 | 2.57E-01 |
| rs7223535 | | 17 | 29211667 | 5.67E-01 | 2.47E-02 | - | 8.05E-01 | - | 4.62E-01 | - | 7.58E-01 | 4.95E-01 | 3.01E-01 | 5.82E-01 | 2.50E-01 |
| rs11867479 | | 17 | 68090207 | 3.85E-01 | 7.42E-01 | 1.09E-01 | 1.65E-01 | 2.25E-01 | 3.30E-01 | 3.41E-01 | 9.95E-02 | 5.50E-02 | 3.63E-01 | 7.64E-01 | 1.74E-01 |
| rs10221267 | | 17 | 68464662 | 9.18E-01 | 7.17E-01 | - | 3.33E-02 | - | 8.64E-01 | - | 8.14E-01 | 5.91E-02 | 1.16E-02 | 4.98E-01 | 8.07E-01 |
| rs73354194 | | 17 | 79905947 | 8.63E-01 | - | - | 5.76E-01 | - | 7.35E-01 | - | 9.25E-01 | 7.01E-01 | 2.23E-01 | 7.38E-01 | - |
| rs9912553 | | 17 | 79959703 | 2.27E-01 | 1.47E-03 | - | 4.51E-01 | - | 1.18E-01 | - | 2.24E-01 | 1.75E-01 | 6.95E-01 | 6.96E-01 | 4.79E-01 |
| rs11082304 | | 18 | 20720973 | 3.65E-01 | 2.04E-01 | 8.99E-02 | 3.41E-01 | 3.13E-01 | 1.41E-01 | 6.97E-01 | 9.99E-01 | 6.58E-01 | 5.45E-01 | 4.66E-01 | 1.27E-01 |
| rs2779165 | | 19 | 4915447 | 9.83E-01 | 1.15E-01 | 8.06E-01 | 5.56E-01 | 8.92E-01 | 5.96E-01 | 8.70E-01 | 4.04E-01 | 8.28E-01 | 5.17E-01 | 1.06E-01 | 1.56E-01 |
| rs8106042 | | 19 | 7161849 | 6.85E-01 | 1.98E-01 | - | 8.05E-01 | - | 4.57E-01 | - | 9.96E-01 | 4.39E-01 | 2.46E-01 | 2.10E-01 | 7.30E-02 |
| rs2967676 | | 19 | 8789666 | 6.53E-01 | 2.73E-01 | 2.66E-01 | 4.66E-01 | 6.86E-01 | 5.19E-01 | 3.77E-01 | 3.12E-02 | 1.68E-02 | 3.59E-01 | 9.15E-01 | 4.66E-01 |
| rs41355649 | | 19 | 33790556 | 1.42E-01 | 7.03E-01 | - | 4.59E-02 | - | 4.73E-02 | - | 7.39E-01 | 5.52E-01 | 2.73E-01 | 3.50E-01 | 2.33E-01 |
| rs1129156 | | 19 | 40719076 | 4.60E-01 | 3.67E-01 | 8.16E-01 | 7.08E-01 | 9.43E-01 | 2.89E-02 | 4.33E-01 | 3.30E-01 | 1.33E-01 | 4.30E-01 | 2.42E-01 | 9.42E-01 |
| rs147957154 | | 19 | 43431040 | 4.26E-01 | - | - | - | - | - | - | 5.22E-04 | 1.00E-03 | 2.75E-01 | 9.41E-01 | - |
| rs516246 | | 19 | 49206172 | 3.82E-03 | 3.20E-01 | 2.68E-02 | 3.71E-01 | 3.83E-03 | 1.19E-13 | 3.88E-03 | 3.64E-01 | 8.16E-05 | 1.21E-08 | 2.82E-01 | 8.84E-01 |
| rs255773 | | 19 | 54723546 | 7.79E-01 | - | - | 5.38E-01 | - | 9.17E-01 | - | 6.34E-01 | 9.96E-02 | 5.10E-02 | 9.05E-01 | 7.50E-01 |
| rs147110934 | | 19 | 55993436 | 6.51E-01 | 9.08E-01 | - | 9.83E-01 | - | 8.92E-02 | - | 5.87E-01 | 4.94E-01 | 7.49E-01 | 7.22E-01 | - |
| rs12461110 | | 19 | 56320663 | 9.89E-01 | 4.43E-01 | 2.62E-01 | 6.20E-01 | 6.60E-01 | 1.08E-01 | 5.16E-01 | 3.34E-01 | 3.30E-01 | 5.22E-01 | 7.90E-01 | 1.23E-01 |
| rs304001 | | 19 | 56423668 | 9.28E-01 | 3.29E-01 | 9.37E-01 | 7.17E-01 | 7.77E-01 | 2.75E-01 | 6.66E-01 | 2.92E-01 | 2.01E-01 | 3.20E-01 | 9.01E-01 | 3.03E-02 |
| rs6040076 | | 20 | 10658882 | 8.06E-01 | 2.15E-02 | - | 8.48E-01 | - | 1.94E-01 | - | 7.84E-02 | 5.90E-03 | 1.74E-02 | 4.41E-01 | 2.55E-01 |
| rs6033062 | | 20 | 11207419 | 1.50E-01 | 9.43E-01 | 7.46E-03 | 6.26E-02 | 2.92E-02 | 7.70E-01 | 1.31E-01 | 2.53E-01 | 8.99E-02 | 1.23E-01 | 9.33E-01 | 1.33E-02 |
| rs1203876 | | 20 | 22540915 | 9.48E-01 | 4.75E-02 | 4.22E-02 | 5.77E-01 | 8.96E-01 | 8.29E-01 | 4.98E-01 | 3.92E-01 | 4.79E-01 | 7.10E-01 | 1.67E-02 | 1.23E-01 |
| rs11698914 | | 20 | 31327144 | 9.54E-01 | 8.54E-01 | - | 2.12E-03 | - | 3.64E-03 | - | 9.65E-03 | 2.90E-02 | 2.11E-01 | 6.53E-01 | 5.34E-01 |
| rs181451002 | | 20 | 32466219 | 3.26E-01 | 1.86E-01 | - | 9.17E-01 | - | 9.37E-01 | - | 8.64E-01 | 4.30E-01 | 6.01E-01 | 4.63E-01 | - |
| rs2889874 | | 20 | 33715777 | 7.21E-01 | 7.96E-01 | - | 4.37E-01 | - | 1.12E-01 | - | 2.32E-01 | 5.23E-01 | 4.21E-01 | 7.73E-01 | 5.69E-01 |
| rs1012167 | | 20 | 39159119 | 2.45E-03 | 4.46E-04 | 8.03E-01 | 1.47E-01 | 1.72E-01 | 1.68E-01 | 5.11E-01 | 6.41E-01 | 5.55E-01 | 9.66E-01 | 4.00E-06 | 8.23E-01 |
| rs753381 | | 20 | 39797465 | 5.62E-01 | 8.22E-01 | 1.10E-02 | 1.79E-01 | 6.21E-01 | 9.76E-01 | 7.48E-01 | 9.18E-02 | 8.41E-01 | 2.47E-02 | 7.21E-02 | 2.31E-02 |
| rs6026449 | | 20 | 57272617 | 9.27E-01 | 2.04E-01 | - | 5.14E-01 | - | 6.50E-01 | - | 7.14E-01 | 4.83E-01 | 1.65E-01 | 4.01E-01 | 2.71E-01 |
| rs73143584 | | 20 | 62445702 | 3.03E-01 | 3.27E-01 | - | 6.49E-01 | - | 8.72E-02 | - | 3.54E-01 | 4.04E-01 | 7.97E-01 | 7.79E-01 | 9.96E-01 |
| rs2229742 | | 21 | 16339172 | 4.51E-01 | 7.51E-01 | 9.87E-03 | 2.60E-01 | 4.11E-01 | 4.21E-01 | 8.50E-01 | 1.36E-01 | 1.32E-02 | 1.03E-01 | - | 2.89E-01 |
| rs220193 | | 21 | 43581308 | 6.36E-01 | 7.14E-01 | - | 6.71E-01 | - | 6.38E-01 | - | 5.62E-01 | 2.06E-01 | 3.06E-01 | 1.16E-01 | 5.85E-01 |
| rs134594 | | 22 | 29468456 | 3.01E-01 | 6.50E-02 | 9.20E-01 | 3.03E-01 | 5.42E-01 | 5.97E-02 | 4.33E-01 | 2.79E-01 | 4.01E-01 | 8.18E-01 | 5.10E-01 | 3.81E-01 |
| rs41311445 | | 22 | 42070374 | 2.69E-01 | 3.35E-01 | - | 1.06E-01 | - | 5.45E-01 | - | 2.67E-01 | 5.41E-01 | 9.45E-01 | 1.08E-03 | 9.07E-02 |
| rs7285579 | | 22 | 46441980 | 6.07E-03 | 2.04E-01 | - | 5.47E-01 | - | 3.57E-01 | - | 2.05E-01 | 6.07E-02 | 9.10E-02 | 5.30E-01 | 4.38E-01 |

**AN**; Anorexia nervosa, **UC**; ulcerative colitis, **IBD**; inflammatory bowel disease, **CD**; crohn’s disease, **VD**; 25-hydroxyvitamin D, **RA;** rheumatoid arthritis

**41-49**; The references in the main text

Note: the red color stands for the significant associations of birth weight variants with potential risk factors

**Table S9.** The association between fetal SNPs determined birth weight and potential risk factors in UK biobank dataset.

|  |  | **63 autosomal SNPs only with fetal effect** | | | **104 autosomal SNPs** | | | **205 autosomal SNPs** | | |
| --- | --- | --- | --- | --- | --- | --- | --- | --- | --- | --- |
| **Risk factors** | **Outcome** | **Effect** | **SE** | ***P*** | **Effect** | **SE** | ***P*** | **Effect** | **SE** | ***P*** |
|  |  |  |  |  |  |  |  |  |  |  |
| Metabolic markers |  |  |  |  |  |  |  |  |  |  |
|  | Alkaline phosphatase | 0.40 | 1.11 | 0.71 | -0.09 | 0.93 | 0.92 | 0.16 | 0.82 | 0.85 |
|  | Vitamin D | 0.28 | 1.05 | 0.79 | -0.16 | 0.88 | 0.85 | -0.62 | 0.77 | 0.40 |
|  | Oestradiol | 7.61 | 52.63 | 0.88 | 18.77 | 43.81 | 0.66 | -8.32 | 38.17 | 0.83 |
|  | Calcium | -0.01 | 0.0005 | **0.01** | -0.008 | 0.004 | **0.03** | -0.009 | 0.03 | **0.005** |
|  |  |  |  |  |  |  |  |  |  |  |
| Inflammatory markers |  |  |  |  |  |  |  |  |  |  |
|  | C-reactive protein | -0.26 | 0.17 | 0.13 | -0.15 | 0.14 | 0.31 | -0.15 | 0.13 | 0.23 |
|  | Rheumatoid factor | -1.49 | 3.64 | 0.68 | -0.08 | 3.06 | 0.97 | 0.18 | 2.69 | 0.95 |
|  | Urea | -0.11 | 0.059 | 0.06 | -0.07 | 0.04 | 0.15 | -0.05 | 0.04 | 0.21 |

**Table S10.** The association between fetal SNPs determined birth weight and adult BMC, Bone area, BMCadjArea and BMD in UK biobank dataset (confounders were sex, age, weight, smoking, alcohol, physical activity and calcium level).

|  |  |  | **63 autosomal SNPs only with fetal effect** | | | **104 autosomal SNPs** | | | **205 autosomal SNPs** | | |
| --- | --- | --- | --- | --- | --- | --- | --- | --- | --- | --- | --- |
| **Measurement** | **Sites** | **Outcome** | **Effect** | **SE** | ***P*** | **Effect** | **SE** | ***P*** | **Effect** | **SE** | ***P*** |
| DXA |  |  |  |  |  |  |  |  |  |  |  |
|  | Lumbar spine (LS, N=19,851) |  |  |  |  |  |  |  |  |  |  |
|  |  | BMC | -0.0005 | 0.03 | 0.98 | -0.01 | 0.03 | 0.73 | 0.02 | 0.02 | 0.31 |
|  |  | Bone Area | 0.14 | 0.03 | 1.57×10^-5^ | 0.14 | 0.02 | 2.08×10^-7^ | -0.13 | 0.05 | 0.01 |
|  |  | BMD | -0.12 | 0.04 | 0.007 | -0.13 | 0.03 | 0.0004 | -0.09 | 0.03 | 0.004 |
|  |  | BMCadjArea | -0.21 | 0.05 | 5.94×10^-5^ | -0.22 | 0.04 | 1.57×10^-7^ | -0.19 | 0.03 | 3.47×10^-7^ |
|  | Lateral spine  (LaS, N=20,874) |  |  |  |  |  |  |  |  |  |  |
|  |  | BMC | -0.07 | 0.03 | 0.02 | -0.07 | 0.02 | 0.01 | -0.04 | 0.02 | 0.08 |
|  |  | Bone Area | 0.01 | 0.03 | 0.71 | 0.03 | 0.03 | 0.24 | 0.04 | 0.02 | 0.16 |
|  |  | BMD | -0.14 | 0.03 | 0.0002 | -0.14 | 0.03 | 7.69×10^-6^ | -0.10 | 0.02 | 0.0001 |
|  |  | BMCadjArea | -0.18 | 0.04 | 0.0001 | -0.21 | 0.04 | 1.57×10^-5^ | -0.16 | 0.03 | 4.07×10^-6^ |
| QUS |  |  |  |  |  |  |  |  |  |  |  |
|  | Heel (N=173,874) |  |  |  |  |  |  |  |  |  |  |
|  |  | Heel BMD | -0.12 | 0.04 | 0.007 | -0.13 | 0.03 | 0.0004 | -0.09 | 0.03 | 0.004 |

All analyses were adjusted for sex, age, weight, smoking, alcohol and physical activity, calcium level.

**Table S11.** Causal associations of the fetal genotype effects on birth weight (63, 104, 205 instrumental variables) with hip bone parameters in two-sample MR analyses.

| **Outcome** | **N** | **Method** | **Estimate** | **Se** | **P value** | **Q' P value** | **Power (%)** |
| --- | --- | --- | --- | --- | --- | --- | --- |
|  |  | IVW method | -0.10 | 0.05 | 0.07 | < 0.001 | 100 |
|  | 63 | MR-Egger Intercept | -0.001 | 0.004 | 0.62 |  |  |
|  |  | MR-Egger | -0.03 | 0.15 | 0.83 |  |  |
|  |  | MR-PRESSO | -0.09 | 0.05 | 0.06 | 0.94 |  |
|  |  | IVW method | -0.09 | 0.05 | **0.06** | < 0.001 | 100 |
| **Femoral neck BMD** | 104 | MR-Egger Intercept | -0.002 | 0.002 | 0.46 |  |  |
|  |  | MR-Egger | -0.02 | 0.11 | 0.87 |  |  |
|  |  | MR-PRESSO | -0.07 | 0.04 | 0.06 | 0.66 |  |
|  |  | IVW method | -0.09 | 0.04 | **0.03** | < 0.001 | 100 |
|  | 205 | MR-Egger Intercept | 0.001 | 0.002 | 0.52 |  |  |
|  |  | MR-Egger | -0.14 | 0.09 | 0.13 |  |  |
|  |  | MR-PRESSO | -0.07 | 0.03 | **0.05** | 0.59 |  |
|  |  | IVW method | 0.18 | 0.13 | **0.002** | < 0.001 | 100 |
|  | 63 | MR-Egger Intercept | -0.006 | 0.003 | 0.11 |  |  |
|  |  | MR-Egger | 0.39 | 0.14 | 0.009 |  |  |
|  |  | MR-PRESSO | 0.15 | 0.05 | **0.005** | 0.62 |  |
|  |  | IVW method | 0.17 | 0.05 | **0.001** | < 0.001 | 100 |
| **Total hip area** | 104 | MR-Egger Intercept | 0.002 | 0.003 | 0.38 |  |  |
|  |  | MR-Egger | 0.28 | 0.13 | 0.03 |  |  |
|  |  | MR-PRESSO | 0.16 | 0.05 | **0.002** | 0.73 |  |
|  |  | IVW method | 0.17 | 0.10 | **0.0003** | < 0.001 | 100 |
|  | 205 | MR-Egger Intercept | -0.005 | 0.002 | 0.33 |  |  |
|  |  | MR-Egger | 0.37 | 0.10 | 0.0003 |  |  |
|  |  | MR-PRESSO | 0.16 | 0.04 | **0.0001** | 0.85 |  |
|  |  | IVW method | 0.05 | 0.06 | 0.43 | < 0.001 | 95.9 |
|  | 63 | MR-Egger Intercept | -0.004 | 0.004 | 0.36 |  |  |
|  |  | MR-Egger | 0.20 | 0.18 | 0.26 |  |  |
|  |  | MR-PRESSO | 0.05 | 0.06 | 0.48 | 0.91 |  |
|  |  | IVW method | 0.10 | 0.06 | 0.09 | < 0.001 | 94 |
| **Femoral neck area** | 104 | MR-Egger Intercept | 0.002 | 0.003 | 0.47 |  |  |
|  |  | MR-Egger | 0.01 | 0.14 | 0.91 |  |  |
|  |  | MR-PRESSO | 0.04 | 0.05 | 0.43 | 0.10 |  |
|  |  | IVW method | 0.10 | 0.04 | **0.01** | < 0.001 | 100 |
|  | 205 | MR-Egger Intercept | -0.002 | 0.002 | 0.38 |  |  |
|  |  | MR-Egger | 0.19 | 0.10 | 0.07 |  |  |
|  |  | MR-PRESSO | 0.08 | 0.04 | **0.04** | 0.38 |  |
|  |  | IVW method | 0.14 | 0.04 | **0.002** | < 0.001 | 100 |
|  | 63 | MR-Egger Intercept | -0.005 | 0.003 | 0.08 |  |  |
|  |  | MR-Egger | 0.35 | 0.12 | 0.006 |  |  |
|  |  | MR-PRESSO | 0.13 | 0.04 | **0.0009** | 0.68 |  |
|  |  | IVW method | 0.13 | 0.04 | **0.004** | < 0.001 | 100 |
| **Intertrochanteric area** | 104 | MR-Egger Intercept | -0.002 | 0.002 | 0.26 |  |  |
|  |  | MR-Egger | 0.24 | 0.10 | 0.02 |  |  |
|  |  | MR-PRESSO | 0.13 | 0.04 | **0.001** | 0.89 |  |
|  |  | IVW method | 0.12 | 0.04 | **0.002** | < 0.001 | 100 |
|  | 205 | MR-Egger Intercept | -0.003 | 0.002 | 0.09 |  |  |
|  |  | MR-Egger | 0.26 | 0.09 | 0.004 |  |  |
|  |  | MR-PRESSO | 0.14 | 0.03 | **0.0001** | 0.60 |  |
|  |  | IVW method | 0.10 | 0.05 | 0.06 | < 0.001 | 100 |
|  | 63 | MR-Egger Intercept | -0.002 | 0.003 | 0.47 |  |  |
|  |  | MR-Egger | 0.19 | 0.14 | 0.17 |  |  |
|  |  | MR-PRESSO | 0.11 | 0.05 | **0.03** | 0.56 |  |
|  |  | IVW method | 0.11 | 0.05 | **0.02** | < 0.001 | 100 |
| **Trochanter area** | 104 | MR-Egger Intercept | -0.002 | 0.002 | 0.39 |  |  |
|  |  | MR-Egger | 0.20 | 0.11 | 0.08 |  |  |
|  |  | MR-PRESSO | 0.14 | 0.04 | **0.005** | 0.65 |  |
|  |  | IVW method | 0.13 | 0.04 | **0.006** | < 0.001 | 100 |
|  | 205 | MR-Egger Intercept | -0.004 | 0.002 | 0.07 |  |  |
|  |  | MR-Egger | 0.30 | 0.10 | 0.005 |  |  |
|  |  | MR-PRESSO | 0.14 | 0.04 | **0.0006** | 0.69 |  |

**Table S12.** Causal associations of the fetal genotype effects on birth weight (62, 102, 200 instrumental variables which excluded the potential pleiotropic variants) with hip bone parameters in two-sample MR analyses.

| **Outcome** | **N** | **Method** | **Estimate** | **Se** | **P value** | **Q' P value** | **Power (%)** |
| --- | --- | --- | --- | --- | --- | --- | --- |
|  |  | IVW method | -0.09 | 0.05 | 0.10 | < 0.001 | 100 |
|  | 62 | MR-Egger Intercept | -0.001 | 0.004 | 0.78 |  |  |
|  |  | MR-Egger | -0.05 | 0.15 | 0.72 |  |  |
|  |  | MR-PRESSO | -0.09 | 0.05 | **0.09** | 0.95 |  |
|  |  | IVW method | -0.08 | 0.05 | **0.08** | < 0.001 | 100 |
| **Femoral neck BMD** | 102 | MR-Egger Intercept | -0.001 | 0.002 | 0.59 |  |  |
|  |  | MR-Egger | -0.03 | 0.11 | 0.79 |  |  |
|  |  | MR-PRESSO | -0.07 | 0.04 | **0.09** | 0.86 |  |
|  |  | IVW method | -0.08 | 0.04 | **0.03** | < 0.01 | 100 |
|  | 200 | MR-Egger Intercept | 0.001 | 0.002 | 0.57 |  |  |
|  |  | MR-Egger | -0.13 | 0.09 | 0.15 |  |  |
|  |  | MR-PRESSO | -0.07 | 0.03 | **0.05** | 0.69 |  |
|  |  | IVW method | 0.17 | 0.05 | **0.002** | 0.001 | 100 |
|  | 62 | MR-Egger Intercept | -0.006 | 0.003 | 0.12 |  |  |
|  |  | MR-Egger | 0.38 | 0.14 | 0.01 |  |  |
|  |  | MR-PRESSO | 0.15 | 0.05 | **0.006** | 0.61 |  |
|  |  | IVW method | 0.18 | 0.05 | **0.001** | < 0.001 | 100 |
| **Total hip area** | 102 | MR-Egger Intercept | -0.002 | 0.003 | 0.45 |  |  |
|  |  | MR-Egger | 0.26 | 0.12 | 0.03 |  |  |
|  |  | MR-PRESSO | 0.16 | 0.05 | **0.001** | 0.75 |  |
|  |  | IVW method | 0.18 | 0.04 | **0.00002** | < 0.001 | 100 |
|  | 200 | MR-Egger Intercept | -0.004 | 0.002 | 0.06 |  |  |
|  |  | MR-Egger | 0.36 | 0.10 | 0.0004 |  |  |
|  |  | MR-PRESSO | 0.18 | 0.04 | **0.00001** | 0.98 |  |
|  |  | IVW method | 0.06 | 0.06 | 0.35 | < 0.001 | 60.1 |
|  | 62 | MR-Egger Intercept | < 0.001 | 0.004 | 0.004 |  |  |
|  |  | MR-Egger | 0.17 | 0.17 | 0.31 |  |  |
|  |  | MR-PRESSO | 0.03 | 0.06 | 0.57 | 0.37 |  |
|  |  | IVW method | 0.11 | 0.06 | **0.07** | < 0.001 | 100 |
| **Femoral neck area** | 102 | MR-Egger Intercept | 0.003 | 0.006 | 0.38 |  |  |
|  |  | MR-Egger | 0.003 | 0.14 | 0.97 |  |  |
|  |  | MR-PRESSO | 0.08 | 0.05 | **0.09** | 0.45 |  |
|  |  | IVW method | 0.11 | 0.04 | **0.01** | < 0.001 | 100 |
|  | 200 | MR-Egger Intercept | -0.001 | 0.002 | 0.45 |  |  |
|  |  | MR-Egger | 0.18 | 0.10 | 0.08 |  |  |
|  |  | MR-PRESSO | 0.07 | 0.04 | **0.06** | 0.19 |  |
|  |  | IVW method | 0.15 | 0.04 | **0.002** | 0.10 | 100 |
|  | 62 | MR-Egger Intercept | -0.005 | 0.003 | 0.09 |  |  |
|  |  | MR-Egger | 0.35 | 0.12 | 0.007 |  |  |
|  |  | MR-PRESSO | - | - | - | - |  |
|  |  | IVW method | 0.13 | 0.04 | **0.003** | < 0.001 | 100 |
| **Intertrochanteric area** | 102 | MR-Egger Intercept | -0.002 | 0.002 | 0.34 |  |  |
|  |  | MR-Egger | 0.22 | 0.10 | 0.03 |  |  |
|  |  | MR-PRESSO | 0.14 | 0.04 | **0.001** | 0.87 |  |
|  |  | IVW method | 0.13 | 0.03 | **0.0005** | < 0.001 | 100 |
|  | 200 | MR-Egger Intercept | -0.002 | 0.002 | 0.16 |  |  |
|  |  | MR-Egger | 0.25 | 0.09 | 0.006 |  |  |
|  |  | MR-PRESSO | 0.14 | 0.03 | **0.0001** | 0.80 |  |
|  |  | IVW method | 0.09 | 0.05 | **0.07** | 0.005 | 100 |
|  | 62 | MR-Egger Intercept | -0.003 | 0.003 | 0.43 |  |  |
|  |  | MR-Egger | 0.20 | 0.14 | 0.16 |  |  |
|  |  | MR-PRESSO | 0.11 | 0.05 | **0.03** | 0.78 |  |
|  |  | IVW method | 0.11 | 0.05 | **0.03** | < 0.01 | 100 |
| **Trochanter area** | 102 | MR-Egger Intercept | -0.003 | 0.003 | 0.38 |  |  |
|  |  | MR-Egger | 0.20 | 0.12 | 0.08 |  |  |
|  |  | MR-PRESSO | 0.13 | 0.04 | **0.008** | 0.73 |  |
|  |  | IVW method | 0.12 | 0.04 | **0.006** | < 0.001 | 100 |
|  | 200 | MR-Egger Intercept | -0.004 | 0.002 | 0.08 |  |  |
|  |  | MR-Egger | 0.29 | 0.10 | 0.006 |  |  |
|  |  | MR-PRESSO | 0.15 | 0.04 | **0.0003** | 0.62 |  |
